# A comprehensive overview of radioguided surgery using gamma detection probe technology

**DOI:** 10.1186/1477-7819-7-11

**Published:** 2009-01-27

**Authors:** Stephen P Povoski, Ryan L Neff, Cathy M Mojzisik, David M O'Malley, George H Hinkle, Nathan C Hall, Douglas A Murrey, Michael V Knopp, Edward W Martin

**Affiliations:** 1 Division of Surgical Oncology, Department of Surgery, Arthur G. James Cancer Hospital and Richard J. Solove Research Institute and Comprehensive Cancer Center, The Ohio State University, Columbus, OH, 43210, USA; 2 Department of Radiology, The Ohio State University, Columbus, OH, 43210, USA; 3 Division of Gynecologic Oncology, Department of Obstetrics and Gynecology, Arthur G. James Cancer Hospital and Richard J. Solove Research Institute and Comprehensive Cancer Center, The Ohio State University, Columbus, OH, 43210, USA; 4 College of Pharmacy, The Ohio State University, Columbus, OH, 43210, USA

## Abstract

The concept of radioguided surgery, which was first developed some 60 years ago, involves the use of a radiation detection probe system for the intraoperative detection of radionuclides. The use of gamma detection probe technology in radioguided surgery has tremendously expanded and has evolved into what is now considered an established discipline within the practice of surgery, revolutionizing the surgical management of many malignancies, including breast cancer, melanoma, and colorectal cancer, as well as the surgical management of parathyroid disease. The impact of radioguided surgery on the surgical management of cancer patients includes providing vital and real-time information to the surgeon regarding the location and extent of disease, as well as regarding the assessment of surgical resection margins. Additionally, it has allowed the surgeon to minimize the surgical invasiveness of many diagnostic and therapeutic procedures, while still maintaining maximum benefit to the cancer patient. In the current review, we have attempted to comprehensively evaluate the history, technical aspects, and clinical applications of radioguided surgery using gamma detection probe technology.

## Background

The concept of radioguided surgery using a radiation detection probe system originated approximately 60 years ago. Interestingly, the first recognized description of radioguided surgery involving a radiation detection probe system [1] did not involve a gamma detection probe, but instead involved the use of a gaseous ionization detector called a Geiger-Müller tube [2], which has a high sensitivity for beta radiation emitting radionuclides and a very low sensitivity for gamma radiation emitting radionuclides.

In 1949, Selverstone et al [1] at Harvard Medical School reported on 33 suspected brain tumor patients that were intravenously injected with the beta radiation emitter, phosphorus-32 (^32^P). At surgery, using a handheld Geiger-Müller tube device, counts in the area of suspected tumor and normal brain tissue were obtained at various time intervals and various depths beneath the cerebral cortex. Following successful location of the tumor, attempts were made to demarcate its tumor boundary margins using the Geiger-Müller counter. Of 33 evaluated patients, 23 brain tumors (88%) were localized using the Geiger-Müller counter. In 12 patients, the Geiger-Müller counter was used to facilitate total extirpation of tumor. In four patients, tumor was not localized by means of the Geiger-Müller counter. This included two false-negative results that were attributed to the inability to place the Geiger-Müller counter in close proximity to the tumor, one patient with diffuse infiltration of the entire cerebral hemisphere with tumor that precluded distinguishing it from normal adjacent tissue, and one patient in which no tumor was correctly identified.

It was then not until 1956, when Harris et al [3] at the Oak Ridge Institute of Nuclear Studies Medical Hospital reported the first description of radioguided surgery involving a gamma detection probe system. In their published report, a patient with a history of thyroid cancer who had previously undergone a total thyroidectomy some three years earlier and who had persistent iodine uptake in the neck region was intravenously injected with the gamma radiation emitter, iodine-131 (^131^I). At surgery, using a handheld scintillation detector device as the gamma detection probe, they localized and successfully resected an area of residual thyroid tissue.

Since the time of these landmark reports by Selverstone et al [1] and Harris et al [3], the concept of radioguided surgery and its supporting technologies has tremendously expanded and has evolved into what is now considered an established discipline within the practice of surgery, revolutionizing the surgical management of many malignancies. Along the way, various milestones in radioguided surgery have been reached (Table 1), and the clinical application of this technology has, to varying degrees, impacted upon almost every facet of cancer-related surgery (Table 2). The impact of radioguided surgery on the surgical management of cancer patients includes providing vital and real-time information to the surgeon regarding the location and extent of disease, as well as regarding the assessment of surgical resection margins. Additionally, it has allowed the surgeon to minimize the surgical invasiveness of many diagnostic and therapeutic procedures, while still maintaining maximum benefit to the cancer patient.

**Table 1:** Historical timeline for milestones in radioguided surgery

Year	Milestone
1949	Selverstone et al [1] at Harvard Medical School (Boston, Massachusetts, USA) were the very first to report the concept of radioguided surgery using of a Geiger-Müller tube device and ^32^P to detect brain tumors.
1956	Harris et al [3] at the Oak Ridge Institute of Nuclear Studies Medical Hospital (Oak Ridge, Tennessee, USA) were the first to report the application of a gamma detection probe during radioguided surgery using ^131^I to detect residual thyroid tissue.
1981	Harvey et al [743] at Presbyterian Hospital of Dallas (Dallas, Texas, USA) first reported the application of a gamma detection probe for radioguided biopsy of benign and metastatic bone lesions using ^99m^Tc methylene diphosphonate.
1981	Ghelman et al [728] at The Hospital for Special Surgery (New York, New York, USA) first reported the application of a gamma detection probe for radioguided resection of a benign bone lesion using ^99m^Tc methylene diphosphonate.
1984	Aitken et al [289,290] at The Ohio State University (Columbus, Ohio, USA) first reported radioimmunoguided surgery using ^131^I-labeled anti-CEA polyclonal antibody to detect colorectal cancer.
1984	Ubhi et al. [408] at Queen's Medical Center/University Hospital (Nottingham, England, UK) first reported radioguided surgery using ^201^Tl-thallous chloride for the detecting a parathyroid adenoma.
1987	Sickle-Santanello et al [299] at The Ohio State University (Columbus, Ohio, USA) first reported radioimmunoguided surgery using ^125^I-labeled anti-TAG-72 monoclonal antibody to detect colorectal cancer.
1993	Krag et al [135] at The University of Vermont (Burlington, Vermont, USA) first reported radioguided sentinel lymph node biopsy using ^99m^Tc radiocolloid for breast cancer.
1993	Alex et al [188] at The University of Vermont (Burlington, Vermont, USA) first reported radioguided sentinel lymph node biopsy using ^99m^Tc radiocolloid for malignant melanoma.
1995	Martinez et al [409] at The Ohio State University (Columbus, Ohio, USA) first reported use of ^99m^Tc-MIBI for the detecting parathyroid gland pathology.
1997	Norman and Chheda [410] at The University of South Florida (Tampa, Florida, USA) popularized the technique of minimally-invasive radioguided surgery using ^99m^Tc-MIBI for the surgical management of primary hyperparathyroidism.
1999	Desai et al [35,36] at The Ohio State University (Columbus, Ohio, USA) first reported use of ^18^F-FDG-directed surgery in the surgical management of colorectal cancer.
2008	Strong et al [29] at Memorial Sloan-Kettering Cancer Center (New York, New York, USA) first reported radioimmunoguided surgery using ^124^I-labeled monoclonal antibody specific for clear cell renal cell cancer.

**Table 2:** Clinical applications of radioguided surgery using gamma detection probe technology

Clinical applications	Specific type(s) of radioguided surgery applications
Breast cancer	RGSLNB, RIGS, ROLL, RIME, FDGDS
Cutaneous malignancies	
Malignant melanoma	RGSLNB, FDGDS
Merkel cell carcinoma	RGSLNB
Other cutaneous malignancies	RGSLNB
Gastrointestinal malignancies	
Colorectal cancer	RIGS, RGSLNB, FDGDS
Anal cancer	RGSLNB
Esophageal cancer	RGSLNB
Gastric cancer	RGSLNB, RIGS, FDGDS
Pancreatic cancer	RIGS
GIST	FDGDS
Head and neck malignancies	
Squamous cell cancer	RGSNLB, RIGS, FDGDS
Parathyroid disease	RGS
Thyroid cancer	RGS, FDGDS, RGSLNB
Parotid gland cancer	RGSLNB
Gynecologic malignancies	
Vulvar cancer	RGSLNB
Vaginal carcinoma	RGSLNB
Cervical cancer	RGSLNB
Endometrial cancer	RGSLNB
Ovarian Cancer	RIGS, FDGDS
Urologic malignancies	
Penile cancer	RGSLNB
Prostate cancer	RGSLNB, RIGS
Testicular cancer	RGSLNB, FDGDS
Bladder cancer	RGSLNB
Renal cell cancer	RIGS
Thoracic malignancies	
Lung cancer	RGSLNB, RIGS, RGS, FDGDS
Pulmonary nodules	RGS
Neuroendocrine tumors	
GEP neuroendocrine tumors	RGS
Bronchial carcinoids	RGS
Neuroblastoma	RGS
Pheochromocytoma	RGS
Adrenocortical carcinoma	FDGDS
Sarcoma	RGSLNB
Brain tumors	RGS
Bone lesions	RGS
Lymphoma	RGS
Monitoring of isolated limb perfusion	RGS

RGS, radioguided surgery; RIGS, radioimmunoguided surgery; RGSLNB, radioguided sentinel lymph node biopsy; ROLL, radioguided occult lesion localization; RIME, radioguided intraoperative margins evaluation; FDGDS, ^18^F-FDG directed surgery; GIST, gastrointestinal stromal tumor; GEP, gastroenteropancreatic

## Gamma detection probe systems

Numerous handheld intraoperative radiation detection probe systems have been developed and have been made commercially available for use in radioguided surgery [4-23]. Such intraoperative radiation detection probes are divided into two general categories (i.e., gamma detection probes and beta detection probes), based upon the specific type of radiation detected. Gamma probes detect photon radiation, consisting of either gamma rays or x-rays [10,11,17]. Beta probes detect beta radiation, consisting of either positrons (positively charged electrons) or negatrons (negatively charged electrons) [10,11,18-23]. This includes some beta detection probe systems that are reported to have gamma photon background rejection capabilities [22,23]. However, the present review will specifically concentrate upon the use of gamma detection probe technology in radioguided surgery. Additionally, the present review will not specifically discuss or advocate the use of any individual commercially-available brand names of gamma detection probe technology.

### Important performance variables of gamma detection probe systems

The most important performance variables of any given gamma detection probe system consist of: (1) overall sensitivity (efficiency); (2) spatial selectivity (radial sensitivity distribution); (3) spatial resolution (lateral sensitivity distribution); (4) energy resolution (spectral discrimination); and (5) contrast [9,11,13-15,17]. Overall sensitivity (efficiency) is the detected count rate (photons detected) per unit of activity (photons emitted) and is determined at the tip of the probe profile. Spatial selectivity (radial sensitivity distribution) is described by the width of the resultant measurement cone out of which radiation is being detected at a defined distance. With a wider measurement cone, background signal may exceed target source signal and can lead to interference with detection of the target signal. With a narrower measurement cone, background counts will be reduced and detection of the target source signal will be more likely, even in the presence of an increased background signal or noise. Spatial resolution (lateral sensitivity distribution) is the ability of the gamma detection probe to accurately localize the position of a target source of activity, as well as to separate and distinguish two target sources of activity which are located relatively close to each other. Energy resolution (spectral discrimination) is the capacity of the gamma detection system to discriminate between emitted radiation of differing energies. Such energy discrimination is critical in two particular respects. First, it is critical for distinguishing two simultaneously administered radionuclides that have differing energies. Second, it is critical for distinguishing primary photons from scattered photons when higher-energy radionuclides are administered. Finally, contrast, which is directly related to all of the above performance variables of the gamma detection probe system, reflects the ability of the gamma detection probe to distinguish activity within the target tissue from that of the lower background activity within the surrounding non-target tissue.

### Basic principles of the radiation detector source housed within the gamma detection probe system

Two general categories of gamma detection probe systems exist that can be utilized within the operating room environment. These include gamma detection probe systems that utilize a scintillation detector and gamma detection probe systems that utilize a semiconductor ionization detector [4,6,8,10-12,15-17]. Only crystalline materials are used as the detector source within commercially-available gamma detection probes.

Crystalline materials that have been utilized in scintillation detectors include thallium-activated sodium iodide (NaI [Tl]), thallium-activated cesium iodide (CsI [Tl]), samarium-activated lutetium ortho-oxysilicate (LSO), and bismuth germanate (BGO). The basic principle behind how a scintillation-type detection system works is that the radiation emitted from the radionuclide excites atoms within the scintillation crystal and produces visible light in proportion to the energy absorbed. A photomultiplier tube is used to enhance the resultant visible light that is produced and is then converted into an electrical pulse that is collected by the detection unit. Crystalline materials that have been utilized in semiconductor ionization detectors include cadmium telluride (CdTe), cadmium zinc telluride (CdZnTe), and mercuric iodide (HgI_2_). The basic principle behind how a semiconductor ionization-type detection system works is that the radiation emitted from the radionuclide produces free electrons as it passes through and ionizes the semiconductor crystal. The resultant free electrons that are produced then create an electrical pulse that is collected and amplified by the detection unit.

There are advantageous and disadvantageous features that are specific to scintillation-type detection systems and to semiconductor ionization-type detection systems [4,6,8,10-12,15,17]. On one hand, scintillation-type detection systems have higher sensitivity (especially for medium-energy to high-energy gamma photons), but have poorer energy resolution and scatter rejection. Likewise, scintillation-type detection probes tend to have a much bulkier probe head profile design. On the other hand, semiconductor ionization-type detection systems have higher energy resolution and scatter rejection, but have lower sensitivity (especially for medium-energy to high-energy gamma photons). Likewise, semiconductor ionization-type detection probes tend to have a much more compact probe head profile design.

### Factors important in the appropriate selection of a gamma detection probe system for its intended clinical application

Several factors are important in the appropriate selection of a particular gamma detection probe system [4,5,8,9,11,15,17].

First, the specific radionuclide utilized and its particular gamma photon energy level is very important in the appropriate selection of a particular gamma detection probe system [8,11,17]. Whereas technetium-99m (^99m^Tc) labeled agents have been used almost exclusively for radioguided sentinel lymph node biopsy (SNL) procedures, various other radiopharmaceutical agents, such as monoclonal antibodies bound to various radionuclides (most commonly iodine radionuclides, indium-111 (^111^In), and ^99m^Tc), as well as fluorine-18 (^18^F) bound to a nonphysiologic analog of glucose have also been used in radioguided surgical resection of tumors. While most commercially available gamma detection probe systems have relatively high sensitivity (efficiency) for predominantly lower energy gamma photon emitting radionuclides (such as iodine-125 (^125^I) and ^99m^Tc), this may not necessarily be the case for predominantly higher energy gamma photon emitting radionuclides (such as ^131^I) or positron emitting radionuclides that produce high-energy gamma photons from resultant positron-electron annihilation (such as iodine-124 (^124^I) and ^18^F). As such, these resultant high-energy gamma photons remain an ongoing challenge for the gamma detection probe systems that are currently commercially available and has been the focus of recent product development of gamma detection probe systems that are specifically intended for the detection of high-energy gamma photons.

Second, the nature of the surgical procedure to be performed is important in the appropriate selection of a particular gamma detection probe system [8,9,11,15,17]. On one hand, gamma detection probe systems used for radioguided sentinel lymph node procedures require exceptional spatial resolution in order to allow for more precise localization of small lymph node candidates. On the other hand, gamma detection probe systems used for radioguided surgical resection of tumors requires high sensitivity in order to help guide the surgeon to the specific sites of disease while rapidly searching over a relatively large surgical field.

Third, the necessity for shielding and collimation of the head of the probe housing the crystalline material is also critical in the appropriate selection of a particular gamma detection probe system [4,5,8,9,11,15,17]. These features may already be built into the standard probe head or can be added onto the existing standard probe head. The function of shielding (material such as lead, tungsten, gold, or platinum) and collimation (length and aperture of the collimator) is to prevent attenuated radiation from unintended locations (i.e., scatter) from accessing the detector source within the probe head and thus producing unintended counts that are recognized by the gamma detection system. Side and back shielding of the probe head can be rather important when there is a strong and localized radiation source (i.e., the ^99m^Tc-labeled agent injection site for a radioguided SLN procedure) which lies in close proximity to the intended target (i.e., the SLN) or when utilizing higher energy gamma photon emitting radionuclides (such as ^131^I) or positron emitting radionuclides that produce high-energy gamma photons from resultant positron-electron annihilation (such as ^124^I and ^18^F). It is clear that collimation of the gamma detection probe head results in improved spatial resolution and contrast between the emitted radiation from the intended target as compared to emitted radiation from surrounding non-target tissue (especially in areas of higher background activity). However, at the same time, such collimation produces a resultant loss in the sensitivity of the gamma detection probe system by decreasing the effectual detection aperture and lengthening the distance to the actual detection source. Thicker shielding and/or longer collimation is generally necessary when using higher energy gamma photon producing radionuclides. However, the addition of thicker shielding and/or longer collimation will increase the overall weight and size-dimensions of the gamma detection probe.

### Desirable design features of any given gamma detection probe system that are important to the surgeon

Many design features of any given gamma detection probe system may be important to the surgeon [8,9,11,12,15,17]. The presence or absence of such specific design features may make any particular gamma detection probe system more or less attractive to the surgeon. First and foremost, the weight, shape, and ergonomic design of the gamma detection probe are critical. By far, surgeons favor sleekly designed, pencil-thin, lightweight probes and angulation of the detector head for better access to desired detection locations. While pencil-thin probes may offer higher spatial resolution secondary to their smaller detector size, they, unfortunately, yield a lower sensitivity than do larger-sized detector probes and can limit the degree of attainable shielding and collimation. Second, the audible signal and digital display of the gamma detection control unit are important variables for providing critical output information to the surgeon as to the localization of the radionuclide to the area of interest without distracting the surgeon from the overall activities within the surgical field [8]. Third, flexibility and adaptability of any given system with regards to removable side shielding, interchangeable collimators, interchangeable detection probes, and user-adjustable energy windows for different radionuclides is also critical to the overall design of a given gamma detection probe system. Lastly, the recent development of handheld, self-contained gamma detection probe systems [24], as well as wireless gamma detection probe technology that is adaptable to existing gamma detection probe systems [25] may help to further advance the technology involved in radioguided surgery by eliminating the need for cables within the surgical field that previously connected the gamma detection probe itself to the gamma detection control unit [25]. All these technology developments involving gamma detection probe systems may ultimately provide the surgeon with more flexibility for utilization of these innovative devices within the operating room environment.

## Properties of radionuclides utilized in radioguided surgery

Numerous radionuclides have been utilized with the gamma detection probe in radioguided surgery. This includes, in alphabetical order, cobalt-57 (^57^Co), ^18^F, gallium-67 (^67^Ga), ^111^In, iodine-123 (^123^I), ^124^I, ^125^I, ^131^I, ^99m^Tc, and thallium-201 (^201^Tl) [26]. The physical half-life, principle gamma photon radiation emission(s), and emission probability per decay (photon yield) of each of these radionuclides are summarized in Table 3[27]. In general, the gamma photon radiation emitted from each radionuclide, which is expressed in kiloelectron volts (keV), can be characterized as low-energy emission (0 keV to 150 keV), medium-energy emission (150 keV to 400 keV), or high-energy emission (greater than 400 keV). To date, the radionuclides that have been utilized most frequently with the gamma detection probe for the specific application of radioguided surgery have been ^125^I, ^111^In, ^99m^Tc, and, most recently, ^18^F.

**Table 3:** Physical properties of radionuclides that have been utilized with the gamma detection probe in radioguided surgery

Radionuclides	Physical half-life	Principle gamma photon radiation emission(s)	Emission probability per decay (percent photon yield)
Cobalt-57 (^57^Co)	271.8 days	14, 122, 136 keV	9.2, 85.5, 10.7%
Fluorine-18 (^18^F)	110 minutes	511 keV*	19.3%
Galium-67 (^67^Ga)	78.3 hours (3.26 days)	91, 93, 184, 209, 300, 393 keV	3.0, 37.8, 20.1, 2.4, 16.8, 4.7%
Indium-111 (^111^In)	67.4 hours (2.81 days)	171, 247 keV	90.7, 94.1%
Iodine-123 (^123^I)	13.2 hours	159, 529 keV	83.4, 1.3%
Iodine-124 (^124^I)	100.3 hours (4.18 days)	511 keV*	not easily characterized
Iodine-125 (^125^I)	1443.4 hours (60.14 days)	35 keV	6.7%
Iodine-131 (^131^I)	193.0 hours (8.04 days)	80, 284, 364, 637, 642, 723 keV	2.6, 6.1, 81.2, 7.3, 0.2, 1.8%
Technetium-99m (^99m^Tc)	6.04 hours	140, 142 keV	88.5, 0.023%
Thallium-201 (^201^Tl)	73.0 hours (3.04 days)	71, 135, 167 keV	47.0, 2.7, 10.0%

* The 511 keV gamma photons are generated from positron-electron annihilation.

### Radionuclides of iodine

Four radionuclides of iodine have been utilized in radioguided surgery, including ^123^I, ^124^I, ^125^I, and ^131^I [26,28,29]. In this regard, various radiopharmaceutical agents have been developed using radionuclides of iodine in conjunction with monoclonal antibody carriers as well as receptor-specific carriers and tissue-specific carriers.

By far, ^125^I has been utilized most frequently in the past in the form of a radiolabeled conjugate with various monoclonal antibodies for gamma probe detection of tumor in radioguided surgery [26,28,30]. ^125^I has a relatively long physical half-life of approximately 60 days and possesses an extremely low gamma photon emission energy of 35 keV. Generally speaking, ^125^I is not suitable for diagnostic nuclear medicine imaging due to its low gamma photon emission energy, which results in weak tissue penetration and high soft tissue attenuation, and a resultant poor image quality. Instead, diagnostic gamma camera imaging is more ideally suited for radionuclides with gamma photon emission energies in the 100 keV to 200 keV range. However, the low gamma photon emission energy and high soft tissue attenuation of ^125^I is highly advantageous in gamma probe detection of tumor in radioguided surgery, since the principle of gamma probe detection generally relies on close approximation of the gamma detection probe to the source of the radioactivity for the facilitation of accurate tumor detection. Additionally, the long physical half-life of ^125^I has been shown to be advantageous for gamma probe detection of tumor in radioguided surgery involving whole monoclonal antibodies due to the prolonged time of approximately 14 to 21 days that its takes for such ^125^I-radiolabeled whole monoclonal antibody conjugates to reach optimal pharmacokinetics and to accomplish maximal tumor localization with maximum background washout.

^131^I has a physical half-life of approximately 8 days and was the first radionuclide used in the radiolabeling of monoclonal antibodies [26,28,30]. The principle gamma photon emission energy of ^131^I that is utilized in nuclear medicine is that of the 364 keV gamma photon. The highly energetic nature of these 364 keV gamma photons increase background counts secondary to scatter and resultantly complicates the tumor detection efficiency of gamma probe detection of tumor during radioguided surgery. Likewise, these highly energetic 364 keV gamma photons require high-energy collimation and are generally less well-detected by diagnostic gamma camera imaging secondary to the limited stopping ability of the crystal element within the diagnostic gamma camera imaging device. The beta particulate emissions of ^131^I contribute significantly to the absorbed dose of radiation to the patient, thus limiting the amount of ^131^I dose that can be administered to the patient. The utilization of ^131^I has principally been limited to that of therapeutic applications for obliteration of thyroid tissue and for radioguided surgery for guiding the resection of recurrent thyroid cancer after diagnostic imaging.

^123^I has a physical half-life of approximately 13 hours, has a principle gamma photon emission energy of 159 keV, and has a relative absence of beta particulate emissions [26,28,30]. These features allow one to administer relatively larger doses of ^123^I dose to patients relative to other radionuclides of iodine. Likewise, these features make ^123^I relatively ideal for detection by diagnostic gamma camera imaging with a number of different carrier agents, including metaiodobenzylguanidine (MIBG), since diagnostic gamma camera imaging is most ideally suited for radionuclides with gamma photon emission energies in the 100 keV to 200 keV range. Additionally, ^123^I-labeled MIBG has been successfully used in gamma probe detection during radioguided surgery. However, the short physical half-life of approximately 13 hours for ^123^I makes it somewhat unsuitable for radioiodination of ^123^I to whole monoclonal antibodies for use in gamma probe detection during radioguided surgery, since such ^123^I-labeled whole monoclonal antibody conjugates may take many days to reach optimal pharmacokinetics and to accomplish maximal tumor localization with accompanying maximum background washout. However, ^123^I labeled monoclonal antibody fragments have been used in diagnostic gamma camera imaging.

^124^I has a physical half-life of approximately 4 days [31-33]. The emission spectrum and decay schema for ^124^I is very complex and is beyond the scope of this review to fully characterize. Only about 23% of disintegrations from ^124^I result in positron emissions, and these are generally of relatively high energy [32]. There are also numerous high-energy gamma photon emissions that occur, some of which occur in cascade with the positron emissions [32]. ^124^I has been used primarily in diagnostic positron emission tomography (PET) imaging [32,34], but is currently under investigation in radioguided surgery with gamma detection probes and PET probes (measuring both high-energy gamma photon emissions and beta emissions) [29].

### ^111^In

^111^In has a physical half life of approximately 2.8 days, has two principle gamma photon emissions with energies of 171 keV and 247 keV, and has a relative absence of beta particulate emissions [26,28]. These features make ^111^In a relatively ideal radionuclide for detection by diagnostic gamma camera imaging in conjunction with a number of different carrier agents. However, the propensity of ^111^In-containing radiopharmaceuticals, such as ^111^In-labeled monoclonal antibodies, to accumulate within the reticuloendothelial system (such as the liver, spleen, and bone marrow) result in relatively high background counts and can limit its potential usefulness for gamma probe detection of tumor during radioguided surgery.

### ^99m^Tc

^99m^Tc has a physical half-life of approximately 6 hours, has a principle gamma photon emission energy of 140 keV, and has a relative absence of beta particulate emissions [26,28]. ^99m^Tc remains the leading radionuclide imaging agent used in diagnostic nuclear medicine secondary to several desirable characteristics, including: (1) the principle gamma photon emission energy of 140 keV that is ideal both for detection by diagnostic gamma camera imaging and for gamma probe detection of tumor and identification of SLNs during radioguided surgery; (2) a low patient-absorbed radiation dose; (3) a low cost per patient dose; and (4) widespread commercial availability.

### ^18^F

A radionuclide to more recently gain interest for radioguided surgery has been ^18^F [35-54]. ^18^F has a relatively short physical half-life of approximately 110 minutes [55,56]. The radioactive decay of ^18^F is predominantly (97%) by positron (positively charged electron) emission. The maximum positron radiation emission energy of ^18^F is 635 keV, giving ^18^F a relatively low positron radiation emission energy. As a result, the positron emitted from the nucleus of this proton-rich/neutron-deficient radionuclide can travel only a short distance (approximately 2 millimeters) within the biological tissue before it interacts (collides) with a negatively charged electron. It is the interaction (collision) of the emitted positron from the ^18^F nucleus with a negatively charged electron within the biological tissue and the resultant positron-electron annihilation within the biological tissue that then generates two high-energy 511 keV gamma photons. Therefore, the resultant detection of ^18^F during radioguided surgery can occur by one of two mechanisms: (1) a direct mechanism of detection of positrons (beta particulate emissions) by a positron detection probe or (2) an indirect mechanism of detection of high-energy 511 keV gamma photons arising from positron-electron annihilation by a gamma detection probe. These highly energetic 511 keV gamma photons, that are the basis of the indirect mechanism of detection by a gamma detection probe, can result in relatively high background counts and can potentially complicate tumor detection efficiency of the gamma detection probe during radioguided surgery.

## Radiopharmaceutical agents utilized in radioguided surgery

Radioguided surgery using gamma detection probe technology has undergone an ever-changing evolution with regards to which radiopharmaceutical agents have been most frequently employed [49]. In the early years of gamma probe detection in radioguided surgery, the radiopharmaceutical agents most frequently utilized were radionuclides of iodine that were labeled to various monoclonal antibodies. More recently, with the advent of radioguided SLN biopsy technology and its application to numerous surgically managed malignancies, the use of ^99m^Tc-labeled radiopharmaceutical agents with the gamma detection probe in radioguided surgery has increased dramatically, and, at the present time, accounts for the vast majority of the radioguided surgical procedures performed. However, future directions for the gamma probe in radioguided surgery are currently being evaluated and the use of ^18^F-fluorodeoxyglucose (^18^F-FDG) as a radiopharmaceutical agent for gamma probe detection of tumor in radioguided surgery holds exciting promise [46,49].

### Monoclonal antibodies and their tumor-associated antigens

The specific application of monoclonal antibodies to radioguided surgery has been the basis for, and has represented the most important component to, the development of the radioimmunoguided surgery (RIGS) system [30,57]. This system was pioneered at The Ohio State University in the early 1980s by the collaboration of a surgical oncologist, Dr. Edward W. Martin, Jr., and a professor emeritus of electrical engineering, Dr. Marlin O. Thurston [58,59].

The production of a monoclonal antibody is the result of a technique called hybridoma fusion technology [60]. Most simply stated, a B-cell lymphocyte (which recognizes a single particular antigen and subsequently produces a single antibody targeting that specific antigen) and a myeloma cell are fused together to create a hybridoma cell. This immortalized hybridoma cell has the ability to survive and replicate outside of the animal. Such a hybridoma cell is able to replicate and be maintained in cell culture and will produce large amounts of a single antibody, which is referred to as a monoclonal antibody.

Monoclonal antibodies used in RIGS can be targeted against antigens expressed on the surface of tumor cells or targeted against antigens expressed within the extracellular environment around tumor cells [30,49,57]. When radiolabeled with various radionuclides, such resulting radiolabeled monoclonal antibody conjugates can potentially be utilized in both diagnostic gamma camera imaging and gamma probe detection of tumors, as well as in cancer therapeutics. In this regard, both whole monoclonal antibodies and monoclonal antibody fragments have been investigated.

The most advantageous features of an ideal monoclonal antibody are: (1) high affinity for its antigen (i.e., the initial ability to bind to the antigen); (2) high avidity for its antigen (i.e., the ability of the antibody to remain bound over an extended period of time); (3) rapid penetration into the tumor tissue; (4) rapid clearance from the bloodstream; (5) minimal accumulation within normal tissues; and (6) the absence of a human antimouse antibody (HAMA) response [8,17,30,58,59,61].

Nevertheless, the production of radiolabeled monoclonal antibody is not necessarily a simple endeavor [26,30]. The conjugation of a radionuclide to a monoclonal antibody may potentially change the specific binding properties of the monoclonal antibody. In such an instance in which the specific binding properties of the monoclonal antibody are significantly altered, the resultant radiolabeled monoclonal antibody may be left with significantly reduced affinity and/or avidity for the intended target antigen that ultimately renders the resultant radiolabeled monoclonal antibody clinical ineffectual.

The particular form of the monoclonal antibody (i.e., whether it is a whole monoclonal antibody or a fragment of a monoclonal antibody) can influence its ability to localize tumor [30]. Monoclonal antibody fragments have smaller molecular weight, have more rapid penetration into tumors, and have more rapid clearance rate from the bloodstream. As a result, the use of radiolabeled monoclonal antibody fragments can result in lower normal tissue background activity and lead to increased tumor to background ratio and improved tumor detections. However, monoclonal antibody fragments tend to accumulate more within the kidneys and, as a result, they may not be useful in the evaluation of the tumors within or around the area of the kidneys or the bladder.

Numerous radiolabeled monoclonal antibodies have been clinically investigated for radioimmunodetection and in RIGS [30,49]. The most intensely investigated and clinically evaluated monoclonal antibodies have been those directed against tumor-associated glycoprotein-72 (TAG-72), carcinoembryonic antigen (CEA), and tumor-associated antigen 17-1A. Several generations of anti-TAG-72 monoclonal antibodies have been developed, including two murine-derived anti-TAG-72 monoclonal antibodies (B72.3, native murine CC49) and one humanized anti-TAG-72 monoclonal antibody (HuCC49).

TAG-72 is a tumor-associated glycoprotein with a molecular weight of greater than 10 million Daltons [62,63]. TAG-72 contains approximately 80% carbohydrates, has mucin-like biochemical and biophysical properties similar to colonic, small intestine, and gastric mucins, and is thought to be secreted by epithelial tissues [62,63]. Numerous epithelial-derived cancers, including colorectal, breast, gastric, pancreatic, ovarian, and non-small cell lung cancers overexpress TAG-72 [62,64]. TAG-72 is predominantly located within mucin pools of the extracellular environment around the tumor cells and is not specifically expressed on the tumor cell surface. Of particular importance, TAG-72 has been shown to be associated with over 90% of the colorectal, gastric, and ovarian carcinomas and in approximately 70% of breast carcinomas [65-68]. Finally, while it is rarely expressed in normal human adult tissues or in benign disease processes, TAG-72 is also expressed in some normal human fetal tissues, including normal fetal intestine [69].

B72.3 was the first-generation murine anti-TAG-72 monoclonal antibody that was developed and was interestingly first derived from reaction with human mammary tumor cells [70]. B72.3 was shown to be reactive with a variety of human carcinomas, including colorectal (94%), breast (84% of invasive ductal), ovarian (100% of common epithelial), as well as the majority of gastric, pancreatic, endometrial, and lung adenocarcinomas [30,65,67-69,71]. In contrast, B72.3 was shown to have only a very weak or a nonreactivity status to a variety of normal adult human tissues [30]. The only exception to this rule has been demonstrated for normal postovulatory (secretory phase) endometrium which was shown to be reactive to B72.3, in contrast to normal preovulatory (proliferative phase) endometrium which was nonreactive [30,71].

Native murine CC49 was the second-generation murine anti-TAG-72 monoclonal antibody that was developed [30,63,72,73]. Native murine CC49 was found to have only minimal reactivity to a variety of normal human tissues, recognized a different epitope on the TAG-72 as compared to B72.3, and exhibited higher reactivity than B72.3 to a variety of human carcinomas, including colorectal, breast, ovarian, and lung carcinomas [30,72,73]. From a clinical perspective and as will later be discussed in the clinical application section, native murine CC49 was also superior to B72.3 in tumor detection in RIGS for colorectal carcinoma [74,75].

It is well characterized that a majority of patients will develop some degree of a HAMA response to the administration of murine monoclonal antibodies [30,64,76-78]. Despite the fact that the HAMA response has been well characterized, its clinical impact on cancer patients, whether deleterious or beneficial, remains very unclear [79,80]. Nevertheless, in order to attempt to eliminate this antiimmunoglobulin response, a third-generation humanized anti-TAG-72 monoclonal antibody (HuCC49) was genetically engineered [81]. HuCC49 demonstrated equivalent tumor-targeting for human colon carcinoma xenografts but a tradeoff of slightly less relative affinity to TAG-72 as compared to native murine CC49 and chimeric CC49 [81]. However, HuCC49 was shown to not produce a HAMA response [82]. Further refinements were made in HuCC49 by the development of a higher affinity HuCC49 possessing a CH2 domain deletion (i.e., HuCC49ΔC_H_2) [83]. HuCC49ΔC_H_2 demonstrated a more rapid blood clearance, a higher affinity constant (5.1 × 10^-9 ^versus 2.1 × 10^-9^), and significantly lower percent of the injected dose in normal tissues compared to intact HuCC49 [83], thus indicating the potential utility of the HuCC49ΔC_H_2 monoclonal antibody for diagnostic and therapeutic clinical applications. Furthermore, population pharmacokinetic modeling studies have demonstrated that HuCC49ΔC_H_2 had more rapid clearance (65% increase) from bloodstream and a resultant shorter "residence time" (24% shorter) than that of native murine CC49 [84].

Carcinoembryonic antigen (CEA) represents another well-studied and potentially useful target antigen for which radiolabeled monoclonal antibodies have been developed and investigated for RIGS [30]. CEA is a tumor-associated glycoprotein with a molecular weight of approximately 200,000 Daltons [85,86]. It is highly expressed on the cell surface of both embryonic colonic mucosa as well as a wide range of human adenocarcinomas, including colorectal, gastric, pancreatic, breast, ovarian, endometrial, and lung [30,85-87]. Specific to colorectal adenocarcinomas, it has been previously reported that anywhere from 66% to 100% express CEA [30].

Numerous murine monoclonal antibodies have been developed to target CEA [30,85,88-93]. Those most well studied have included COL-1, A_5_B_7_, IMMU-4, and CL58. COL-1 monoclonal antibody was first derived from reaction with LS-174T human colon carcinoma xenograft in athymic mice, has a very high affinity to CEA, and has been shown to have a high reactivity to significant number of colon, breast, and lung carcinomas [30,85,88,89]. Likewise, A_5_B_7_, IMMU-4, and CL58 represent three additional anti-CEA murine monoclonal antibodies that have shown clinical relevance by possessing a high reactivity to CEA-producing malignancies [30,88,90-93].

Lastly, 17-1A (also called EpCAM) is a tumor-associated glycoprotein with a molecular weight in the range of approximately 30, 000 to 40,000 Daltons [94-96] which is thought to represent a cell-cell adhesion molecule. It was first characterized on a human colorectal adenocarcinoma cell line SW1083 [97]. It is broadly distributed in normal epithelial tissues and in various carcinomas, including colorectal, gastric, and breast [94,95,98].

Murine monoclonal antibodies against the tumor-associated antigen 17-1A were originally developed in the hybridoma SW1083-17-1A [57,99,100]. The localization and clearance properties of the 17-1A murine monoclonal whole antibody and its monoclonal antibody fragment were previously evaluated in a mice xenograft model by Martin et al [101], demonstrating high tumor-to-normal tissue ratios with highest tumor-to-normal tissue ratios seen at 72 hours and 24 hours, respectively, for the 17-1A murine monoclonal whole antibody and monoclonal antibody fragment [57,101].

The most common challenges facing the utility of monoclonal antibodies in radioimmunodetection relate to the activity ratio between tumor and normal surrounding tissues and the time interval between the initial administration of the radiopharmaceutical agent and performance of diagnostic gamma camera imaging or radioguided surgical detection. In an attempt to increase the activity ratio between tumor and normal surrounding tissues and to decrease the time interval between the initial administration of the radiopharmaceutical agent and performance of diagnostic gamma camera imaging or radioguided surgical detection, pretargeting strategies for monoclonal antibodies and radionuclides have been investigated [102]. Most such pretargeting strategies utilize the principle of the avidin-biotin binding system. This avidin-biotin pretargeting strategy allows for the complete temporal separation of the systemic administration of the monoclonal antibody from that of the systemic administration of the radionuclide. The monoclonal antibody is labeled with biotin and the radionuclide is labeled with avidin. This will ultimately result in a reduction of nonspecific binding. The biotin-labeled monoclonal antibody is first administered, allowing binding of the biotin-labeled monoclonal antibody to the tumor and allowing the nonspecific uptake of the biotin-labeled monoclonal antibody to be cleared. The avidin-labeled radionuclide is then administered and resultantly localizes in the tumor secondary to the high affinity and specificity of the avidin-labeled radionuclide for the biotin-labeled monoclonal antibody. More recently, an additional pretargeting strategy utilizing a bispecific antibody and radiolabeled bivalent hapten system has been investigated that bind cooperatively to target cells [103].

### Radioactive iodine-labeled radiopharmaceutical agents

The vast majority of radioactive iodine-labeled radiopharmaceutical agents that have been utilized with the gamma detection probe for tumor detection in radioguided surgery have been those radionuclides of iodine that have been labeled to various monoclonal antibodies [30]. The predominant iodine radionuclide that has been labeled to various monoclonal antibodies and utilized with the gamma detection probe for tumor detection in radioguided surgery has been ^125^I, and to a much lesser degree ^131^I. The radiolabeling of ^123^I to monoclonal antibodies has not been proven useful for tumor detection in radioguided surgery for the reasons previously discussed. Both ^131^I and ^123^I are used with MIBG, a molecule similar to norepinephrine, for identification of neuroendocrine tumors.

### ^99m^Tc-labeled radiopharmaceutical agents

Numerous ^99m^Tc-labeled radiopharmaceutical agents have been formulated for use in diagnostic nuclear medicine by radiolabeling the radionuclide ^99m^Tc to various compounds [26,104]. The list of compounds that have been radiolabeled with ^99m^Tc for diagnostic nuclear medicine use is extensive and includes, in alphabetical order, antimony trisulfide colloid, bicisate dihydrochloride, colloidal human albumin (i.e., nanocolloid), colloidal rhenium sulfide, dextran, diethylenetriaminepentaacetic acid (DTPA)-mannosyl-dextran, disofenin, hydroxyl-ethyl starch, exametazime, gluceptate, glucoheptonate, hexakis-2-methoxy-isobutyl-isonitrile (methoxyisobutylisonitrile, MIBI, or sestamibi), hydroxymethylene diphosphonate (HMDP or oxidronate), hydroxyethylene diphosphonate (HDP), lidofenin, mebrofenin, mertiatide (mercaptoacetylglyclyglyclyglycine), methylene diphosphonate (MDP or medronate), pentetate (diethylenetriaminepentaacetic acid), sodium pertechnetate, sodium phytate (D-myo-inositol 1,2,3,4,5,6-hexakisphosphate dodecasodium), sodium pyrophosphate, stannous phytate, succimer, sulfur colloid, teboroxime, tetrofosmin, and tin colloid. The primary ^99m^Tc-labeled radiopharmaceutical agents that have been used for radioguided SLN biopsy include ^99m^Tc sulfur colloid, ^99m^Tc colloidal human albumin, and ^99m^Tc antimony trisulfide colloid. The primary ^99m^Tc-labeled radiopharmaceutical agents that have been used for tumor detection during radioguided surgery include ^99m^Tc MIBI (sestamibi), ^99m^Tc diphosphonates, and ^99m^Tc sodium pertechnetate. The application of ^99m^Tc-labeled monoclonal antibody fragments, such as^99m^Tc-labeled arcitumomab (IMMU-4 murine monoclonal antibody fragments against CEA) and ^99m^Tc-nofetumomab merpentan (monoclonal antibody fragment of the pancarcinoma murine antibody NR-LU-10) have been used in nuclear medicine imaging but have only been very limitedly investigated for tumor detection during radioguided surgery [105-107].

### ^111^In-labeled radiopharmaceutical agents

Several ^111^In-labeled radiopharmaceutical agents have been formulated for use in diagnostic nuclear medicine by radiolabeling the ^111^In with various compounds [26,30,108,109]. This includes the ^111^In-labeled somatostatin analogue, ^111^In-diethylenetriaminepentaacetic acid-D-phenylalanine^1^-octreotide (^111^In-DTPA-D-Phe^1^-octreotide or ^111^In-pentetreotide), as well as various ^111^In-labeled monoclonal antibodies. ^111^In-(DTPA)-D-Phe^1^-octreotide binds to somatostatin receptors, predominantly of somatostatin receptor subtype sst2 and sst5, and have been useful for diagnostic nuclear medicine imaging of neuroendocrine tumors and of non-neuroendocrine tumors which express somatostatin receptors [26,108]. Likewise, ^111^In-labeled monoclonal antibodies have been investigated in colorectal cancer [30,109]. However, ^111^In-labeled monoclonal antibodies have been of somewhat limited usefulness secondary to the previously discussed nonspecific accumulation of ^111^In within reticuloendothelial organs, such as within the liver and spleen. This resultant nonspecific binding generally interferes with the detection of tumor within or around the liver and spleen during radioguided surgery and is therefore undesirable [26,30].

### ^18^F-fluorodeoxyglucose (^18^F-FDG)

Malignant tumors have long been known to have an accelerated rate of glucose metabolism and have an increased rate of glucose transport and glucose utilization [110-112]. The mechanism of ^18^F-FDG within malignant cells is well described in the literature [113-115]. ^18^F-FDG is an ^18^F-labeled nonphysiologic analog of glucose. ^18^F-FDG within the bloodstream is transported into cells (both malignant cells and normal cells) by a facilitated diffusion mechanism involving specific glucose transporters (i.e., GLUT transporters). Once within the cell, ^18^F-FDG is phosphorylated to ^18^F-FDG-6-phosphate by the enzyme hexokinase. Unlike ^18^F-FDG, ^18^F-FDG-6-phosphate can not be readily transported across the cellular membrane of either malignant cells or normal cells. The enzyme glucose-6-phosphatase is responsible for dephosphorylating ^18^F-FDG-6-phosphate to ^18^F-FDG. Because the enzyme glucose-6-phosphatase is present in relatively low amounts within malignant cells and within normal cells, ^18^F-FDG-6-phosphate cannot be readily dephosphorylated back to ^18^F-FDG once ^18^F-FDG has been phosphorylated within the intracellular environment. Therefore, once ^18^F-FDG is transported into the malignant cell or the normal cell via the GLUT transporters and is subsequently phosphorylated, the resultant ^18^F-FDG-6-phosphate is essentially trapped within the cell. Additionally, ^18^F-FDG-6-phosphate cannot be utilized in the metabolic steps of glycolysis, and, this further lends to the accumulation of ^18^F-FDG-6-phosphate within the cell. This entire process is thought to occur more readily in malignant cells than in normal cells due to the overexpression of the glucose transporters GLUT 1 and GLUT 3 by malignant cells and due to higher levels of hexokinase within malignant cells. The overall result of this entire process of an accelerated rate of glucose metabolism and an increased rate of glucose transport and glucose utilization by malignant cells is that of a relatively greater accumulation of ^18^F-FDG-6-phosphate within malignant cells as compared to normal cells. Even more simply stated, malignant cells are much more efficient at accumulating glucose molecules within their intracellular environment than are normal cells. This elegantly elucidated process represents the overall basis for the clinical application of ^18^F-FDG for the detection of tumor by both diagnostic PET imaging and gamma detection probe technology.

However, limitations do exist in regards to the utilization of ^18^F-FDG in both diagnostic PET imaging and gamma detection probe technology. These limitations are: (1) the accumulation of ^18^F-FDG within certain normal tissues with an elevated rate of glucose metabolism (most striking in the brain and heart, and to a lesser degree in the mucosa and smooth muscle of the stomach, small intestine and colon, as well as in thyroid, liver, spleen, and skeletal muscle); (2) the accumulation of ^18^F-FDG within in inflammatory/granulomatous processes and infectious processes; (3) the excretion and accumulation of ^18^F-FDG within the urinary tract (kidneys, ureters, and bladder); and (4) the impaired uptake of ^18^F-FDG in patients with elevated blood glucose levels and with impaired glucose metabolism [113,116,117].

## Occupational radiation exposure from radiopharmaceutical agents utilized during radioguided surgery

The assessment of occupational radiation exposure to surgical personnel involved in radioguided surgical procedures is important to maintaining a safe work environment for such personnel. This has been evaluated to varying degrees for ^125^I, ^111^In, ^99m^Tc, and, ^18^F. The United States Nuclear Regulatory Commission (USNRC) has set the annual occupational exposure limit for adults as a total effective dose equivalent of 50,000 μSv [118]. The International Commission on Radiological Protection (ICRP) has set the annual occupational exposure limit for adults as a total effective dose equivalent of 20,000 μSv per year, averaged over a five year period (100,000 μSv in five years), with further provision that the total effective dose equivalent should not exceed 50,000 μSv in any single year [119,120].

For ^125^I-labeled monoclonal antibodies, the radiation exposure to the surgeon has been previously assessed during RIGS [78,121]. For a mean dosage of 2 mCi (74 MBq) of ^125^I that was radiolabeled to 1 mg of B72.3 monoclonal antibody and was injected at a mean of 23.4 days prior to surgery, the mean dose equivalent for the surgeon was determined to be only 0.2 μSv per hour of exposure and was not significantly different from that of the environmental (operating room) background.

For ^111^In radiopharmaceuticals, no data on radiation exposure to surgical personnel is currently available. However, in this regard, limited data from one study reported that staff members and technologists involved in treating patients with somatostatin receptor positive tumors with a therapeutic dosage (216 mCi or 8000 MBq) of an ^111^In-labeled somatostatin analogue received a mean whole body dose equivalent of only 45 μSv per case [122].

Radiation exposure to surgical personnel members from ^99m^Tc-labeled radiopharmaceutical agents used for radioguided SLN biopsy has been well-documented [123-127]. There was significant variability in the whole body dose equivalent incurred by the surgeon and this value was highly dependent upon the injection dose of the ^99m^Tc-labeled radiopharmaceutical agent and the total duration of time from the injection to the start of the radioguided SLN biopsy procedure. On one hand, Waddington et al reported mean whole body dose equivalent of only 0.34 μSv per case when 0.27 mCi (10 MBq) to 0.41 mCi (15 MBq) of ^99m^Tc colloidal albumin was injected 24 hours prior to the start of the radioguided SLN biopsy procedure [124]. On the other hand, Stratmann et al reported a mean whole body dose equivalent of 13.3 μSv per case when 0.7 mCi (26 MBq) to 1.1 mCi (41 MBq) of ^99m^Tc sulfur colloid was injected 90 to 180 minutes prior to the start of the radioguided SLN biopsy procedure [123]. Nevertheless, the data supports the fact that the whole body dose equivalent incurred by a surgeon during any given radioguided SLN biopsy procedure using a ^99m^Tc-labeled radiopharmaceutical agent is extremely low.

Radiation exposure to surgical personnel members from ^99m^Tc-MIBI used for radioguided brain tumorectomy has been previously investigated [128]. The dosage of ^99m^Tc-MIBI that was intravenously administered on the day of surgery was not specified. The mean exposure time was 6.1 hours for all surgical personnel members. The mean whole body dose equivalent per case was 27.9, 25.8, and 14.9 μSv, respectively, for the surgeon, nurse, and anesthetist. More recently, Bekiş et al [129] evaluated radiation exposure to surgical staff from ^99m^Tc-MIBI used in two cases of radioguided parathyroidectomy. When 20 mCi (740 MBq) of Tc-99m MIBI was intravenously injected 3 hours prior to the operation and with a mean operative time of 83 minutes, the least and highest exposure of the surgical staff were calculated as 1.01 and 3.63 μSv for the anesthetist, 8.78 and 11.00 μSv for the senior surgeon, 7.60 and 10.00 μSv for the first assistant surgeon, 6.75 and 8.20 μSv for the second assistant surgeon, and 3.64 and 8.00 μSv for the nurse.

The issue of radiation exposure to intraoperative and perioperative personnel involved in radioguided surgery cases utilizing ^18^F-FDG has become a topic of recent interest. However, data on this topic is currently very limited [42,47,50,51,130-132]. In a recent comprehensive evaluation, our group at The Ohio State University has determined that after a mean dosage of 18.9 mCi (699 MBq) of ^18^F-FDG injected at a mean of 142 minutes prior to surgery that the mean deep dose equivalent per case was 164, 119, 92, 63, 54, and 48 μSv, respectively, for the surgeon, anesthetist, scrub technologist, postoperative nurse, circulating nurse, and preoperative nurse [132]. This data clearly illustrates that the absorbed radiation dose received by both intraoperative and perioperative personnel involved in ^18^F-FDG radioguided surgery cases is relatively low per case and allows for all these personnel to participate in multiple such cases and still remain well below standards set for occupational exposure limits.

## Clinical applications (Table 2)

### Breast cancer

There are numerous reported applications of gamma detection probe technology during radioguided surgery for breast cancer. However, by far, the single most important and most widely utilized application of the gamma detection probe in breast cancer surgery has been for radioguided SLN biopsy.

#### Radioguided SLN biopsy

It is clearly evident that SLN biopsy has become widely accepted as a standard of care in the surgical staging of the axillary lymph nodes during breast cancer surgery [133,134]. The intraoperative use of the gamma detection probe for radioguided SLN biopsy in breast cancer was first described in 1993 by Krag et al [135] at The University of Vermont (Table 1). Since that time, over 3,300 articles have been published which have been sited in PUBMED under the search descriptors of "breast cancer" and "sentinel lymph node".

Worldwide, numerous ^99m^Tc-based agents have been utilized for radioguided SLN biopsy for breast cancer [133,136]. This includes ^99m^Tc sulfur colloid, ^99m^Tc antimony trisulfide colloid, ^99m^Tc colloidal human albumin (i.e., ^99m^Tc nanocolloid), ^99m^Tc tin colloid, ^99m^Tc labeled dextran, ^99m^Tc hydroxyl-ethyl starch, and ^99m^Tc stannous phytate. However, within the United States, ^99m^Tc sulfur colloid represents the only registered and licensed radiocolloid for use SLN biopsy. The dosing of these radiocolloid agents varies considerably within the literature and have been reported as low as 0.1 mCi (3.7 MBq) [137] and as high as 10 mCi (370 MBq) [138]. Generally, in current practice, these radiocolloid agents are most commonly administered on the day of surgery in a dosing range from 0.4 mCi (14.8 MBq) to 1 mCi (37 MBq) [133]. These radiocolloid agents are generally administered one to six hours prior to the planned breast cancer surgery, although prior-day injection of radiocolloid has been shown to be technically feasible [137,139,140].

The administration of a radiocolloid agent for SLN biopsy in breast cancer surgery has been described by numerous injection routes, including intraparenchymal (peritumoral), intradermal, subdermal, subareolar, and intratumoral [133]. Within the United States, the three predominant injection routes have been intraparenchymal, intradermal, and subareolar. Until recently, all data comparing these injection routes has been retrospective in nature. However, recently, the first prospective randomized clinical trial comparing the intraparenchymal, intradermal, and subareolar injection routes was conducted among 400 breast cancers at The Ohio State University and utilizing a dose of approximately 0.4 mCi (14.8 MBq) of ^99m^Tc sulfur colloid [141]. This recently reported study demonstrated superior intraoperative gamma probe localization of ^99m^Tc sulfur colloid within the axillary lymph nodes for the intradermal route (100%), as compared to the subareolar route (95%) and intraparenchymal route (90%) [141]. Additionally, this study demonstrated that intradermally injected ^99m^Tc sulfur colloid resulted in the greatest maximum sustainable ex vivo counts within the hottest axillary SLN and the shortest intraoperative time to harvest the axillary SLN.

The determination of an adequate intraoperative assessment of the axilla with the gamma detection probe during breast cancer surgery is clearly related to the number of SLN candidates harvested and meticulous intraoperative attention to attempting to identify all potential sentinel lymph node candidates with counts of at least 10% of the counts of the hottest SLN. The most compelling argument for this is based on the results of two reports published by the University of Louisville Breast Cancer Sentinel Lymph Node Multiinstitutional Study Group [142,143] which evaluated patients undergoing SLN biopsy and a concomitant confirmatory axillary lymph node dissection. In 2001, Wong et al [142] demonstrated a false negative rate of 14.3% in patients who had a single SLN was harvested as compared to 4.3% in patients who had two or more SLNs were harvested (P = 0.0004). Identically, in 2005, Martin et al [143] reproduced those same results, demonstrating a false negative rate of 13.7% in patients who had a single SLN harvested as compared to 5.4% in patients who had two or more SLNs harvested (P < 0.0001). A re-emphasis of this concept has been brought back to our attention in several more recently published reports [144-147]. In 2007, Povoski et al [144] demonstrated that although 83% of cases had the first positive SLN identified as the hottest SLN, 17% of cases had the first positive SLN identified as the second, third, fourth, or fifth hottest SLN. Additionally, in this report, the SLN was positive in a significantly greater frequency of cases in which two or more SLNs were identified as compared to when only a single SLN was identified (34% versus 18%, P = 0.003). Likewise, Woznick et al [145] reported in 2006 that although 77% of cases had the first positive SLN identified as the first removed SLN, 23% of cases had the first positive SLN identified as the second, third, fourth, fifth, or sixth SLN removed. Similarly, Wada et al [146] reported in 2007 that although 76% of cases had the first positive SLN identified as the hottest SLN, 14% of cases had the first positive SLN node identified as the second and third hottest removed SLN, with an additional 3% having the positive SLN identified as a non-radioactive, blue-stained SLN, and an additional 7% having false-negative results. Finally, Krag et al [147], in the NSABP (National Surgical Adjuvant Breast and Bowel Project) B-32 randomized phase 3 trial, clearly demonstrated a significant association of the reduction in the observed false-negative rate as the total number of SLNs that were removed increased (i.e., with a 17.7%, 10.0%, 6.9%, 5.5%, and 1.0% false-negative rate when one, two, three, four, and five or more SLNs were removed, respectively). These six studies [142-147], when taken together, raise significant concern with the scenario in which only a single negative SLN candidate is intraoperatively identified with the gamma probe and emphasizes the importance of a meticulous intraoperative search for additional SLN candidates with counts of at least 10% of the counts of the hottest SLN.

#### RIGS

The application of RIGS technology to the breast has been previously investigated and reported in a very limited fashion [148-152]. In 1989, Nieroda et al [148,149] intraoperatively evaluated 14 patients with breast cancer with the gamma detection probe after injection 5 mCi (185 MBq) of ^125^I-labeled monoclonal antibody B72.3 at a time of six to 24 days prior to the surgical procedure. In 1996, and again identically re-reported in 1998, Percivale et al [150] and Badellino et al [151] intraoperatively evaluated 21 patients with locally advanced breast cancer with the gamma detection probe after injection of either 1.5 mCi (56 MBq) of ^125^I-labeled monoclonal antibody B72.3 or 1.5 mCi (56 MBq) of ^125^I-labeled fragments of the murine anti-CEA monoclonal antibody F023C5 at a mean time of 21.7 days or 10.3 days, respectively, prior to the surgical procedure. In 2001, Burak et al [152] intraoperatively evaluated 10 patients with breast cancer with the gamma detection probe after injection 2 mCi (74 MBq) of ^125^I-labeled NR-LU-10 antibody fragments at a time of two to seven days prior to the surgical procedure. In all three instances, specific binding to histologically confirmed sites of breast cancer was noted. However, the clinical impact of this technology in the context of these ^125^I-labeled-antibody complexes has not been more recently further investigated in the arena of breast cancer surgery.

#### Radioguided occult lesion localization (ROLL)

Radioguided occult lesion localization (ROLL), using gamma detection probe technology, is well described in the literature as an alternative to conventional wire localization for nonpalpable breast lesions, including breast cancer [153-181]. Most commonly, this ROLL technique involves an intratumoral injection (using preoperative same-day or prior-day mammographic or ultrasound guidance) of a ^99m^Tc-based agent (such as colloidal human serum albumin, nanocolloid, macroaggregate albumin, or dextran) in a dosing range from 0.05 mCi (1.8 MBq) to 4.0 mCi (148 MBq) [153-155,157,158,160-171,173-181]. As a result of the intratumoral injection of a 99mTc-based agent, this ROLL technique allows for simultaneous performance of a radioguided SLN biopsy procedure. In the largest retrospective series to date, Monti et al [168], 959 patients with cytologically or histologically proven breast cancer underwent ROLL plus radioguided SLN biopsy, with successful breast lesion localization in 99.6% of cases and negative surgical margins obtained in 91.6% of cases. Additionally, a ROLL technique involving placement of a radioactive seed (consisting of a 4.5 mm by 0.8 mm titanium seed containing 0.125 mCi (4.6 MBq) to 0.29 mCi (10.7 MBq) of ^125^I) up to five days in advanced to the date of surgery [156,159] has been described. Finally, and most recently, ROLL techniques have been described utilizing an intravenous intraoperative injection of 20 mCi (740 MBq) of ^99m^Tc sestamibi [167] on the day of surgery and utilizing an intravenous injection of 6 mCi (222 MBq) of ^111^In pentetreotide [172] at approximately 24 hours prior to surgery. In a systematic review detailing the available data in the literature on the ROLL technique, it was concluded that the ROLL technique compared favorably to that of conventional wire localization for nonpalpable breast lesions [176]. A large-scale (over 300 patients), multicenter, prospective clinical trial in the Netherlands to evaluate the ROLL technique compared to conventional wire localization for nonpalpable breast cancers is currently being planned [182].

#### Radioguided intraoperative margins evaluation (RIME)

Radioguided intraoperative margins evaluation (RIME), using gamma detection probe technology, is limitedly described in the literature as a technique for not only guiding resection of the primary tumor but also for intraoperative assessment and determination of the adequacy of surgical margins [183,184]. The feasibility of this technique was first described utilizing a preoperative same-day intravenous injection of 0.172 mCi (6.4 MBq) of ^125^I-labeled Lanreotide (a radiolabeled somatostatin analog) [183]. More recently, feasibility of the RIME technique was described utilizing a preoperative same-day intravenous injection of 20 mCi (740 MBq) of ^99m^Tc sestamibi [184].

#### ^18^F-FDG-directed surgery

Very recently, the application of gamma detection probe technology in radioguided surgery after a preoperative same-day injection of ^18^F-FDG has been reported in a limited number of selected cases of breast cancer [43,45,47,51]. In this regard, and of most recent note, our own group at The Ohio State University has recently reported a combined approach of perioperative ^18^F-FDG PET/CT imaging (using a triad of preoperative PET/CT imaging, specimen PET/CT imaging, and postoperative PET/CT imaging) and intraoperative gamma detection probe technology, using a dose of approximately 14 to 19 mCi (518 to 703 MBq) ^18^F-FDG injected intravenously approximately 120 minutes prior to the anticipated time of surgery for two selected cases of breast cancer for guiding tumor localization and for verifying complete tumor resection [51].

### Cutaneous malignancies

#### Malignant melanoma

The single most important and most widely utilized application of gamma detection probe technology for the surgical management of malignant melanoma is radioguided SLN biopsy. More recently, the application of ^18^F-FDG-directed surgery to the management of appropriately selected cases of metastatic or recurrent malignant melanoma has received increasing interest within the surgical community.

##### Radioguided SLN biopsy

The application of SLN biopsy for the surgical staging of the regional lymph node basins during cutaneous malignant melanoma surgery has become widely accepted as a standard of care for cutaneous malignant melanoma of the trunk, extremities, and head and neck region [185]. To date, over 1,800 articles have been published which have been sited in PUBMED under the search descriptors of "melanoma" and "sentinel lymph node".

SLN biopsy in cutaneous malignant melanoma was first described in 1992 by Morton et al [186] using a technique of intradermally injected vital blue dye alone. In 1993, Uren et al [187] described a combined technique, consisting of preoperative lymphoscintigraphy (using ^99m^Tc antimony sulfide colloid) for identification of all sites of location that are simply marked on the skin surface with an indelible marker and of intraoperative intradermally injected vital blue dye (but without use of an intraoperative gamma detection probe), for SLN detection. Shortly thereafter in 1993, the intraoperative use of the gamma detection probe for radioguided SLN biopsy in cutaneous malignant melanoma was first reported by Alex et al [188] at The University of Vermont in ten patients using intradermally injected ^99m^Tc sulfur colloid (Table 1). Since that time, it has been clearly demonstrated that the use of radioguided SLN biopsy is superior to the vital blue dye alone technique for the surgical evaluation of at-risk nodal basins for cutaneous malignant melanoma [189-198]. The most compelling and well-respected international evidence for this comes from the Multicentric Selective Lymphadenectomy Trial Group results that were published in 1999 by Morton et al [193] on 570 melanoma patients. They demonstrated that the success of SLN identification was significantly improved by a combined radiocolloid and vital blue dye technique (n = 217) as compared to a vital blue dye alone technique (n = 352) (99.1% versus 95.2%, respectively; p = 0.014).

The primary ^99m^Tc-based agents utilized for radioguided SLN biopsy for cutaneous malignant melanoma are either ^99m^Tc sulfur colloid or ^99m^Tc colloidal human albumin (i.e., ^99m^Tc nanocolloid) [199-206]. Generally, these radiocolloid agents are most commonly administered one to six hours prior to the planned surgical procedure in a dosing range from 0.4 mCi (14.8 MBq) to 1 mCi (37 MBq). Radiocolloid is exclusively injected intradermally and is administered in one to four sites around the intact primary lesion or around the resultant excisional biopsy scar.

It is clearly evident that preoperative lymphoscintigraphy plays as important of a role as does intraoperative radioguided SLN biopsy for the successful identification of all potential SLN candidates during the surgical management of cutaneous malignant melanoma secondary to the potential for localization to multiple nodal basins [196,203,207-214] and for localization to in-transit (interval) SLNs [215-219]. For cutaneous malignant melanoma of the truncal region, preoperative lymphoscintigraphy localization to multiple nodal basins has been demonstrated in 17% to 32% of patient undergoing radioguided SLN biopsy [196,203,207-214]. Likewise, preoperative lymphoscintigraphy localization to in-transit (interval) SLNs has been demonstrated in 3% to 10% of patient undergoing radioguided SLN biopsy for cutaneous malignant melanoma of the extremities and of the head and neck region [215-219]. Therefore, unlike other malignancies in which radioguided SLN biopsy can be performed without the antecedent need of preoperative lymphoscintigraphy, the radioguided surgical approach to cutaneous malignant melanoma should include both preoperative lymphoscintigraphy and intraoperative utilization of the gamma detection probe.

The determination of an adequate intraoperative assessment of any given nodal basin with the gamma detection probe during radioguided SLN biopsy for cutaneous malignant melanoma is clearly related to the meticulous intraoperative attention to attempting to identify all potential SLN candidates with counts of at least 10% of the counts of the hottest SLN [220-225]. The first compelling argument for the 10% rule for malignant melanoma came from work published by McMasters et al [221] from the Sunbelt Melanoma Trial in which they found that within 13.1% of positive lymph node basins that the most radioactive SLN was negative for tumor while a less radioactive sentinel lymph node was positive for tumor. Additionally, in 50% of such cases, they found that these less radioactive positive SLNs contained 50% or less of the radioactive counts of the hottest non-positive SLN. Similarly, Carlson et al [222] in 2002, Jacob et al [223] in 2003, and Kroon et al [224] in 2007 found in 19.1%, 19.0%, and 11.0% of all positive SLN cases, respectively, that the hottest SLN was negative for tumor. As a result, all these reports have strongly advocated applying the 10% rule to reduce the risk of a missed lymph node metastasis during radioguided SLN biopsy for cutaneous malignant melanoma [220-225].

In addition to the vast body of data in the literature on the application of radioguided SLN biopsy for the surgical staging of conventional sites of cutaneous malignant melanoma (i.e., trunk, extremities, and head and neck region), various case reports and small series reports exist on the potential application of radioguided SLN biopsy for the surgical staging of less conventional sites of malignant melanoma, such as the ocular conjunctiva and periocular skin [226-234], the vulvar region [235-240], the vagina [236,238,240-242], and the anal canal [243-248]. For all these less conventional sites of malignant melanoma, the feasibility of radioguided SLN biopsy has been demonstrated. However, larger scale evaluation of this technology for these less conventional sites of malignant melanoma is obviously necessary in order to better assess its clinical relevance.

##### ^18^F-FDG-directed surgery

The ^18^F-FDG avidity of malignant melanoma has made the application of ^18^F-FDG PET imaging paramount to the accurate staging and restaging of malignant melanoma [185]. Likewise, ^18^F-FDG PET imaging and ^18^F-FDG PET/CT imaging have been shown to lead to a change in the clinical management of 17% to 39% of malignant melanoma patients, which is far greater than with other conventional imaging techniques alone [249-253]. While preoperative utilization of ^18^F-FDG PET imaging has become a standard of care for the clinical management of selected cases of malignant melanoma, the intraoperative use of ^18^F-FDG in combination with gamma detection probe technology has also been proposed and described in the clinical management of selected cases of metastatic malignant melanoma and has received increasing interest within the surgical community [38,40,41,43,47,52].

The intraoperative use of ^18^F-FDG in combination with gamma detection probe technology for selected cases of metastatic malignant melanoma was first reported in 2001 by Essner et al  [38]. Six patients were intravenously injected with 7 to 10 mCi (259 to 370 MBq) of ^18^F-FDG within three hours of the planned time of surgery. They reported that the tumor to background count ratio varied from 1.16:1 to 4.67:1, with eight of 13 melanoma metastases having a tumor-to-background count ratio of greater than 1.5:1. In 2005, Franc et al  [41] reported on 5 patients with recurrent malignant melanoma that were injected with 14.6 ± 3.2 mCi (540 ± 118 MBq) of ^18^F-FDG. They found that the use of gamma detection probe technology had a sensitivity of 89% and a specificity of 100% for the detection of tissues containing recurrent malignant melanoma. More recently in 2006, Gulec et al [43] reported on 26 patients with either recurrent or metastatic malignant melanoma that were intravenously injected with 7 to 10 mCi (259 to 370 MBq) of ^18^F-FDG within one to four hours of the planned time of surgery. They found that the tumor-to-background ratio ranged from 1.5:1 to 2.5:1, with a mean of 1.8:1, and metastatic lesions as small as 5 mm were detected.

The specific application of a combined approach of preoperative ^18^F-FDG PET/CT imaging and intraoperative gamma detection probe technology for recurrent malignant melanoma was first described in 2005 by Carrera et al [40] in a patient intravenously injected with 6.8 mCi (250 MBq) of ^18^F-FDG at approximately four hours prior to the planned time of surgery, illustrating the potential advantage to a combination of these technologies. Furthermore, the added benefit to such combination technologies for ^18^F-FDG-directed surgery was most recently illustrated by our own group at The Ohio State University in which a multimodality approach of perioperative ^18^F-FDG PET/CT imaging (preoperative patient imaging, specimen imaging, and postoperative patient imaging), intraoperative gamma detection probe technology, and intraoperative ultrasound were utilized for tumor localization and resection of all sites of hypermetabolic activity in a case of occult recurrent metastatic malignant melanoma [52]. In this case, the patient was intravenously injected with 12.8 mCi (474 MBq) of ^18^F-FDG at approximately 80 minutes prior to the planned time of surgery and this multimodality approach allowed for successful identification and resection of all three occult sites of recurrent metastatic malignant melanoma. Such refinements in multimodal ^18^F-FDG-directed surgery [40,52] may allow this approach to further positively impact upon the established survival advantage of complete surgical resection for malignant melanoma patients with limited metastatic disease [254].

#### Merkel cell carcinoma

The application of the gamma detection probe in radioguided surgery for Merkel cell carcinoma has been limited to radioguided SLN biopsy. In this regard, SLN biopsy for the surgical staging of the regional lymph node basins is generally accepted as a standard of care for previously untreated, clinical stage I Merkel cell carcinoma [255,256]. The utilization of radioguided SLN biopsy for Merkel cell carcinoma was first reported in 1997 by Messina et al [257]. Since that time, multiple reports have been published on the application of radioguided SLN biopsy in the surgical management of Merkel cell carcinoma [258-274].

The technique of radioguided SLN biopsy for Merkel cell carcinoma is very similar to that for malignant melanoma. The primary ^99m^Tc-based agents utilized for radioguided SLN biopsy for Merkel cell carcinoma are either ^99m^Tc sulfur colloid or and ^99m^Tc colloidal human albumin. These radiocolloid agents are generally administered on the morning of the planned surgical procedure in a dosing range from 0.3 mCi (11 MBq) to 4 mCi (150 MBq), are exclusively injected intradermally, and are administered in one to four sites around the intact primary lesion or around the resultant excisional biopsy scar [257-274].

The two largest radioguided SLN biopsy series for Merkel cell carcinoma reported to date were published by Allen et al in 2001 [264] and Maza et al in 2006 [272]. Allen et al [264] reported on 26 patients with clinical stage I Merkel cell carcinoma in which a SLN was detected in all patients using ^99m^Tc sulfur colloid and metastatic disease was found in five (19%) patients. Maza et al [272] reported on 23 patients with clinical stage I Merkel cell carcinoma in which a SLN was detected in all patients using ^99m^Tc colloidal human albumin and metastatic disease was found in eleven (48%) patients.

#### Other cutaneous malignancies

Radioguided SLN biopsy has been limitedly investigated in other cutaneous malignancies. It has been described for high risk squamous cell carcinoma of the facial region (face, ears, nose, and lips) [268,269,275-278] and of the trunk and extremities [268,269,279-283], for high risk basal cell carcinoma [284,285], for sweat gland carcinoma [269,286-288], and for cutaneous lymphoma [269]. The technique described in the literature for radioguided SNL biopsy for these other cutaneous malignancies is identical to the technique described for malignant melanoma and Merkel cell carcinoma.

### Gastrointestinal malignancies

#### Colorectal cancer

##### RIGS overview

The concept of RIGS in colorectal cancer [58] was first introduced in 1984 by the work of Aitken and colleagues [289,290] at The Ohio State University (Table 1). Using CEA-producing human colonic adenocarcinoma cells (CX-1) grown as tumor xenografts that were subcutaneously implanted on the flank in Swiss nude mice, they demonstrated the feasibility of gamma probe detection of ^131^I-labeled anti-CEA polyclonal baboon antibodies within such subcutaneous implants and demonstrated the greater sensitivity of the gamma detection probe as compared to gamma camera imaging for small tumor implants [289,290]. In addition to these experimental results in mice, they also reported the first clinical application of RIGS in a case study of a 59 year-old male with a rectal carcinoma [290]. The handheld gamma probe detected an increased level of the ^131^I-labeled anti-CEA polyclonal baboon antibody in the rectal tumor (135 counts/minute) compared to the sigmoid colon (111 counts/minute).

Shortly thereafter, Martin et al. [291] reported the results of the first RIGS clinical series involving 28 patients with primary (n = 12) and recurrent (n = 16) colorectal cancer. Each patient was injected intravenously with 2.2 mCi (81.4 MBq) of ^131^I baboon polyclonal anti-CEA antibody at approximately 48 to 72 hours prior to surgery. Twenty-three patients underwent preoperative scintigraphy. In all patients, intraoperative counts (20 seconds per count) of gross tumor and adjacent tissues were obtained using a prototype handheld gamma detection probe and a commercial control unit. Preoperative scintigraphy yielded correct results in 33% and 64% of the patients with primary and recurrent disease, respectively. In comparison, high intraoperative tumor-to-background ratios were achieved in all patients with primary tumors (3.91:1) and recurrent tumors (4.18:1). This clinical feasibility study demonstrated the ability of RIGS technology to provide immediate intraoperative information for the assessment of colorectal cancer. In addition, this study was an early indication of the application of the inverse square law and the advantage of a gamma detection probe [8]. The inverse square law states the sensitivity or specificity will increase as the distance from the detector is decreased. Hence, the intraoperative use of the gamma detection probe increased the probability of tumor detection due to its proximity to the source of radioactivity.

In all subsequent RIGS clinical studies, ^125^I was selected to replace ^131^I [26,101,292]. ^125^I was selected as the radionuclide of choice for RIGS since the handheld gamma detection probe was more efficient at detecting ^125^I than ^131^I secondary to the lower energy level of the primary gamma photon emitter of ^125^I (35 keV) as compared to the primary gamma photon emitter of ^131^I (364 keV). Such lower energy gamma photons were more likely to be detected because the radiation was less likely to pass through a small detector without being counted. Likewise, in tumor-bearing mice, higher tumor-to-background ratios were achievable with ^125^I labeled antibodies than with ^131^I labeled antibodies [293]. This resultant improved tumor-to-background ratio was a function of greater tissue attenuation and lesser scatter that occurs because of the lower energy gamma photon emission of ^125^I [8,26]. Likewise, the majority of clinical trials for RIGS in the detection of colorectal cancer have specifically utilized ^125^I-labeled anti-TAG-72 monoclonal antibodies. As previously discussed, such anti-TAG-72 monoclonal antibodies have evolved from first generation ^125^I-labeled murine B72.3 monoclonal antibodies, to second-generation ^125^I-labeled murine CC49 monoclonal antibodies, and finally to third-generation ^125^I-labeled humanized CC49 (HuCC49) monoclonal antibodies [49].

##### RIGS with 17-1A murine monoclonal antibody

The availability of the monoclonal antibody 17-1A and its antibody fragment provided preliminary indication of the utility of ^125^I and a gamma detection probe to discriminate between tumor and background tissue and detect occult disease [101]. In 1986, O'Dwyer and colleagues [294] at The Ohio State University reported a 75% intraoperative tumor localization rate among patients with primary and recurrent colorectal cancer. Higher tumor-to-background ratios were observed in patients intravenously injected with the ^125^I-17-1A whole monoclonal antibody (3.4:1) compared to its radiolabeled monoclonal antibody fragment F(ab')_2 _(2.3:1). Of the 16 evaluable patients (two patients excluded due to a probe malfunction), histologically confirmed occult or subclinical disease was detected in 18% of these patients that would have otherwise not been clinically detected by normal intraoperative inspection and palpation. This early study was useful in shedding light on the optimal timing between injection and surgery for enhancing intraoperative gamma detection.

Petty and colleagues [295] at The Ohio State University continued the investigation of the ^125^I-17-1A monoclonal antibody in thirteen patients with colorectal cancer and optimized the use of the gamma detection probe to determine the clearance of the blood-pool background between the time of injection and surgery. The scheduling of the operative procedure was dependent on external precordial counts of ≤ 10 per second obtained by the gamma detection probe. Intraoperative detection by the gamma detection probe was facilitated by the incorporation of a microprocessor into the control unit. The microprocessor maintained a running average of the count rate and made a synthetic siren sound when the count rate exceeds the background count by a statistically significant amount [8]. The squelching feature of the control unit allowed for a siren sound and no sound discrimination between normal tissues and areas of increased radioactivity without interfering with the count rates. Overall tumor localization rate of ^125^I-17-1A monoclonal antibody was 61% [295]. Localization was 33% among primary tumor patients and 85% among recurrent tumor patients. Of the 12 tumor sites localized and biopsied, histologically confirmed tumor was identified in 8 (66%) using hematoxylin and eosin (H&E) staining. Additional studies using immunohistochemical staining and autoradiography identified malignant cells in all specimens. In comparison to the study reported by O'Dwyer et al [294], a higher occult disease detection rate was attributed to a lower blood-pool background at operation and improvements in the gamma detecting probe [295]. Predictability of ^125^I-17-1A monoclonal antibody blood clearance was feasible using precordial counts. The majority of patients (85%) demonstrated adequate clearance by 10 days post-injection of ^125^I-17-1A monoclonal antibody. Of the seven patients with recurrent disease, occult disease was identified in 43% of these patients. The finding of occult disease altered the surgical management (i.e., extended resection) of six patients. This study demonstrated the ability of the RIGS system to provide immediate intraoperative information regarding the extent and localization of disease by identifying tissues that did not look or feel abnormal but which were worthy of attention due to a siren signal/sound generated by the gamma detection probe.

##### RIGS with B72.3 murine monoclonal antibody

The first generation of radiolabeled anti-TAG-72 monoclonal antibodies utilized the B72.3 murine monoclonal antibody. From preclinical studies on human colon carcinoma xenografts, significant tumor uptake of ^131^I-B72.3 monoclonal antibody was found to occur within 48 hours and subsequently increased over time [296]. Although blood-pool background clearance yielded higher tumor-to-background ratios, the activity within the xenograft tumors remained constant over the 19 day period. This prolonged retention of the radiolabeled B72.3 monoclonal antibody and high degree of tumor targeting demonstrated in preclinical studies resulted in the selection of the B72.3 monoclonal antibody for subsequent use in RIGS [296,297].

Martin et al [298] reported the use of the ^125^I-B72.3 monoclonal antibody and an improved gamma detection probe in human clinical trials initiated at The Ohio State University in 1986 [299] (Table 1). Of the 66 patients with gastrointestinal, breast, and ovarian carcinoma, six and 39 had primary and recurrent colon cancer, respectively [298]. Each patient was injected intravenously with 4 to 5 mCi (148 to 185 MBq) of ^125^I-B72.3 monoclonal antibody at approximately 5 to 42 days prior to surgery. Adequate clearance of the blood-pool background yielded a high percentage of tumors identified using RIGS. The mean interval between injection and surgery of 19 days demonstrated long retention of B72.3 monoclonal antibody and adequate amount of the radionuclide for intraoperative gamma probe detection. Tumor localization of ^125^I-B72.3 monoclonal antibody was observed in 83% and 79% of the patients with primary and recurrent colorectal cancer, respectively. Increasing the size of the probe detector crystal and modifications in the crystal mounting improved the counting rate by a factor of approximately 2.5.

Prolonged retention of the radiolabeled B72.3 monoclonal antibody and adequate clearance of the blood-pool background at the time of operation was further demonstrated in a study of primary colon cancer [300]. Thirty primary colon cancer patients were intravenously injected with 1 to 5 mCi (37 to 185 MBq) of ^125^I-B72.3 monoclonal antibody and then underwent surgery after adequate clearance of the blood-pool background was determined by precordial gamma detection probe counts (mean 22.3 days, range 8 to 34 days). The ^125^I-B72.3 monoclonal antibody localized in histologically confirmed tumor among 23 of the 30 patients (77%). Of the 23 patients, 30% had occult disease in the liver, lymph nodes, and/or invasion in adjacent tissues identified by RIGS. In 23% of these patients, the surgical plan was modified due to the finding of occult disease. RIGS provided adequate information (i.e., gamma detection probe counts comparable to background) regarding tumor margins and histologically confirmed benign lesions of the liver and ovaries. Therefore, the RIGS system provided a more accurate intraoperative staging of disease and immediate confirmation of tumor margins.

In 1991, Martin and Carey [301] reported the relationship between the survival of 86 patients with recurrent colorectal cancer undergoing a second-look procedure and use of the RIGS system. All patients received a preoperative intravenous injection of 2 mCi (74 MBq) of ^125^I-B72.3 monoclonal antibody at approximately 24 days prior to surgery. The abdomen and pelvis were explored using traditional surgical techniques of inspection and palpation. Prior to the use of the gamma detection probe, sites of tumor were noted and the surgeon declared his surgical plan. The surgical field was reexplored using the gamma detection probe to identify areas of increased radioactivity compared to normal adjacent tissues and to determine whether the findings would alter the planned procedure. Fifty-three patients (62%) were deemed resectable by traditional techniques. However, only 40 patients (47%) were determined to be resectable by RIGS. Retroperitoneal disease was a common finding for RIGS nonresectability. Two-, three-, four-, and five-year survival data were reported for three classifications of patients: RIGS resectable (n = 40), traditional nonresectable (n = 33), and RIGS nonresectable (n = 13). Overall survival rate for the RIGS resectable group was 83%, versus 21% and 31% for the traditional nonresectable and RIGS nonresectable groups, respectively. Significant differences in survival were observed in the RIGS resectable versus traditional nonresectable, p < 0.0001; RIGS resectable versus RIGS nonresectable, p < 0.0008; and non-significant differences were noted in the traditional nonresectable versus RIGS nonresectable groups (p = 0.24). A two- and five-year survival comparison is provided in Table 4 for RIGS using ^125^I-B72.3 monoclonal antibody.

**Table 4:** Two-year and five-year survival for RIGS with ^125^I-B72.3 monoclonal antibody

PATIENT GROUP	SURVIVAL: 2-YEAR (% PATIENTS)	SURVIVAL: 5-YEAR (% PATIENTS)
RIGS resectable	95%	60%

Traditional nonresectable	36%	0%

RIGS nonresectable	57%	0%

The safety and efficacy of ^125^I-B72.3 monoclonal antibody to localize in tumor and detect occult disease was evaluated in a multicenter clinical trial of 104 patients with primary or recurrent colorectal cancer [302]. All patients received a preoperative an intravenous injection of 2 mCi (74 MBq) of ^125^I-B72.3 monoclonal antibody at approximately 24 days prior to surgery. Tumor localization occurred in 78% of the patients. A total of 30 occult tumor sites were identified in 26 patients. Of all histologically confirmed tumor sites, 9.2% represented clinically occult sites identified only by the gamma detection probe. Location of occult disease in primary patients included retroperitoneum, pelvis, periportal, and liver. Of the 72 patients with recurrent disease, 37 were deemed unresectable. In 27% of these patients, data provided by the RIGS led to the decision to abandon an attempted resection. In addition, the operative resection was extended in the remaining 35 patients due to RIGS positive findings. One patient developed an acute hypersensitivity reaction to the skin test using unlabeled B72.3 monoclonal antibody. Although 40% of the patients injected with ^125^I-B72.3 monoclonal antibody developed human anti-murine antibodies, the occurrence did not impact upon tumor localization. Serum TAG-72 levels analysis demonstrated no relationship between the circulating antigen and localization of ^125^I-B72.3 monoclonal antibody. The safety analysis of ^125^I-B72.3 monoclonal antibody did reveal statistically significant changes in body temperature, systolic blood pressure, and hemoglobin concentration; however, these changes were clinically insignificant. This multi-institutional study confirmed a relatively low toxicity profile for ^125^I-B72.3 monoclonal antibody and its utility in identifying occult disease and impact on the surgical management of patients with primary and recurrent colorectal cancer.

Additional investigations into RIGS with ^125^I-B72.3 monoclonal antibody were reported in 1998 by the group at The University of Genoa in Italy [303,304]. They evaluated RIGS in both 16 asymptomatic patients with a history of previously treated colorectal cancer who has a rising CEA [303] and in 64 patients with recurrent or metastatic colorectal cancer [304]. In the group of 16 asymptomatic patients with a history of previously treated colorectal cancer who has a rising CEA, they found recurrent disease in only nine of 16 patients (56.2%) by traditional surgical exploration alone, but in 14 of 16 patients (87.5%) using a combined approach of traditional surgical exploration and RIGS, thus demonstrating that RIGS detected over-looked recurrent disease in five of 16 patients (31.3%) [303]. In the group of 64 patients with recurrent or metastatic colorectal cancer, they nonrandomly compared ^125^I-B72.3 monoclonal antibody in 30 patients to ^125^I-F023C5 monoclonal antibody fragment that reacts with CEA in 34 patients [304]. They determined that the correct RIGS identification of tumor sites occurred in 80.4% of the ^125^I-B72.3 monoclonal antibody group and in 92.6% of the ^125^I-F023C5 monoclonal antibody fragment group, with additional occult sites of tumor identified by RIGS that would modify surgical strategy in 23.3% of the ^125^I-B72.3 monoclonal antibody group and in only 8.8% of the ^125^I-F023C5 monoclonal antibody fragment group. They concluded that ^125^I-B72.3 monoclonal antibody is the first choice for RIGS in recurrent or metastatic colorectal cancer.

The use of ^111^In-B72.3 murine monoclonal antibody [107,305,306] in radioguided surgery for colorectal cancer has been limitedly investigated. Renda et al [305] investigated the detection of ^111^In-B72.3 monoclonal antibody by RIGS in 8 patients with colorectal cancer, reporting one false-positive and an overall sensitivity of 62.5%. Muxi et al [306] investigated the detection of ^111^In-B72.3 monoclonal antibody by radioimmunoscintigraphy and RIGS in 28 patients with colorectal cancer (18 primary and 10 recurrent) and demonstrated an overall sensitivity of radioimmunoscintigraphy and RIGS of 71.4% and 82.1%, respectively. More recently, Hladik et al [107] investigated the combined use of ^111^In-B72.3 monoclonal antibody and ^99^Tc-anti-CEA monoclonal antibody fragment (^99^Tc-IMMU-4 monoclonal antibody fragment) and compared the findings yielded by preoperative immunoscintigraphy, RIGS, and histology (H & E and immunohistochemistry). Of the 121 patients, 106 and 15 had primary and recurrent colorectal cancer, respectively. A more accurate diagnosis was achieved using preoperative immunoscintigraphy and RIGS. Of the 55 RIGS-positive lymph node patients, 43 cases (78%) were confirmed by histologically, including 9 cases in which RIGS-positivity was recognized by immunohistochemistry alone. Of 66 patients with RIGS-negative results, 62 cases (94%) were confirmed by negative histology. The authors concluded a potential use of RIGS in the surgical management of primary colorectal patients includes better intraoperative assessment of the extent of disease and staging by identifying occult lymph node disease. Nevertheless, the major drawback to the use of ^111^In-B72.3 monoclonal antibody in radioguided surgery for the detection of colorectal cancer has been the nonspecific accumulation of the ^111^In-B72.3 monoclonal antibody conjugate within the liver, thus making it difficult to identify liver metastases and limiting its usefulness to the identification of extrahepatic disease [307].

##### RIGS with CC49 murine monoclonal antibody

The second generation of radiolabeled anti-TAG-72 monoclonal antibodies utilized the CC49 murine monoclonal antibody. CC49 murine monoclonal antibody demonstrated a higher affinity constant (K_a _16.18 × 10^9 ^m^-1^) compared to B72.3 monoclonal antibody (K_a _2.54 × 10^9 ^m^-1^) [72]. In 1992, Arnold et al [74] at The Ohio State University evaluated the efficiency of ^125^I-CC49 monoclonal antibody and its impact on the RIGS system in a clinical trial of colorectal cancer patients, including 24 primary tumors and 30 recurrent tumors. All patients received a preoperative intravenous injection of 2 mCi (74 MBq) of ^125^I-CC49 monoclonal antibody with a varying monoclonal antibody dosage at approximately 14 to 21 days prior to surgery. Tumor localization of the ^125^I-CC49 monoclonal antibody occurred in 86% and 97% of the primary and recurrent tumors, respectively. In comparison to ^125^I-B72.3 monoclonal antibody, ^125^I-CC49 monoclonal antibody tumor localization was superior in targeting primary and recurrent colorectal tumors. The intraoperative findings from RIGS altered the planned surgical procedure in 50% and 47% of the primary and recurrent patients, respectively. Likewise, the intraoperative identification of occult disease by RIGS resulted in upstaging of cancer and abandonment of hepatic resections in three patients undergoing a second-look procedure. In this study, tissue specimens were classified by whether they were detected by RIGS and by the presence or absence of histologically confirmed carcinoma. Specimens were divided into tissue types I through IV, based on antibody localization and hematoxylin and eosin staining: type I, RIGS negative and histologically negative; type II, RIGS negative and histologically positive; type III, RIGS positive and histologically negative; and type IV, RIGS positive and histologically positive. Of interest to the authors were the type III lymph nodes. The intraoperative detection of type III lymph nodes by RIGS was a function of the presence of the TAG-72 within the extracellular environment. At surgery, all RIGS-positive tissue was considered malignant and excised whenever possible. Type III lymph nodes were detected in both primary cases (n = 40 specimens) and recurrent cases (n = 16 specimens). In this regard, Quinlan et al [308] reported a relationship between the presence of CC49 monoclonal antibody in the germinal centers of lymph nodes and early death among patients with colorectal cancer.

The role of RIGS as an intraoperative prognostic indicator of survival in patients with primary colorectal cancer was investigated by Arnold et al [309]. Patients were intravenously injected with 2 mCi (74 MBq) of ^125^I-CC49 monoclonal antibody at approximately 21 days prior to surgery. Thirty-one primary colorectal cancer patients with ^125^I-CC49 monoclonal antibody localization were assessed for the presence or absence of residual RIGS-positive tissue at the completion of the operative procedure. Patients were classified as RIGS-positive (i.e., residual RIGS-positive tissue) or RIGS-negative (i.e., no residual RIGS-positive tissue). Tumor localization was observed in 86% of the patients. One-hundred and nine extra-regional sites of RIGS-positivity were identified using RIGS, including but not limited to the gastrohepatic ligament, celiac axis, retroperitoneum, liver, omentum, and sites above the diaphragm. Seventeen and 14 patients were assessed as RIGS-positive and RIGS-negative, respectively, following the completion of the operative procedure. Follow up of patients ranged from 30 to 54 months. Of the 17 patients with residual RIGS-positive tissue, 15 (88%) succumbed to their disease. In comparison, all 14 patients with no residual RIGS-positive tissue were alive at last follow-up (24 to 48 months after surgery) (p < 0.0001). Therefore, the presence or absence of residual RIGS-positive tissue provided immediate and accurate prognostic information regarding the behavior of the tumor and patient outcomes in patients with primary colorectal cancer.

Likewise, in the recurrent colorectal cancer population, Bertsch et al [310] reported an improved survival relative to the use of the RIGS system. One hundred and thirty-one patients with recurrent colorectal cancer were injected with ^125^I-B72.3 monoclonal antibody (n = 86) or ^125^I-CC49 monocloncal antibody (n = 45) and underwent a surgical exploration using traditional inspection and palpation and the RIGS system. Of the 49 patients deemed resectable (i.e., successful removal of all traditionally evident disease and all RIGS-positive tissue), 55% (27 patients) were alive at 2 to 8 years following surgery, with a minimal follow-up of 28 months. In contrast, only 2.4% of all patients (2 of 84 patients) with unresectable disease were alive at the time of this report. None of the patients determined to be traditionally resectable but unresectable by the RIGS system were alive. A significant increase in survival (p < 0.0001) was observed among patients with recurrent colorectal cancer undergoing a resection of all disease found by a combination of both traditional means and by RIGS.

A long-term survival analysis of 97 patients with primary colorectal cancer supports the use of RIGS as an intraoperative prognostic tool [311]. Survival was assessed using the traditional staging (TNM) and the presence or absence of RIGS-positive tissue at the end of the operative procedure. The mean follow-up among the 52 evaluable patients was 62 months (range 34 to 89 months). Based on TNM staging 13, 18, and 28 patients were Stage, I, Stage II, and Stage III, respectively. By RIGS status, 24 and 35 were RIGS-negative and RIGS-positive, respectively. The RIGS status was not significantly related to the traditional pathologic staging (p = 0.73). No significant difference in survival was observed using standard pathologic staging (p = 0.12). However, a significant difference in survival was observed by the using the RIGS status (p < 0.0002), with 87% of RIGS-negative patients alive and only 40% of RIGS-positive patients alive at a mean follow-up of 62 months.

The extended survival (i.e., 10-year survival) of 90 colorectal cancer patients undergoing RIGS, based on the presence or absence of residual RIGS-tissue at the time of surgery, was recently analyzed by Sun et al [49]. At 10 years after RIGS, survival differences remained significant (p = 0.001), with 49% of the RIGS-negative patients (median survival of 106.5 months) still alive as compared to only 21% of the RIGS-positive patients (median survival of 26 months) still alive. These extended follow-up data support the accuracy of utilizing the RIGS status at the time of surgery as a predictor of long-term survival in colorectal cancer patients.

Additional investigations into RIGS with ^125^I-CC49 murine monoclonal antibody were reported in 2000 and 2001 by the group at Tel-Aviv University in Israel [312-314]. Overall, they evaluated a total of 58 patients with recurrent colorectal cancer who were intravenously injected with of 2 mCi (74 MBq) of ^125^I-B72.3 monoclonal antibody at approximately 24 days prior to surgery. Traditional surgical exploration and RIGS were performed. While traditional surgical exploration identified 117 suspected tumor sites, RIGS identified 177 suspected tumor sites. In 17 of 58 patients (29.3%), at least one occult tumor site that was not identified by traditional surgical exploration was identified by RIGS, and was subsequently confirmed by pathology with H&E staining. This resulted in a major change in the surgical plan in 16 cases. RIGS performance was found to vary between lymphoid tissue and non-lymphoid tissue, with a positive predictive value and a negative predictive value of 96% and 90% in non-lymphoid tissue and 40% and 100% in lymphoid tissue, respectively.

##### RIGS with humanized CC49 monoclonal antibody

A phase I study of ^125^I-HuCC49ΔC_H_2 monoclonal antibody conducted at The Ohio State University determined the feasibility of the humanized monoclonal antibody as a component of the RIGS system and evaluated the optimal timing interval between injection and surgery [315]. Study eligibility included patients with recurrent colorectal cancer undergoing a surgical exploration and excluded those with prior exposure to murine antibodies. Patients underwent a surgical exploration at variable time intervals following the intravenous administration of 2 mCi (74 MBq) of ^125^I-HuCC49 ΔC_H_2 monoclonal antibody or after the precordial counts measured by a handheld gamma detecting probe were less than 30 counts per two seconds. HAMA determinations were obtained at baseline, as well as at 4 to 6 weeks and 12 weeks post-injection of the ^125^I-HuCC49ΔC_H_2 monoclonal antibody. At surgery, traditional exploration (i.e., inspection and palpation) and subsequent RIGS exploration were performed. Suspicious tissues were biopsied or excised to determine the presence of absence of carcinoma. The findings were analyzed to determine the sensitivity and positive predictive value for each modality. Of the twenty recurrent colorectal cancer patients evaluated, the first 15 operative procedures were performed at various time intervals (3, 5, 7, 9, 11, and 13 days) after the injection of ^125^I-HuCC49ΔC_H_2 monoclonal antibody and independent of the precordial counts. In the five remaining patients, the timing of surgery was determined by the demonstration of precordial counts of less than 30 counts per two seconds, indicating optimal clearance of ^125^I-HuCC49 ΔC_H_2 monoclonal antibody from the blood-pool background. This occurred at 10 to 24 days after the injection of ^125^I-HuCC49ΔC_H_2 monoclonal antibody. The adequate clearance of the blood-pool background in these five patients allowed for intraoperative differentiation of RIGS-positive tissue compared to normal adjacent tissue. Among those five patients with optimized clearance conditions based on precordial counts of less than 30 counts per two seconds, 17 and 21 sites were identified using traditional techniques (i.e., inspection and palpation) and RIGS system, respectively. Approximately 90% of the excised tissues identified by traditional techniques and by RIGS were histologically positive for tumor. Of the six sites identified exclusively by RIGS, five were excised and all contained tumor. Table 5 provides the sensitivity of traditional exploration and RIGS exploration using ^125^I-HuCC49ΔC_H_2 monoclonal antibody for identifying recurrent colorectal cancer and the positive-predictive value. No significant HAMA response was detected in these patients. Despite the small number of evaluable patients, the study demonstrated the safety and utility of ^125^I-HuCC49ΔC_H_2 monoclonal antibody in RIGS. The rate of tumor localization and detection of occult disease was comparable to murine CC49 previously reported by Arnold et al [74], but without a detectable HAMA response. The use of HuCC49ΔC_H_2 monoclonal antibody as a component of RIGS technology suggests that it is worthy of continued investigations in colorectal cancer patients.

**Table 5:** Traditional exploration and RIGS exploration with ^125^I-HuCC49ΔC_H_2 monoclonal antibody

	Traditional exploration	RIGS exploration
Sensitivity	64%	92%

Positive predictive value	90%	100%

##### RIGS with ^125^I-anti-CEA monoclonal antibody (^125^I-A_5_B_7_) and ^99^Tc-anti-CEA monoclonal antibody fragment (^99^Tc-IMMU-4)

Limited information is available on use radiolabeled anti-CEA monoclonal antibody in RIGS for colorectal cancer [90,91,107,316]. Dawson et al [91] evaluated 43 patients undergoing surgery with primary colorectal cancer and 9 patients undergoing second look laparotomy for recurrent colorectal cancer who were intravenously injected with 2 mCi (74 MBq) of ^125^I-anti-CEA monoclonal antibody (^125^I-A_5_B_7_) three to ten days before surgery. They determined that RIGS assessment of ^125^I-A_5_B_7_localized in 97.8% of primary tumors and in 88.8% of most principle tumor sites found at the time of the second look procedure. Likewise, they determined that the use of RIGS influenced the overall surgical procedure performed in 2 of 43 cases (4.6%) of primary colorectal cancer and in three of nine cases (33%) of recurrent colorectal cancer. Lechner et al [316] evaluated 20 patients with primary colorectal cancer who were intravenously injected with 25 mCi (925 MBq) of ^99^Tc-anti-CEA monoclonal antibody fragment (^99^Tc-IMMU-4) on the day before RIGS. They determined that RIGS assessment of ^99^Tc-IMMU-4 led to up-staging of disease in 7 of 20 patients (35%). Hladik et al [107] evaluated 65 patients with either primary or recurrent colorectal cancer who were intravenously injected with ^99^Tc-IMMU-4 in a dose range of 18.9 to 24.3 mCi (700 to 900 MBq) on the day before RIGS. They determined that RIGS assessment of ^99^Tc-IMMU-4 led to a sensitivity and accuracy for the detection of tumor or local recurrence of 92% and 92%, for the detection of hepatic metastases of 75% and 92%, and for the detection of extrahepatic metastases of 57% and 78%, respectively. Finally, Gu et al [90] evaluated 29 patients with primary colorectal cancer who were injected with 0.5 mCi (18.5 MBq) of ^125^I-anti-CEA monoclonal antibody (^125^I-CL58) into the colonic submucosa at the tumor-surrounding areas (at 2, 4, 6, 8,10, and 12 o'clock locations) via colonoscopy at a time of approximately three to 14 days before RIGS. The sensitivity of RIGS in detecting primary lesions was 93.1%, and the specificity of RIGS to correctly identify negative surgical margins was 95.5%. For detection of lymph node metastases, the sensitivity and specificity of RIGS was 92.0% and 87.8%, respectively.

##### Radioguided SLN biopsy

The application of SLN biopsy technology to colorectal cancer has been thoroughly studied. While the vast majority of the information available for SLN biopsy for colorectal cancer is on the application of the blue dye alone technique, the radioguided application of ^99m^Tc sulfur colloid technique with intraoperative gamma probe detection has been limitedly investigated [317-324]. The results of the two largest reported series are strikingly divergent [319,323]. In 2003, Bilchik et al [319] evaluated 120 patients using a combined blue dye and ^99m^Tc sulfur colloid (dose not reported) technique in 32 such patients. They reported successfully identifying a SLN in 115 of 120 (96%) patients, with a false-negative SLN biopsy results identified in only 5 of 115 (4%) patients. In contrast, in 2007, Lim et al [323] evaluated 120 patients using a combined blue dye and ^99m^Tc sulfur colloid (0.5 mCi; 19 MBq) technique in all patients. They reported successfully identifying a SLN in 119 of 120 (99%) patients, with a falsely negative SLN biopsy results identified in 20 of 119 (17%) patients. As a consequence of these strikingly different results with regards to the false-negative rate, we can realistically say that at the current time we are no closer to elucidating the clinical relevance of SLN biopsy technology for colorectal cancer surgery.

##### ^18^F-FDG-directed surgery

The most recent application of the gamma detection probe for radioguided surgery in colorectal cancer has been directed towards to identification of ^18^F-FDG-avid tumors [35-39,45,50]. This technique was first described by Desai et al [35,36] at The Ohio State University (Table 1). Fourteen colorectal cancer patients received an intravenous injection of 4.0 to 5.7 mCi (148 to 211 MBq) of ^18^F-FDG at a time of 58 to 110 minutes prior to intraoperative evaluation with the gamma detection probe. Single or multiple tumor foci were correctly identified in 13 of 14 patients with the gamma detection probe as ^18^F-FDG-avid tissue and this correlated to hypermetabolic activity on prior preoperative diagnostic ^18^F-FDG PET imaging. These results have been further corroborated since that time in several other clinic reports [37-39,45,50]. Most recently, a combined approach of preoperative diagnostic ^18^F-FDG PET imaging and intraoperative gamma probe detection has been advocated to potentially provide the surgeon with a real-time, intraoperative roadmap for accurately locating and determining the extent of tumor recurrence in patients with colorectal cancer [50]. In this series, patients received an average dose of 10 to 15 mCi (370 to 555 MBq) of ^18^F-FDG at a time of 30 to 60 minutes prior to intraoperative evaluation with the gamma detection probe. It was determined that intraoperative evaluation with the gamma detection probe appeared to be more sensitive in detecting the extent of abdominal and pelvic recurrence, while preoperative ^18^F-FDG PET imaging was more sensitive in detecting liver metastases and other distant metastases [50].

#### Anal cancer

The application of the gamma detection probe in radioguided surgery for anal cancer has been limited to radioguided SLN biopsy [324-337]. In general, ^99m^Tc sulfur colloid, ^99m^Tc colloidal human albumin, ^99m^Tc colloidal rhenium sulphide, and ^99m^Tc antimony trisulfide colloid have been utilized and are injected into four subdermal or submucosal sites around the primary tumor in a total dosage range from 0.135 mCi (5 MBq) to 1.0 mCi (37 MBq). Localization to a SNL was generally demonstrated in 75% to 100% of cases. There were three common pathways of lymphatic drainage (including drainage to inguinal, iliac, and mesorectal lymphatic basins) and the very frequent finding of bilaterality of such lymphatic drainage, emphasizing the importance of preoperative lymphoscintigraphy in most effectively performing radioguided SLN biopsy in anal cancer. The inguinal region was the predominant lymphatic drainage pathway, representing the site of localization most accessible to performance of a minimally invasive radioguided SLN biopsy procedure. Radioguided SLN biopsy to the inguinal region was useful in identifying inguinal lymph node metastases in approximately 10% to 40% of such patients. However, there is yet to be a large scale prospective clinical trial to assess the clinical efficacy of radioguided SLN biopsy in anal cancer.

#### Esophageal cancer

The application of the gamma detection probe in radioguided surgery for esophageal cancer has been limited to radioguided SLN. In this regard, it is interesting to note that there have been as many review papers written that have debated the pros and cons of the potential clinic efficacy of radioguided SLN biopsy for esophageal cancer [324,338-343] as there have been actual papers written on the clinical results of this technology in esophageal cancer surgery. Clinical data on radioguided SLN biopsy for esophageal cancer are restricted to those reports from Japan [344-350], the United Kingdom [351], and Germany [352]. Central to the debate with regards to the potential clinical efficacy of radioguided SLN biopsy for esophageal cancer are the complex and extensive lymphatic networks located throughout cervical, mediastinal, and abdominal nodal basins and the concern for skip metastases [324,338,339,341-343]. In this regard, the routine use of radioguided SLN biopsy for esophageal cancer surgery remains controversial and is yet to be widely adopted.

Most frequently, ^99m^Tc tin colloid has been used as the radiocolloid, especially in Japan [344-350]. However, ^99m^Tc colloidal human albumin [351], ^99m^Tc sulfur colloid [352], and ^99m^Tc colloidal rhenium sulfide [347] have also been utilized. The dosing of these radiocolloid agents vary from as low as 0.54 mCi (20 MBq) [351] to as high as 5.0 mCi (185 MBq) [346,350]. These radiocolloid agents are injected endoscopically in up to four submucosal sites around the tumor on either the day before surgery [344-350] or on the day of surgery [351,352].

The two largest series reported are by Lamb et al [351] and Kato et al [347]. In 2005, Lamb et al [351] identified a SLN in all 40 patients evaluated with adenocarcinoma of the lower esophagus. They identified a total of 77 SLNs from a combination of both the mediastinal and abdominal nodal stations, with 51% of these SLNs being located within the mediastinal nodal stations and with 38% of proven metastatic lymph nodes residing within the mediastinal nodal stations. Noteworthy from this study was the fact that if patients did not have a mediastinal SLN identified at the time of radioguided SLN biopsy that a negative abdominal SLN accurately predicted the absence of mediastinal lymph node involvement. Such a predictive property of SLN biopsy may help to alter the surgical strategy by possibly omitting a cervical nodal dissection for mid-esophageal tumors or by making the option of transhiatal esophagectomy more appropriate when mediastinal nodal clearance is not indicated. In 2003, Kato et al [347] identified a SLN in 23 of 25 (92%) patients evaluated with squamous cell carcinoma of the thoracic esophagus. The accuracy of radioguided SLN biopsy was 91.3% (21 of 23 patients), the sensitivity was 86.7% (13 of 15 patients), and the false-negative rate was 8.7% (2 of 23 patients). Despite the promising clinical results seen with radioguided SLN biopsy for esophageal cancer, technical difficulties can be encountered with regards to identifying SLNs within the peritumoral nodal stations, particularly the lower paraesophageal, paracardial, and left gastric nodes, which are common sites of lymphatic metastases for lower esophageal cancers. As with other malignancies, the proximity of the SLN to the radiocolloid injection site creates an artifact shine through effect which can make identification of such located SLN with the gamma detection probe very difficult.

#### Gastric cancer

Although radioguided surgery is not routinely utilized in the current surgical management of gastric cancer, the application of the gamma detection probe for gastric cancer surgery has been investigated in the areas of radioguided SLN biopsy, RIGS, and ^18^F-FDG-directed surgery.

##### Radioguided SLN biopsy

The necessary extent of lymphadenectomy during gastric cancer surgery remains a controversial issue, with two randomized trials demonstrating no survival advantage for patients treated with D2 lymphadenectomy as compared to a D1 lymphadenectomy [353,354] compared with other retrospective reviews showing a survival benefit for patients treated with D2 lymphadenectomy [355,356]. In this regard, the potential application of SLN biopsy for gastric cancer remains an active avenue of clinical research and debate amongst surgeons who treat gastric cancer. This is most relevant for those clinicians in eastern Asia that primarily treat early stage gastric cancers, which by definition should have a less than 10% chance of nodal involvement [357].

Multiple reports exist in the literature on the feasibility of radioguided SLN biopsy for gastric cancer [338,344,345,358-383]. Many of these reports from eastern Asia specifically target a purely laparoscopic approach to radioguided SLN biopsy in the treatment of cases of early stage gastric cancer [338,361,367,371,377,378,380-382].

Most frequently, ^99m^Tc tin colloid [338,344-346,359-361,363-367,369,371,375-378,380,382] has been used as the radiocolloid, especially in eastern Asia. However, ^99m^Tc colloidal rhenium sulfide [362,368,374,379], ^99m^Tc sulfur colloid [352,358,370,372], and ^99m^Tc colloidal human albumin [381] have also been utilized. The dosing of these radiocolloid agents vary from as low as 0.5 mCi (18.5 MBq) [374] to as high as 6.0 mCi (222 MBq) [364,369]. These radiocolloid agents are generally injected endoscopically in up to four submucosal sites around the tumor at a time period from two to 24 hours before surgery.

The two largest series reported are by Kitagawa et al [359] and Uenosono et al [369]. In 2002, Kitagawa et al [359] identified a SLN in 138 of 145 patients (95.2%) with presumed cT1N0 or cT2N0 gastric cancer. A SLN was positive in 22 of 24 patients who had lymph node metastases and this demonstrated a diagnostic accuracy of assessment of the regional lymph node status on the basis of the SLN status of 98.6%. In 2005, Uenosono et al [369] identified a SLN in 99 of 104 patients (95.2%) with presumed cT1 or cT2 gastric cancer. Excluding three technical failures in radiocolloid injection, identification rates were 99% (78 of 79) and 95% (21 of 22) for cT1 and cT2 lesions, respectively. Lymph node metastases and/or micrometastases were found in 28 patients (15 cT1 and 13 cT2) and the resultant false-negative rate, sensitivity, and accuracy were significantly better for cT1 tumors than for cT2 tumors (P < 0.001, P = 0.004, and P < 0.001, respectively). While the possibility exists for radioguided SLN biopsy to help individualize the surgical therapy for patients with early stage gastric cancer, additional studies are needed to determine its utility.

##### RIGS

The feasibility of RIGS for gastric cancer has been evaluated by several groups of investigators [149,383-387]. In 1988, Martin et al [149] evaluated ^125^I-B72.3 murine monoclonal antibody in five patients with gastric cancer, finding positive gamma detection probe counts in four patients. In 1994, Xu et al [383] and Lui et al [384] evaluated ^131^I-3H11 murine monoclonal antibody, a mouse antibody raised against human gastric cancer cells, in 25 patients with gastric cancer. They reported detection of metastatic lymph nodes with a sensitivity of 99.2%, a specificity of 97.7%, and an accuracy of 98.8%, and reported the detection of tumor infiltration of the gastric wall with a sensitivity of 94.6%, a specificity of 96.7%, and an accuracy of 95.9%. In 1998, Lucisano et al [385] evaluated ^125^I-B72.3 murine monoclonal antibody in 7 patients with gastric cancer. The correct RIGS identification of the primary tumor was seen in four of seven patients (57.1%) and of metastatic lymph nodes in two of four patients (50%). Also, in 1998, Mussa et al [386] evaluated ^111^In-B72.3 murine monoclonal antibody in 3 patients with gastric cancer, confirming intraoperative tumor-to-background counts of greater than 2 in all three cases and demonstrating a sensitivity of 100%, a specificity of 72%, and no false-negative findings with RIGS. Finally, in 2000, Wang et al [387] evaluated ^125^I-3H11 murine monoclonal antibody in 35 patients with gastric cancer. For the detection of lymphatic metastases, they demonstrated a sensitivity of 83.6%, a specificity of 95.0%, and an accuracy of 91.3%. The existence of lymphatic micrometastatic disease was verified immunohistochemically in 10 of 19 patients (52.6%) that were RIGS-positive but that had H&E histologically negative lymph nodes. Despite these early promising results, no further investigations into RIGS for gastric cancer have been published since that last report.

##### ^18^F-FDG-directed surgery

The use of ^18^F-FDG-directed surgery has been very limitedly reported in the literature for the surgical management of gastric cancer [45,47]. Gulec et al [45] reported on a single case of gastric cancer in which ^18^F-FDG-directed surgery was utilized for the identification and resection of a hypermetabolic metastatic lymph node during a gastectomy and extended node dissection. Piert et al [47] reported on the use of ^18^F-FDG-directed surgery in three gastric adenocarcinomas and two adenocarcinomas of the gastroesophageal junction.

#### Pancreatic cancer

Radioguided surgery using the gamma detection probe has surprisingly been previously investigated in only the most limited fashion for pancreatic cancer [388]. No data are currently available on radioguided sentinel lymph node biopsy or on ^18^F-FDG-directed surgery for pancreatic adenocarcinoma. Only a single report exists in the literature from 1997 by LaValle et al [388] from The Ohio State University on RIGS for the assessment of extent of disease in ten cases of pancreatic adenocarcinoma that were deemed resectable by preoperative CT scan. Each patient was intravenously injected with 2 mCi (74 MBq) of ^125^I-CC-49 murine monoclonal antibody and then underwent surgery after adequate clearance of the blood-pool background was determined by precordial gamma detection probe counts (mean 26.1 days, range 7 to 35 days). Traditional assessment of the abdomen was compared to RIGS assessment of the abdomen. Three patients underwent pancreatic resection for locoregional disease. The other seven patients had visceral metastases, carcinomatosis, or both detected at the time of laparotomy. All sites suspicious for tumor by traditional assessment of the abdomen were found to be RIGS positive. Occult pancreatic adenocarcinoma identified only by RIGS assessment was found to be disseminated to both the abdominal viscera and lymphatics. RIGS detected significantly more total sites (viscera and lymphatics) of metastatic disease than traditional assessment (73 site versus 31 sites for RIGS versus traditional assessment, respectively, p < 0.05), with the greatest difference being observed for dissemination to the lymphatics (44 site versus 6 sites for RIGS versus traditional assessment, respectively, p < 0.001). Despite these very encouraging early results, no further work has been done on RIGS for pancreatic cancer.

#### Gastointestinal stromal tumors (GIST)

The use of the gamma detection probe in radioguided surgery for GIST is virtually nonexistent. Only one report exists in the literature that describes two GIST cases in which ^18^F-FDG-directed surgery was employed [45]. In this report, ^18^F-FDG-directed surgery was used during exploratory cytoreduction and during liver resection for GIST.

### Head and neck malignancies

#### Squamous cell cancer of the oral cavity, oropharynx, hypopharynx, and laryngeal regions

##### Radioguided SLN biopsy

While SLN biopsy had become a widely accepted diagnostic technique in the evaluation of the lymph node status in breast cancer and melanoma, it has been met with much more limited enthusiasm for squamous cell cancers of the head and neck region, including the oral cavity, oropharynx, hypopharynx, and laryngeal region, secondary to the complicated lymphatic drainage pathways of the head and neck region [389]. In 1996, Alex and Krag [390] first described successful radiolocalization of SLNs with ^99m^Tc sulfur colloid in the aerodigestive system in a patient with supraglottic squamous cell cancer. Since the time of this initial report, the sensitivity and specificity of radioguided SLN biopsy has become greater than that of physical exam, computed tomography, magnetic resonance imaging, or positron emission tomography for assessing the N0 neck [391]. Additionally, the overall feasibility of radioguided SLN biopsy has gradually improved, with most recently reported success of localization of 99.3% in 137 patients by American College Of Surgeons Oncology Group Z0360 [392] and 100% in 79 patients by Stoeckli [393] for early (T1/T2) squamous cell cancers of the oral cavity and oropharynx.

The overall methodology for performing radioguided SLN biopsy for squamous cell cancers of the head and neck region, including the oral cavity, oropharynx, hypopharynx, and laryngeal region is not that dissimilar as to that for breast cancer and melanoma. However, there are some unique features that are worth further discussing [392-397].

Recently, Vigili et al [396] have outlined one such protocol for performing radioguided SLN biopsy for squamous cell cancers of the oral cavity and oropharynx. Topical anesthetic (10% lidocaine spray) was administered to the oral cavity. Then, 0.8 mCi (30 MBq) to 1.4 mCi (50 MBq) of ^99m^Tc human nanocolloidal albumin within 0.3 ml of normal saline solution was injected superficially into the subepithelial stroma in four points around the tumor. Injection into deeper tissues resulted in a poorer image quality, increased blood accumulation of the tracer, and a lower success rate for SLN localization. The mouth was immediately washed out in order to prevent pooling and swallowing of any residual ^99m^Tc human nanocolloidal albumin. Immediate dynamic lymphoscintigraphy imaging and 30 minute static images were performed with lateral and/or anterior views. The skin overlying the area of the identifiable SLNs seen on lymphoscintigraphy were marked with a permanent marking pen. Subsequent appropriate surgery and radioguided SLN biopsy with the gamma detection probe were performed approximately three hours after completion of lymphoscintigraphy. They defined a SLN as any lymph node containing activity counts of at least three times that of the background count activity.

Likewise, recently, Tomifuji et al [397] have outlined one such protocol for performing radioguided SLN biopsy for squamous cell cancers of the hypopharynx and laryngeal region. On the day before surgery, topical anesthetic (4% lidocaine spray) was administered to the oral cavity. Then, a 2.0 mCi/mL (74 MBq/mL) solution of ^99m^Tc phytate was submucosally injected in 0.2 mL quantities into three to four sites adjacent to the tumor using a 23-gauge endoscopic puncture needle that was passed through the channel of a flexible laryngohypopharyngeal endoscope with a transparent hood. Three hours after the injection, static lymphoscintigraphy images were obtained from lateral and anterior views. The skin overlying the area of the identifiable SLNs seen on lymphoscintigraphy were marked with a permanent marking pen. The following day, appropriate surgery and radioguided SLN biopsy with the gamma detection probe were performed. They defined a SLN as any lymph node containing activity counts of at least ten times that of the background count activity.

The determination of the adequacy of the intraoperative assessment of the neck with the gamma probe during radioguided SLN biopsy for squamous cell cancers of the oral cavity and oropharynx has been recently assessed. In a recent study of 31 patients undergoing radioguided SLN biopsy, resection of the primary tumor, and a concomitant neck dissection, Atula et al [398] reported that all patients could be accurately staged based upon the findings within the hottest three SLNs removed.

Despite encouraging results, controversy still exists as to whether SLN biopsy will ultimately replace the standard clinical practice of selective neck dissection for squamous cell cancers of the head and neck region, as previously described [399,400]. Potential disadvantages of SLN biopsy include a limited exposure and the potential injury to neural structures, such as cranial nerve XI and the mandibular branch of cranial nerve VII [401]. Additionally, if neck nodal metastases are not identified with frozen section, then a subsequent operation within a recently open and inflamed surgical field could increase morbidity [393]. Furthermore, it has been observed that lymph nodes predominately replaced by metastatic disease do not accumulate radiotracer and may result in altered lymphatic pathways, thereby increasing false negatives [402]. Finally, the proximity of the primary tumor and the SLN can be problematic. Kovács et [401] al reported that 6 of 104 known sites of radiotracer localization seen on preoperative lymphoscintigraphy were intraoperatively missed using the gamma detection probe secondary to shine-through radioactivity from a nearby injection site. This proximity issue likely explains why the radioguided SLN identification frequency is less for carcinomas of the floor of mouth as compared to other sites of carcinomas within the oral cavity, oropharynx, hypopharynx, and laryngeal region [392,395-397].

Regardless of these potential disadvantages, radioguided SLN biopsy has proven value especially in management of the N0 neck in squamous cell cancers of the head and neck region [278,392,395,396]. One advantage of radioguided SLN biopsy is for guiding selective nodal harvesting in carcinomas situated at or adjacent to the midline, since in such situations the lymphatic drainage may be bilateral in up to 10% of cases [403]. In this setting, lymphoscintigraphy and intraoperative gamma probe detection of SLNs may provide a suitable alternative in these patients, in whom the management of the contralateral neck is debatable. Another advantage of SLN biopsy is the allowance of a concentrated and extensive pathologic examination of a limited number of lymph nodes versus that of a limited pathologic examination of numerous lymph nodes from selective neck dissections where finding a metastasis is similar to the proverbial needle in a haystack [392]. Because of this required meticulous histopathologic examination, Kovács [404] has discouraged the use of frozen section, but admits its intraoperative availability and diagnostic accuracy are important in shortening the time to deciding on a therapeutic neck dissection. SLN biopsy has been reported to aid in the identification of skip metastases, defined by Byers et al [405] as metastases that bypass level I and II lymph nodes and go directly to levels III through V. They demonstrated that skip metastases were the only manifestation of disease in the neck after selective neck dissection in approximately 16% of 277 patients evaluated. Such a finding can clearly explain disease relapse after surgeries which do not include all five levels. A final potential benefit of radioguided SLN biopsy is the upstaging of early tumors from N0 to N1 which occurs in 16% to 34% [392,395] of patients with early squamous cell carcinomas (T1/2) of the oral cavity and oropharynx. This finding has led to appropriate therapy of the neck (i.e., neck dissection or radiation), and unlike in melanoma where lymphatic metastases portend an extremely poor prognosis, squamous cell carcinomas of the head and neck region with lymphatic metastases remain potentially curable.

##### RIGS

Only one available report can be found in the literature on the application of RIGS to squamous cell cancers of the head and neck region [406]. In this report, Argenzio et al described the intravenous administration of 15 mCi (555 MBq) of ^99m^Tc-labeled anti-CEA monoclonal antibody fragments in two cases with squamous cell cancer of the head and neck, with one to the cheek and one to the scalp [406]. Diagnostic gamma camera imaging was performed 4 and 12 hours after intravenous administration of ^99m^Tc-labeled anti-CEA monoclonal antibody fragments and then intraoperative gamma probe detection was used to assist in the resection of the primary tumor and the assessment of surgical margins at a time approximately 36 hours after the initial intravenous dosage of this radiopharmaceutical. They defined tumor to healthy tissue background ratio of greater than two as a discriminate value for defining diseased tissue.

##### ^18^F-FDG-directed surgery

Only one report is currently available in the literature on the application of gamma probe detection during ^18^F-FDG-directed surgery to squamous cell cancer of the head and neck region [45]. In this report, Gulec et al [45] describe a single case in which ^18^F-FDG-directed surgery was used the localize a nonpalpable target from a head and neck cancer at approximately 4 hours after intravenous injection of a unknown dose of ^18^F-FDG. In an additional report by Meller et al [44], they describe the use of a high energy gamma detection probe to preoperatively identify metastatic lymph nodes in 36 patients with cancers of the oral cavity and oropharynx which was performed within a two week period prior to their definitive surgery and specifically at the time of their diagnostic whole body PET scan. Patients were intravenously injected with 6.8 mCi (250 MBq) to 9.5 mCi (350 MBq) of ^18^F-FDG for the purpose of the preoperative diagnostic whole body PET scan and for the formalized preoperative survey of the lymph node levels within the bilateral neck with a high energy gamma detection probe. Nevertheless, this high energy gamma detection probe was not utilized within the intraoperative setting during their definitive surgical procedure.

#### Parathyroid disease

Aside from radioguided SLN biopsy for breast cancer and melanoma, the gamma detection probe has not been utilized more frequently in any other arena than it has in minimally-invasive radioguided parathyroid surgery for primary hyperparathyroidism. The traditional surgical approach to primary hyperparathyroidism has included bilateral neck exploration with visualizing all parathyroid tissue and removing the enlarged gland(s). In the hands of an experienced parathyroid surgeon, this approach has a success rate of over 95% [407]. However, since a single adenoma is responsible for at least 85% of all cases of primary hyperparathyroidism, the use of bilateral neck exploration has been deemed by some surgeons to represent vast over treatment of such cases. Coupled with advances in preoperative localization of parathyroid tissue with preoperative ^99m^Tc-MIBI imaging and intraoperative quick parathyroid hormone assay, minimally-invasive radioguided parathyroid surgery has become a viable and widely accepted alternative to that of traditional bilateral neck exploration in appropriately selected cases.

##### Minimally-invasive radioguided parathyroidectomy for primary hyperparathyroidism secondary to parathyroid adenomas

In 1984, Ubhi et al [408] first described the use of the gamma detection probe for intraoperative identification of ^201^Tl-thallous chloride in a case of mediastinal parathyroid adenoma (Table 1). Then, in 1995, Martinez et al [409] first described the use of the gamma detection probe for intraoperative identification of ^99m^Tc-MIBI in patients with parathyroid gland pathology (Table 1). Later in 1997, the University of South Florida group [410,411] popularized this technique of using ^99m^Tc-MIBI for the surgical management of primary hyperparathyroidism (Table 1). Since that time, multiple other groups of investigators have published reports describing their experience with minimally-invasive radioguided parathyroidectomy for primary hyperparathyroidism [412-431].

In their initial report, Norman and Chheda from The University of South Florida [410] described their technique of minimally-invasive radioguided parathyroidectomy in 15 patients and utilized an approach of same-day MIBI imaging and radioguided parathyroid surgery. Patients were intravenously injected with 20 to 25 mCi (740 to 925 MBq) of ^99m^Tc-MIBI [411]. After completion of preoperative scintigraphic localization of the presumed parathyroid adenoma (at approximately 3 hours after the MIBI injection), patients underwent radioguided parathyroid surgery. A gamma detection probe was then used to systematically assess radioactivity in all four quadrants of the neck. An initial 2 cm skin incision was then made overlying the location of the radioactive parathyroid gland, the platysma muscle and strap muscles were retracted, and dissection proceeded with guidance from the gamma detection probe for identification of the radioactive parathyroid gland representing the presumed parathyroid adenoma. After resection of the radioactive gland, counts were recorded of the four quadrants of the neck, along with excised radioactive parathyroid gland, excised fat, and excised lymph nodes. Parathyroid adenomas were consistently found to have counts exceeding the postexcisional background counts by more than 20%, whereas the counts of fat and lymph nodes never exceeded 3% of the postexcision background counts [411]. This finding has subsequently led to the abandonment of frozen section pathology for confirmation of the presence of parathyroid tissue [411]. In their initial report [410], a single parathyroid adenoma was located by this technique in 14 of 15 patients, with an average time of approximately 19 minutes to find the radioactive parathyroid gland and an average total operating time of approximately 48 minutes. The one failed patient was intraoperatively found to have four-gland hyperplasia as diagnosed by increased background counts after successful excision of the first targeted radioactive parathyroid gland. The first 5 procedures were done under general anesthesia, with all subsequent procedures being done under local anesthetic.

Instead of utilizing a same-day protocol for MIBI imaging and radioguided parathyroid surgery, other investigators have described and recommended utilizing separate days for MIBI imaging and radioguided parathyroid surgery in order to allow for more efficient use of operative time by preselecting those individuals with confirmed localization to a solitary parathyroid adenoma on MIBI imaging for determination of the appropriateness of minimally-invasive radioguided parathyroidectomy [412,419]. Flynn et al [412] previously described performing preoperative MIBI imaging electively before the day of surgery and subsequent intravenous injection of 20 mCi (740 MBq) of ^99m^Tc-MIBI on the day of surgery at a time approximately 60 to 90 minutes preoperatively. In order to decrease the radiation dose at the time of surgery, Rubello and colleagues have developed a low dose ^99m^Tc-MIBI technique [418-424]. As originally described, Rubello et al [419] reported performing preoperative double-tracer (^99m^Tc-pertechnetate and ^99m^Tc-MIBI) subtraction scanning at a time several days before the proposed surgery to identify those individuals with a presumed solitary parathyroid adenoma and then, on the day of surgery, those individuals who preoperatively localized were subsequently intravenously injected with a low dose of 1 mCi (37 MBq) of ^99m^Tc-MIBI just a few minutes prior to the start of surgery. Both the Flynn et al series [412] and the Rubello et al series [419] reported excellent intraoperative localization rates with the gamma detection probe for demonstrating an abnormal parathyroid gland. Additionally, with the low dose ^99m^Tc-MIBI technique that was administered just a few minutes prior to the start of surgery, Rubello et al [419] demonstrated that all excised parathyroid adenomas had ex vivo radioactivity that was more than 40% of the postexcision background counts, further confirming completeness of surgical excision beyond that of the 20% rule previously established by Murphy and Norman [411]. Finally, both the Flynn et al series [412] and the Rubello et al series [419] used an intraoperative quick parathyroid hormone assay to aid in the surgical decision-making process. While Rubello and colleagues [419-422,424] and Chen et al [428] have strongly advocated the routine use of the intraoperative quick parathyroid hormone assay for further confirming completeness of removal of all hyperfunctioning parathyroid tissue (especially to aid in the recognition of previously unrecognized double adenomas or multiple-gland hyperplasia), Flynn et al [412], Goldstein et al [427], Caudle et al [429], and Norman and Politz [430] have not. In their series of 112 patients with preoperative localization on an MIBI scan, Goldstein et al [427] reported a 98% success rate for identification of a radioactive parathyroid gland based upon the intraoperative use of the gamma detection probe alone during minimally-invasive radioguided parathyroid surgery.

Several authors have discussed the potential advantages of a minimally-invasive radioguided parathyroid surgical approach as compared to standard bilateral neck exploration for the surgical management of primary hyperparathyroidism. Rubello and colleagues [418-424] have emphasized several potential advantages of a minimally-invasive radioguided parathyroid surgical approach for the surgical management of primary hyperparathyroidism. This includes minimizing the invasiveness of the surgical approach for identifying and resecting solitary parathyroid adenomas (thus allowing for maximal cosmetic outcome and the ability to perform such cases without the need of general anesthesia), as well as enhancing the accuracy of the surgeon to specifically locate ectopic parathyroid adenomas and ensure complete operative success by intraoperatively evaluating the postresectional field for residual radioactivity. Additionally, Flynn et al [412] has emphasized a modest cost savings of almost one thousand dollars per patient, particularly due to shorter operative time, avoidance of general anesthesia, elimination of the need for both frozen section pathology and intraoperative quick parathyroid hormone assays, and earlier hospital discharge. A similar effect on cost was reported by Goldstein et al. [413] in which they described a reduction in hospital charges by nearly 50% for the minimally-invasive radioguided parathyroid surgical approach as compared to that of standard neck exploration. Furthermore, a potential benefit of a minimally-invasive radioguided parathyroid surgical approach for reoperative parathyroid surgery has been reported in cases where a failed initial surgical exploration without the use of the gamma detection probe resulted from what was labeled as a "false-positive" MIBI scan [414]. In this series of 17 patients in which a minimally-invasive radioguided parathyroid surgical approach was utilized for reoperative parathyroid surgery, Norman et al [414] reported that a repeat MIBI scan again demonstrated the same focus of radioactivity and intraoperative use of the gamma detection probe correctly identified and aided in the excision of a radioactive parathyroid gland in all 17 patients who were previously presumed to have an initial "false-positive" MIBI scan.

Throughout the development of minimally-invasive radioguided parathyroid surgery, identification of an adenoma on preoperative MIBI scintigraphic imaging has been the most important criteria for the determination of the appropriateness of this surgical approach. However, if an initial negative MIBI scan is encountered in a patient with primary hyperparathyroidism, there may still be a viable role for consideration of MIBI-directed intraoperative gamma probe detection at the time of minimally-invasive radioguided parathyroid surgery. Lal and Chen [431] recently described an algorithm for patients with primary hyperparathyroidism in whom an initial negative MIBI scan is encountered. In a group of 90 such patients, this algorithm involved the use of further preoperative testing with thallium subtraction scanning and neck ultrasound, as well as intraoperative bilateral internal jugular sampling of parathyroid hormone, along with intraoperative quick parathyroid hormone assay, and/or intraoperative MIBI-directed gamma probe detection. Their results indicated that despite an initial negative MIBI scan, that 67% of such patients had a single adenoma as the cause of their primary hyperparathyroidism, and 23% of such patients had successful utilization of intraoperative MIBI-directed gamma probe detection at the time of minimally-invasive radioguided parathyroid surgery.

##### Radioguided parathyroidectomy for hyperparathyroidism secondary to hyperplastic parathyroid glands

While the role of utilizing intraoperative gamma probe detection during radioguided parathyroid surgery is well established for parathyroid adenomas, few reports have focused on its effectiveness for hyperplastic parathyroid glands demonstrated in either primary or secondary/tertiary hyperparathyroidism [432-440]. This approach was first successfully reported in 2000 by Rossi et al [432] in 11 patients with persistent hyperparathyroidism undergoing reoperative neck exploration for localizing residual hyperfunctioning parathyroid tissue and by Navarra et al [433] in a single patient with recurrent secondary renal hyperparathyroidism. The largest series to date has been reported by Chen et al [436], in which they compared the results of minimally-invasive radioguided parathyroid surgery in 25 patients with secondary/tertiary hyperparathyroidism with that of 77 patients with primary hyperparathyroidism. Their results demonstrated in vivo counts of parathyroid adenomas and hyperplastic parathyroid glands did not significantly differ; however, ex vivo counts were highest in single gland adenomas and lowest in hyperplastic parathyroid glands. Nevertheless, all hyperplastic parathyroid glands still registered ex vivo counts > 20% of postexcision background counts in a setting where each patient received an intravenous dosage of 10 mCi (370 MBq) of ^99m^Tc-MIBI at approximately 30 to 60 minutes before surgery. Despite this, Rubello et al [424] still recommend use of the intraoperative quick parathyroid hormone assay at the time of radioguided parathyroidectomy by the low dose ^99m^Tc-MIBI technique in order to establish the completeness of removal of additional foci of hyperfunctioning parathyroid tissue secondary to the persistence of previously unrecognized hyperplastic parathyroid glands.

##### Radioguided surgical approach to recurrent parathyroid cancer

Parathyroid cancer is an extremely rare entity that has been reported to occur in 0.1% to 0.4% of patients with primary hyperparathyroidism [441]. The incidental finding of parathyroid cancer at the time of minimally-invasive radioguided surgery for primary hyperparathyroidism presumed secondary to a parathyroid adenoma is well-described in the literature [424]. Nevertheless, only one report in the literature exists which describes the use of an intraoperative gamma probe detection of ^99m^Tc-MIBI-avid tissue during radioguided surgery that is specifically directed toward parathyroid cancer [442]. In this report, they describe a case of recurrent parathyroid carcinoma in which the patient was intravenously injected with 10 mCi (370 MBq) of ^99m^Tc-MIBI at one hour prior to surgery and an intraoperative gamma detection probe was used to localize and aid in the resection of the area of recurrent disease. In this case, successful resection was verified by normalization of the postresection parathyroid hormone level on intraoperative quick parathyroid hormone assay and for which they report that the patient remains asymptomatic at 17 months after surgery.

#### Thyroid cancer

Radioguided surgery for thyroid cancer has gained some attention in recent years. While radioguided SLN biopsy has been limitedly investigated [443], the mainstay of radioguided surgery for thyroid cancer has been directed towards the utilization of iodine-based and ^99m^Tc-labeled radiopharmaceutical agents for identifying recurrent disease in both differentiated thyroid cancer and medullary thyroid cancer.

Despite the standard practice of using total thyroidectomy and selective ^131^I remnant ablative therapy for the initial therapeutic strategy for differentiated thyroid cancer, locoregional neck recurrence occurs in approximately 5% to 20% of such patients [444-446]. The application of additional courses of ^131^I ablative therapy alone has not proven adequate for the control of disease recurrence of differentiated thyroid cancer [444]. In this regard, radioguided surgery offers a mechanism to achieve complete tumor extirpation of recurrent or persistent disease with a relatively high degree of sensitivity and specificity. Such a technique is important in aiding in the detection of occult disease not readily apparent from a preoperative diagnostic evaluation that may be closely approximated to vascular structures or that is found within areas of scar tissue and sclerosis resulting from previous surgeries, external beam radiation therapy, or high-dose ^131^I ablative therapy [446]. This approach allows for the systematic evaluation of the completeness of surgical resection in extended operations for recurrent or persistent disease, particularly in anatomically difficult areas [447].

Numerous radiopharmaceutical agents have been described for radioguided surgery for recurrent thyroid cancer. The most logical choices for recurrent differentiated thyroid cancer would include ^131^I and ^123^I. However, a previous history of ^131^I ablative therapy has been shown to abolish subsequent uptake of radioiodine in approximately 65% of previously ablated patients with recurrent differentiated thyroid cancer [448]. These iodine-negative recurrent tumors, being resistant to further ^131^I therapy, carry a worse prognosis, with reported 2-, 5-, and 10-year survival rates of 55%, 16%, and 11%, respectively, compared to 91%, 77%, and 62%, respectively, for patients with iodine-avid recurrent tumors [449,450]. In this regard, 65% of patients with iodine-negative metastases have disease that is generally limited to the neck and/or mediastinum. For such patients, the only effective treatment is radical surgery, which can result in a complete, sustained remission in up to 50% of cases [451], confirming the importance of early recognition of recurrent differentiated thyroid cancer, as well as the importance of assessing whether it is limited to one or more organ systems. In this regard, radioguided surgery is a potentially powerful mechanism for managing patients with iodine-negative metastases in whom a more aggressive treatment strategy is required.

##### Radioguided surgery of iodine-avid recurrent differentiated thyroid cancer and recurrent medullary thyroid cancer with iodine radionuclides

The experience of treating differentiated thyroid cancers with ^131^I remnant ablative therapy following total thyroidectomy has made ^131^I the most logical radionuclide for localization of iodine-avid recurrent tumors during radioguided surgery. Due to the fact that ^131^I has a relatively long physical half-life (approximately 8 days), as well as the fact that it is readily available, highly affordable [26], and can be used to distinguish tumor from that of background with a collimated gamma detection probe, it is an ideal radionuclide for detecting iodine-avid recurrent disease. This approach for using ^131^I is well reported in the literature. The first description of radioguided surgery for thyroid cancer was published in 1956 by Harris et al [3] and was later refined in 1971 by Morris et al [452], in which they described using a CsI [Tl] gamma detection probe to localize thyroid tissue or recurrent thyroid cancer in patients undergoing neck exploration.

Recently, Rubello et al [446] reported an eight-day protocol for radioguided surgery for iodine-avid recurrent disease, which was modified from the first original protocol reported by Travagli et al [453]. In this protocol, a therapeutic dosage of 100 mCi (3700 MBq) of ^131^I was orally administered to hypothyroid patients (serum thyroid stimulating hormone level > 30 μU/ml) on day 0, with a whole-body gamma camera imaging (^131^I scan) performed on day 3. Then on day 5, radioguided neck surgery was performed with the assistance of a 15-mm collimated handheld gamma detection probe, and all sites of elevated activity were resected. A subsequent postoperative neck ^131^I scan was obtained on day 7 to evaluate the success of the surgery, utilizing the remaining radioactivity from the initial oral dosage of ^131^I given on day 0. Of the 184 metastatic foci found within the 31 treated patients, 41.3% of extirpated metastatic foci were localized only with the gamma detection probe and were not seen with preoperative imaging. The postoperative neck ^131^I scan on day 7 showed a negative pattern in 25 of 31 treated patients (80.6%). As such, the remaining 6 treated patients showed reduced ^131^I uptake, suggesting persistent iodine-avid residual disease. Alternative ^131^I dosage regimens and different timing regimens for radioguided surgery have been reported. Negele et al [447] reported using a diagnostic dosage of ^131^I in the range of 1.9 to 9.5 mCi (70 to 350 MBq) and radioguided neck surgery performed at 6 to 8 days after the original ^131^I dosage. Their utilization of a significantly lower diagnostic dosage of ^131^I, rather than a therapeutic dosage (100 mCi), avoided the need for hospitalization prior to the planned radioguided neck surgery [447]. Furthermore, Scurry et al [454] reported that radioguided neck surgery was possible for up to three weeks after the administration of a therapeutic dosage of ^131^I.

In an effort to shorten the time interval between radionuclide administration and the time to radioguided surgery for iodine-avid recurrent thyroid cancer, Gallowitsch et al [455] reported using ^123^I instead of ^131^I for radioguided surgery for recurrent papillary thyroid cancer using an oral ^123^I dosage of 2 mCi (74 MBq). The short physical half-life of approximately 13 hours for ^123^I allows it to be administered on the day of or on the day prior to the planned radioguided surgical procedure for an iodine-avid recurrent thyroid cancer [455,456]. Unlike ^131^I, ^123^I is a pure gamma photon emitter, is not associated with the potential stunning effect on subsequent radioiodine uptake, and produces less radiation exposure to the patient and the surgeon [456]. This concept of using ^123^I has also been similarly applied to recurrent medullary thyroid cancer by Shimotake at al [457] in which they reported intravenously administering ^123^I-MIBG at a dosage of 2.7 mCi (100 MBq) at a time 24 hours prior to the planned radioguided surgical procedure. Despite these potentially favorable reasons for utilizing ^123^I instead of ^131^I, the greater expense and relatively limited availability of ^123^I has contributed to the more popular continued use of ^131^I for radioguided surgery for iodine-avid recurrent differentiated thyroid cancers.

##### Radioguided surgery of iodine-negative recurrent differentiated thyroid cancer and recurrent medullary thyroid cancer with ^99m^Tc-labeled radiopharmaceutical agents, ^111^In-labeled radiopharmaceutical agents, and ^18^F-FDG

In contrast, radioguided surgery of iodine-negative recurrent differentiated thyroid cancer and recurrent medullary thyroid cancer has focused primarily upon the use of ^99m^Tc-labeled radiopharmaceutical agents, ^111^In-labeled radiopharmaceutical agents, and ^18^F-FDG. While ^99m^Tc-MIBI [458], ^99m^Tc-dimercaptosuccinic acid (^99m^Tc(V)-DMSA) [459], and ^99m^Tc-tetrafosmin [460] have all been utilized for diagnostic gamma camera imaging of iodine-negative recurrent differentiated thyroid cancers, only ^99m^Tc-MIBI and ^99m^Tc(V)-DMSA have been described for gamma detection probe-directed radioguided surgery.

Rubello et al [461] reported on 37 patients who underwent a previous total thyroidectomy and subsequent ^131^I ablative therapy for differentiated thyroid cancer and who later demonstrated evidence of an iodine-negative recurrence. Each such patient had an elevated thyroglobulin level, a negative ^131^I scan, demonstration of recurrent locoregional recurrent disease on a preoperative ^99m^Tc-MIBI scan and on a high-resolution ultrasound, and no demonstration of distant metastatic disease [461,462]. Then, each patient was taken to the operating suite and, 10 minutes prior to starting the procedure, was intravenously injected with 1 mCi (37 MBq) of ^99m^Tc-MIBI. A gamma detection probe was then used to guide resection of all foci of ^99m^Tc-MIBI uptake. In total, 66 discrete nodules in 37 patients were identified by both high-resolution ultrasound and intraoperative gamma probe detection and were subsequently successfully resected [461]. At follow-up ranging from 9 to 57 months, 27 of 37 patients remained disease-free. In contrast to other approaches, the use of ^99m^Tc-MIBI and intraoperative ultrasound for radioguided surgery of recurrent thyroid cancers is relatively far less expensive and far more available to most medical facilities.

Just as ^99m^Tc-MIBI has proven benefit for the management of recurrent differentiated thyroid cancer, ^99m^Tc(V)-DMSA has been shown to be effective in the diagnostic evaluation of recurrent medullary thyroid cancer, with a detection sensitivity of 95% [459]. Adams et al [463] reported on medullary thyroid cancer recurrences in 25 patients evaluated by preoperative diagnostic tumor localization imaging by computed tomography, ^111^In-DTPA-D-Phe^1^-octreotide imaging, and ^99m^Tc-(V)-DMSA imaging, as well as by intraoperative surgical palpation and by radioguided surgery using an intraoperative gamma probe detection system. All patients were preoperatively injected with an intravenous dose of 6 mCi (222 MBq) of ^111^In-DTPA-D-Phe^1^-octreotide and an intravenous dose of 13.5 mCi (500 MBq) of ^99m^Tc-(V)-DMSA [463]. ^111^In-DTPA-D-Phe^1^-octreotide imaging was performed 4 hours and 24 hours after injection. ^99m^Tc-(V)-DMSA imaging was performed 6 hours after injection. Radioguided surgery was performed approximately 24 hours after ^111^In-DTPA-D-Phe^1^-octreotide injection and approximately 6 hours after ^99m^Tc-(V)-DMSA injection. They demonstrated lesion detection sensitivities of 32%, 34%, and 65% for preoperative diagnostic tumor localization imaging by computed tomography, ^111^In-DTPA-D-Phe^1^-octreotide imaging, and ^99m^Tc-(V)-DMSA imaging, respectively [463]. In addition, lesion detection sensitivities were 65% by intraoperative surgical palpation and 97% by radioguided surgery using a combination of channel selection for both ^111^In and ^99m^Tc on the gamma probe detection unit. In this series, radioguided surgery detected metastases as small as 5 mm in greatest dimension, whereas intraoperative surgical palpation detected metastases only greater than or equal to 1 cm in greatest dimension. Likewise, radioguided surgery was able to identify more than 30% more foci of recurrent medullary thyroid carcinoma compared with conventional preoperative diagnostic tumor localization imaging and intraoperative surgical palpation. Despite these highly encouraging results with recurrent medullary thyroid cancer, ^99m^Tc(V)-DMSA is no longer commercially available for use.

As mentioned above, ^111^In-DTPA-D-Phe^1^-octreotide has been investigated in radioguided surgery for recurrent medullary thyroid cancer [463]. However, the overall sensitivity of detecting metastases from recurrent medullary thyroid cancer on diagnostic ^111^In-DTPA-D-Phe^1^-octreotide imaging [463] is far less than the overall sensitivity of detecting metastases from iodine-negative recurrent differentiated thyroid cancer on diagnostic ^111^In-DTPA-D-Phe^1^-octreotide imaging (34% versus 74%, respectively) [464]. Despite the better overall sensitivity of detecting metastases from iodine-negative recurrent differentiated thyroid cancer on diagnostic ^111^In-DTPA-D-Phe^1^-octreotide imaging, there is no published data on the use of ^111^In-DTPA-D-Phe^1^-octreotide imaging for radioguided surgery for iodine-negative recurrent differentiated thyroid cancer.

^111^In RIGS has been limitedly investigated for recurrent medullary thyroid cancer in France [465-467]. These studies have used a two-step radioimmunotargeting system, consisting of a bispecific antibody (composed of a fragment of anti-CEA monoclonal IgG1 that is coupled chemically with a fragment of anti-DTPA monoclonal IgG1) and an ^111^In-labeled bivalent hapten (^111^In-di-DTPA-tyrosyl-lysine). Patients were first intravenously injected with 0.1 mg/kg of body weight of the bispecific antibody. Approximately three to five day later, patients were then injected with 2.7 mCi to 10 mCi (100 MBq to 370 MBq) of the ^111^In-labeled bivalent hapten. RIGS was then performed approximately two to four days later. Using this RIGS protocol for detecting recurrent and metastatic medullary thyroid cancer, de Labriolle-Vaylet et al [467] most recently reported an accuracy of 86%, a sensitivity of 75%, and a specificity of 90% and suggested its value in the surgical management of this disease process.

The use of ^18^F-FDG for gamma probe-directed radioguided surgery of iodine-negative recurrent thyroid tumors has been recently investigated in a limited fashion [42,45,47,468-470]. It is well-established that ^18^F-FDG PET imaging is particularly useful in the detection of iodine-negative recurrent differentiated thyroid cancer with reported sensitivities ranging from 85% to 95% [471,472] and a specificity as high as 90% [472].

In 2005, Kraeber-Bodéré et al [42] first reported on the use of ^18^F-FDG-directed radioguided surgery in 10 patients with iodine-negative recurrent differentiated thyroid cancer. All 10 patients had previously undergone a diagnostic preoperative ^18^F-FDG PET/CT scan demonstrating abnormal hypermetabolic activity suggesting cervical recurrence, but with no evidence of distant disease. Then, on the day of surgery, at approximately 30 minutes prior to the start of the surgical procedure, patients were injected with a mean dose of 7.2 mCi (265 MBq) of ^18^F-FDG and a subsequent intraoperative radioguided survey of the operative field was performed with a handheld gamma detection probe. Likewise, a completion lymph node dissection was performed in all patients after the intraoperative radioguided survey. A total of 12 abnormal findings were demonstrated on both ^18^F-FDG PET/CT and on the intraoperative radioguided survey, with no additional abnormal finding demonstrated on the intraoperative radioguided survey with the gamma detection probe. Based on the results of the completion lymph node dissection that were performed on all of the patients, they demonstrated that diagnostic preoperative ^18^F-FDG PET/CT scan and intraoperative radioguided survey failed to identify all sites of additional lymph node microscopic metastases in five of the patients.

More recently, Gulec et al [45] reported on four cases of iodine-negative recurrent thyroid cancer undergoing radioguided surgery among a series of 25 patients with various malignancies [45]. In this series, patients were injected with an intravenous dose of 5 to 15 mCi (185 to 555 MBq) of ^18^F-FDG at approximately 2 to 6 hours prior to the planned surgical procedure. Subsequent radioguided surgery was performed with successful localization of nonpalpable metastatic lymph nodes and other target tissues.

Most recently, Agrawal et al [470] reported on two cases of iodine-negative recurrent thyroid cancer at The Ohio State University in which a multimodality approach of perioperative ^18^F-FDG PET/CT imaging (preoperative patient imaging, specimen imaging, and postoperative patient imaging), intraoperative gamma probe detection, and intraoperative ultrasound were utilized for tumor localization and resection of all sites of hypermetabolic activity. The two patients were intravenously injected with 15 to 20 mCi (555 to 740 MBq) of ^18^F-FDG at approximately 120 minutes prior to the planned time of surgery and this multimodality approach allowed for successful identification and resection of all occult sites of iodine-negative recurrent thyroid cancer.

##### Radioguided SLN biopsy

The utility of SLN biopsy for differentiated thyroid cancer remains a topic of major debate. Although it is reported that up to 80% of differentiated thyroid cancer patients have neck lymph node metastases when a lymphadenectomy accompanies the total thyroidectomy [473], only a minority (3% to 30%) of patients will later develop recurrent neck disease [473,474]. Furthermore, in an extensive literature review, Grebe and Hay [474] concluded that nodal disease at presentation was not a predictor of patient survival for papillary thyroid cancer. As a result, the current management of the central neck compartment lymph nodes consists of intraoperatively identifying suspicious lymph nodes, followed by central compartment and superior mediastinal nodal clearance [475].

In the current literature, only a limited number of studies have been published on the feasibility of radioguided SLN biopsy for thyroid cancer [443,476-482]. Those available studies report success of localization in the range of 78% to 100%. In those studies, 0.11 mCi to 3.2 mCi (4 MBq to 120 MBq) of ^99m^Tc colloidal human albumin was injected intratumorally in a total volume of 0.1 mL to 0.5 mL of normal saline. Preoperative lymphoscintigraphy was generally performed. The hemithyroidectomy or total thyroidectomy was generally performed first, followed by the radioguided SLN biopsy procedure, in order to minimize the "shine through" effect which can be especially problematic in the central neck compartment where the lymph nodes may be otherwise within close proximity to the thyroid gland [476]. Despite these encouraging results, it still remains unclear as to whether radioguided SLN biopsy will prove to be of any prognostic value in the management of differentiated thyroid cancer.

#### Parotid gland cancer

Although the generalized concept of a so-called "sentinel node" was first coined by Ernest Gould in 1960 in parotid gland cancer [483], only one report exists within the literature on radioguided SLN biopsy for parotid gland cancer [484], and this single report also represents the only paper addressing any aspect of radioguided surgery for this disease entity. In 2006, Stárek et al [484] reported on six patients injected in eight divided portions into the parotid gland equidistantly from the tumor with approximately 1.35 mCi (50 MBq) of ^99m^Tc colloidal human albumin in a total volume of 2 mL. These patients subsequently underwent preoperative lymphoscintigraphy, radioguided SLN biopsy, and elective lymph node dissection. A SLN was detected in all six cases. In two cases, preoperative lymphoscintigraphy and radioguided SLN biopsy revealed additional sentinel lymph nodes outside the usual extent of elective lymph node dissection that is generally performed for parotid gland cancer. One false-negative (i.e., a positive SLN outside of the parotid gland) was seen with demonstration of intraparotid localization only and was explained by obstruction of the lymphatic outflow from the parotid gland by an intraparotid SLN metastasis.

### Gynecologic malignancies

#### Vulvar cancer

The application of the gamma detection probe in radioguided surgery for vulvar cancer has been limited to radioguided SLN biopsy. Since its first description by Decesare et al in 1997 [485], the feasibility of radioguided SLN biopsy for vulvar cancer has been well evaluated and reported in the literature [268,269,485-509]. In general, ^99m^Tc sulfur colloid or ^99m^Tc colloidal human albumin have been utilized and injected into two to four intradermal sites around the primary tumor in a total dosage range from 0.4 mCi (15 MBq) to 4.0 mCi (150 MBq). In the six most recently published series, localization has been reported in 93% (n = 40) by Nyberg et al [501], 98% (n = 62) by Vidal-Sicart et al [502], 95% (n = 41) by Hauspy et al [503], 100% (n = 43) by Rob et al [504], 95% (n = 39) by Johann et al [508], and 98% (n = 127) by Hampl et al [509], with false negative results reported in 0%, 0%, 0%, 0%, 2.2%, and 7.7% of patients, respectively. Bilateral groin localization has been reported in 19% to 39% [493,500,502], emphasizing the importance of preoperative lymphoscintigraphy for most effectively performing radioguided SLN biopsy in vulvar cancer. Currently, there is a multi-institutional clinical trial (GOG173) for SLN biopsy for vulvar cancer from the collaborative Gynecologic Oncology Group, starting in December 1999 and as of September 2007, has enrolled 456 patients from 40 sites [510]. Since 2001, radioguided SLN biopsy became a mandatory component for enrollment into GOG173 and 316 such patients have been enrolled with 96% localization accomplished using the combined radiocolloid and vital blue dye technique. However, a full disclosure of the results of GOG173 is still pending completion of patient enrollment and publication of the data.

#### Vaginal carcinoma

Radioguided surgery using the gamma detection probe for vaginal carcinoma has been limited to three small case report series addressing radioguided SLN biopsy [490,511,512]. In the largest such radioguided SLN biopsy series reported in 2004 by van Dam et al [511], four patients with vaginal carcinoma received a dose of approximately 1.6 mCi (60 MBq) of ^99m^Tc colloidal human albumin injected into the normal vaginal mucosa (at a depth of 4 to 5 mm) in four locations around the vaginal tumor. Preoperative lymphoscintigraphy was performed and patients with verified localization were subsequently taken to the operating room for laparoscopic radioguided sentinel lymph node biopsy approximately three to five hours after the initial ^99m^Tc colloidal human albumin injection. Localization on preoperative lymphoscintigraphy was demonstrated in three of four (75%) cases. Laparoscopic radioguided SLN biopsy was successful in all three vaginal carcinomas showing localization on preoperative lymphoscintigraphy.

#### Cervical cancer

The application of the gamma detection probe in radioguided surgery for cervical cancer has been limited to radioguided SLN. Since its first description by Verheijen et al [513] and Kamprath et al [514] in 2000, the feasibility of radioguided SLN biopsy for cervical cancer has been well reported in the literature [505-507,515-550]. ^99m^Tc colloidal human albumin, ^99m^Tc sulfur colloid, and ^99m^Tc nanocolloid have all been used as the radiocolloid. The range of time for injection of radiocolloid varies from intraoperative, to immediately preoperative, and to as long as 24 hours prior to the planned surgical procedure. Most commonly, a total dose of the radiocolloid, ranging from 0.5 mCi (18.5 MBq) to 3.2 mCi (120 MBq), has been recommended for injection into the four quadrants of the cervix, while attempting to avoid direct intratumoral injection.

In most series, a sentinel lymph node was generally identified in anywhere from 80% to 100% of patients, a SLN being positive for metastatic disease in 8% to 30% of cases, and false-negative rates were generally reported as approximately 8% with ranges of 0–20% [505-507,515-550]. The finding of bilateral sentinel lymph nodes is generally described in approximately half of the patients, and in as high as 70% of patients in some series. Interestingly, two of the most recently reported series have demonstrated an improved detection rate in primary tumors that are ≤ 2 cm [549,550].

The Arbeitsgemeinschaft Gynäkologische Onkologie (AGO, German Association of Gynecologic Oncologist) has recently published the largest series to date on radioguided SLN in cervical cancer [549]. In this multicenter prospective validation series, patients generally received 1.6 mCi (60 MBq) of ^99m^Tc nanocolloid injected into four sites beneath intact-appearing cervical epithelium on the day before surgery and 4 mL of blue dye injected intraoperatively after induction of anesthesia in a similar manner. Data from a total of 590 patients were used for analysis of the detection rate and data from a total of 507 patients were used for analysis of the diagnostic accuracy. The overall detection rate of pelvic SLNs was 88.6%. The overall sensitivity was only 77.4%, and this was significantly less (P < 0.001) than the predefined acceptability mark of ≥ 90%. Nevertheless, the sensitivity in women with tumors ≤ 20 mm was 90.9%, and bilateral detection was noted in 87.2% of such patients. The overall negative predictive value was 94.3% and negative predictive value was significantly higher (P < 0.001) in patients with tumors ≤ 20 mm (99.1%) as compared with patients with tumors > 20 mm (88.5%) The authors concluded that due to the suboptimal overall sensitivity of 77.4% that complete pelvic lymphadenectomy cannot yet be omitted at this time in the surgical management of cervical cancer. Nevertheless, they commented that SLN biopsy should continue to be investigated in future studies in patients with tumors ≤ 2 cm [549].

Finally, the Gynecologic Oncology Group (GOG) has completed a prospective trial (GOG 206) to determine the false negative rate in a large randomized prospective trial in cervical cancer patients. However, their results have not yet been officially reported. [507,551,552]. In this regard, multiple authors have continued to advocate ongoing prospective evaluation of SLN biopsy before its routine use in the surgical management of cervical cancer can be more universally advocated [505-507,551-554].

#### Endometrial cancer

The application of the gamma detection probe in radioguided surgery for endometrial cancer has been limited to radioguided SLN biopsy. Since its first description by Pelosi et al in 2002 [555], the feasibility of radioguided SLN biopsy for endometrial cancer has been well reported in the literature [505-507,556-574]. Most frequently, ^99m^Tc colloidal human albumin has been used as the radiocolloid. However, ^99m^Tc sulfur colloid and ^99m^Tc phytate have also been utilized. The range of time for injection of radiocolloid varies from intraoperative, to immediately preoperative, and to as long as 24 hours prior to the planned surgical procedure.

Two major injection techniques have been described for the injection of the radiocolloid for endometrial cancer. This includes injection into the uterine corpus and injection into the cervix. Those authors who injected into the cervix [555-557,560,562,564,569-572] usually injected a total dose of the radiocolloid ranging from 1.0 mCi (37 MBq) to 3.24 mCi (120 MBq) into the four quadrants of the cervix to a depth from 0.5 cm to 1.5 cm on the day prior to the surgical procedure. Those authors who injected into the uterine corpus usually inject a total dose of radiocolloid ranging from 0.8 mCi (30 MBq) to 3.0 mCi (111 MBq) into the corpus of the uterus using several different schedules and techniques of injection [558,559,561,563,566-568,572]. Most commonly this is accomplished hysteroscopically by injecting the radiocolloid in a subendometrial fashion in juxtaposition to the tumor at approximately 2 to 6 hours prior to proceeding to the operating room for the staging procedure [561,563,572]. Variations of this technique include those by Nikura et al [559,568] in which they inject radiocolloid hysteroscopically into the endometrium on the day before the surgical procedure and by Delaloye [567] in which they inject radiocolloid hysteroscopically into the endometrium in the operating room immediately prior to starting the staging procedure. Finally, one group [566] attempted to inject into the subserosal myometrium of the uterine fundus at three midline sites at the time of the staging procedure, but achieved only limited success.

In most series, a SLN was generally identified in anywhere from 70% to 100% of patients, a SLN being positive for metastatic disease in 14% to 25% of cases, and false-negative rates were generally reported as 5% or less [505-507,555-574].

It is important to note, that only one prospective randomized clinical trial exists in the literature for endometrial cancer [572] which compared the cervical injection approach (n = 23) to the hysteroscopic subendometrial injection approach (n = 17) for radiocolloid. There was no difference in the SLN detection rates for the cervical injection technique (70%) versus the hysteroscopic subendometrial injection technique (65%). Likewise, no false-negative results were seen in either group. Interestingly, this study showed that there were no identifiable aortic SLNs in the cervical injection group as compared to finding aortic SLNs in 11.8% of patients receiving a hysteroscopic subendometrial injection.

#### Ovarian Cancer

Although radioguided surgery is not routinely utilized in the current surgical management of ovarian cancer, the application of the gamma detection probe for ovarian cancer has been limitedly applied to the radioguided detection of occult primary disease and recurrent disease. No data are available on radioguided SLN biopsy for ovarian cancer. However, RIGS [149,575-582] and ^18^F-FDG-directed surgery [45,53,583] applications of the gamma detection probe have been investigated.

##### RIGS

Since first described for ovarian cancer by Martin et al in 1988 [149,578], the feasibility of RIGS has been evaluated by several groups of investigators [149,575-582]. The focus of all these RIGS protocols has been the detection of occult recurrence in those ovarian cancer patients undergoing both second look laparotomy and those undergoing surgery for suspected or confirmed recurrence. Martin et al [149,578] evaluated ^125^I-B72.3 murine monoclonal antibody, McIntosh et al [582] evaluated ^125^I-CC49 murine monoclonal antibody, and Krag et al [580] evaluated ^111^I-B72.3 murine monoclonal antibody. Gitsch et al [575-577] and Jäger et al [579] evaluated ^131^I-OC125 (anti-CA125) murine monoclonal antibody. Finally, Ind et al [581] evaluated ^99m^Tc labeled murine monoclonal antibodies SM3 and H17E2. Each of these RIGS protocols utilized similar regimens with intravenous injection of 2 to 5 mCi (74 to 185 MBq) of the radiolabeled monoclonal antibody at an interval of 4 to 42 days prior to the planned surgical procedure [149,575-582]. The reported detection rates for RIGS in these reports ranged from 50% to 80%. Despite these early promising results, no further investigations into RIGS for ovarian cancer have been made in over the last 10 years.

##### ^18^F-FDG-directed surgery

Most recently, ^18^F-FDG-directed surgery has been evaluated in the setting of recurrent ovarian cancer [45,53,583]. In 2005, Barranger et al [583] first described ^18^F-FDG-directed surgery in a single patient case report for a patient undergoing laparoscopic excision of recurrent ovarian cancer using a ^18^F-FDG dose of 0.11 mCi/kg (4 MBq/kg). The most recent and largest report to date is by Cohn et al [53] at The Ohio State University. Three patients undergoing laparotomy for recurrent ovarian cancer underwent preoperative intravenous injection of approximately 10 to 20 mCi (370 to 740 MBq) of ^18^F-FDG at approximately 60 to 120 minutes prior to the planned surgical procedure. A combined approach of intraoperative gamma probe detection, specimen PET/CT imaging, and postoperative PET/CT patient imaging was utilized, and demonstrated the feasibility of this approach for establishing the location and extent of disease and confirming complete cytoreduction in selected cases of recurrent ovarian cancer.

### Urologic malignancies

#### Penile cancer

The application of the gamma detection probe in radioguided surgery for penile cancer has been limited to radioguided SLN biopsy. Nevertheless, the routine use of radioguided SLN biopsy for penile cancer remains controversial and is yet to be widely adopted [584].

The generalized concept of a so-called "sentinel node" in our modern medical literature was first coined by Ernest Gould in 1960 in parotid gland cancer [483] and was then later most notably attributed to the work of Ramon Cabañas in 1977 in the area of penile squamous cell carcinoma [585], which was based upon Ramon Cabañas' careful work and meticulous description of lymphadenography of the dorsal lymphatics of the penis [586]. Gould's [483] and Cabañas' [585] strict definition of the "sentinel node" was based on its proximity to a static anatomical landmark (i.e., the angle of the confluence of the anterior and posterior facial veins and the superficial epigastric vein, respectively) and was not based on actual variations in the pattern of lymphatic drainage. In penile cancer, based upon utilizing the superficial epigastric vein as a static anatomical landmark for identifying the so-called "sentinel node" of penile cancer, multiple groups reported variable results and very high false-negative rates [587-591]. Resultantly, controversy has continued about whether the concept of SLN biopsy is reliable in the management of the regional lymph nodes in penile cancer.

It was not until 1993, after Krag et al [135] described the utilization of radioguided SLN biopsy in breast cancer, that a re-emerging interest developed in the concept of SLN biopsy for penile cancer. Since 2000, multiple reports have been published evaluating the feasibility and efficacy of radioguided SLN biopsy for penile cancer [592-609]. In general, ^99m^Tc colloidal human albumin or ^99m^Tc sulfur colloid and have been utilized and injected into one to four intradermal or subdermal sites around the primary tumor in a total dosage range from 0.5 mCi (19 MBq) to 3.2 mCi (120 MBq).

In the four largest published series on penile cancer in which a subsequent formal lymph node dissection was performed only if a SLN was positive, radioguided localization was reported in 97% (n = 74) by Valdés Olmos et al [594], 98% (n = 90) by Tanis et al [597], 98% by Kroon et al [599], and 96% (n = 75) by Hadway et al [607]. Bilateral groin localization was reported in 81%, 79%, 80%, and 91%, respectively [594,597,599,607], emphasizing the extreme importance of preoperative lymphoscintigraphy for most effectively performing accurate radioguided SLN biopsy in penile cancer. A SLN was positive in 22%, 20%, 21%, and 25% of patients, respectively [594,597,599,607]. In this regard, at a median follow-up of 28 months, 7 months, and 11 months, respectively, only 3.6%, 6.3%, and 1.9% of patients with an initial negative SLN biopsy developed a nodal metastasis in a previously SLN-negative nodal basin [594,599,607]. Despite these encouraging results, the routine use of radioguided SLN biopsy for penile cancer remains controversial within the field of urological surgery and may not become widely adopted until there is verification of this technology by a large prospective randomized clinical trial [584].

#### Prostate cancer

The gamma detection probe is not routinely used in the current surgical management of prostate cancer. Nevertheless, radioguided SLN biopsy has been thoroughly evaluated for its potential application in the surgical management of prostate cancer and RIGS has been previously very limitedly investigated.

##### Radioguided SLN biopsy

The utilization of radioguided SLN biopsy for prostate cancer was first reported in 1999 by Wawroschek et al [610]. Since that time, multiple reports have been published on the potential application of radioguided SLN biopsy in the surgical management of prostate cancer [611-632]. Most frequently, ^99m^Tc colloidal human albumin has been used. However, ^99m^Tc phytate, ^99m^Tc sulfur colloid, and ^99m^Tc rhenium sulfur colloid have also been utilized. Patients undergo the radiocolloid injection at an interval from 6 hours to 24 hours before the planned surgery by an ultrasound-guided transrectal approach and into one to three sites within the peripheral zone of the prostate gland. Total dosage range for the radiocolloid is from 1.6 mCi (60 MBq) to 10.8 mCi (400 MBq), which is injected in a total volume of normal saline from 0.2 mL to 3.0 mL. In most series, a SLN was generally identified in 90% to 100% of patients, a SLN was positive in 12% to 25% of cases, and false negative rates were generally reported as 5% or less.

The largest radioguided SLN biopsy series reported to date was recently published by Weckermann and associates from Augsburg, Germany [627]. In their report, they describe 1,055 patients with clinically organ confined prostate cancer undergoing radioguided SLN biopsy, pelvic lymph node dissection, and radical retropubic prostatectomy. A SLN was detected in all patients. A positive SLN were found in 205 patients (19.4%), and only two false negative cases (< 1%) were encountered. One to three positive lymph nodes were found in 95, 50, and 21 cases, respectively, and more than 3 positive lymph nodes were found in 41 cases. Approximately 63% of patients having positive lymph nodes had such positive lymph nodes found outside the region of the standard pelvic lymphadenectomy field. Therefore, Weckermann et al [627] strongly recommended that patients with a positive SLN should undergo extended pelvic lymphadenectomy rather than standard pelvic lymphadenectomy since, in such cases, tumor cells were also additionally detected in non-SLNs.

Finally, several series have reported upon the feasibility of laparoscopic radioguided SLN biopsy [622,624,629,632]. Such a minimally-invasive diagnostic approach to the lymph nodes in the management of prostate cancer may ultimately prove valuable in the evaluation of the lymph node status in patients with an intermediate or high risk of nodal metastases who are either being considered for external beam radiation therapy or for robotic prostatectomy. However, no prospective randomized data are available at this time to more fully address this question.

##### RIGS

The investigation of RIGS in prostate cancer has been limited to a single report on ^125^I-B72.3 monoclonal antibody and a single report on ^111^In-capromab pendetide. In 1993, Badalament et al [633] from The Ohio State University reported their RIGS feasibility study from ten patients with prostate cancer who were planned for radical retropubic prostatectomy and bilateral pelvic lymphadenectomy and that were intravenously injected with 2 mCi (74 MBq) of ^125^I-B72.3 monoclonal antibody at an interval from 17 to 38 days prior to the anticipated date of surgery. RIGS successfully localized tumor in all ten patients, identified otherwise occult bilateral intraprostatic tumor in three patients, correctly identified regional lymph node metastases in two patients, and demonstrated no false-negative results. In 2000, Anderson et al [634] reported a single case of suspected metastatic prostate cancer in which the patient was intravenously injected with 5 mCi (185 MBq) of ^111^In-capromab pendetide. Four days later, the patient underwent RIGS with surgical excision of a RIGS-positive lymph node in the left supraclavicular region that confirmed the diagnosis of metastatic prostate cancer. Despite these two early reports on RIGS in prostate cancer, no further investigations have occurred since that time.

#### Testicular cancer

The application of the gamma detection probe in radioguided surgery for testicular cancer has been only limitedly investigated in regards to radioguided SLN biopsy and ^18^F-FDG-directed surgery.

##### Radioguided SLN biopsy

To date, only three reports exist in the literature on radioguided SLN biopsy for testicular cancer [635-637]. The technique of laparoscopic radioguided SLN biopsy was first described in 2002 for clinical stage I testicular cancer by Tanis et al [635] and by Ohyama et al [636].

In a series of 5 patients, Tanis et al described the technique of laparoscopic radioguided SLN biopsy in two patients [635]. All patients received an intratesticular injection of 1.4 to 3.7 mCi (52 to 135 MBq) of ^99m^Tc colloidal human albumin in 0.15 to 0.3 ml of normal saline and underwent subsequent lymphoscintigraphy. The last two patients underwent same-day transperitoneal laparoscopic radioguided SLN biopsy, with identification of two radioactive SLNs in one patient (with no nodal metastases identified) and no radioactive SLNs identified in the second patient.

In a series of 15 patients, Ohyama et al [636] described the technique of laparoscopic radioguided SLN biopsy in five patients. The day before surgery, each patient was injected into the testicular tissue around the tumor in the testicular tunica albuginea with 0.2 mCi (7.5 MBq) of ^99m^Tc phytate in 0.2 ml of normal saline and underwent subsequent lymphoscintigraphy. The following day, patients one through ten underwent extraperitoneal laparoscopic retroperitoneal lymph node dissection, patients 11 through 14 underwent extraperitoneal laparoscopic radioguided SLN biopsy (with 11 to 14 SLNs removed in each patient and with no nodal metastases identified), and patient 15 underwent extraperitoneal laparoscopic radioguided SLN biopsy with a subsequent confirmatory extraperitoneal laparoscopic retroperitoneal lymph node dissection (with the finding to two micrometastatic disease in SLNs and no metastatic disease in any non-SLNs). Ohyama et al [636] simply concluded that extraperitoneal laparoscopic radioguided SLN biopsy is technically feasible, but requires further clinical study.

Most recently in 2005, Satoh et al [637] reported the most comprehensive series on radioguided SLN biopsy in 22 patients with clinical stage I testicular cancer. The day before surgery, each patient was injected into the testicular tissue around the tumor in the testicular tunica albuginea with 0.4 mCi (15 MBq) of ^99m^Tc phytate in 0.4 ml of normal saline and underwent subsequent lymphoscintigraphy. The following day, patients underwent extraperitoneal laparoscopic radioguided SLN biopsy with a subsequent confirmatory extraperitoneal laparoscopic retroperitoneal lymph node dissection. Radioactive SLNs were identified in 21 of 22 patients (95%), with one radioactive SLN identified in 15 patients and 2 to 4 radioactive SLN identified in the six other patients. Only two patients (9.5%) had micrometastatic disease in radioactive SLNs. Likewise, none of the 21 patients in which a radioactive SLN was identified had metastatic disease in any non-SLNs harvested at the time of the subsequent confirmatory extraperitoneal laparoscopic retroperitoneal lymph node dissection. Two patients with no metastatic disease in their radioactive SLNs or in their subsequent confirmatory extraperitoneal laparoscopic retroperitoneal lymph node dissection developed lymph node disease in the paraaortic lymph nodes at the level of the renal vein and in the obturator lymph nodes at 10 and 20 months after surgery, respectively. Satoh et al [637] concluded that extraperitoneal laparoscopic radioguided SLN biopsy is technically feasible and has potential value in staging and treatment of patients with early-stage testicular cancer. Nevertheless, the lack of any other subsequent reports in the literature or the mention of radioguided SLN biopsy in the National Comprehensive Cancer Network (NCCN) clinical guidelines for testicular cancer [638] leads one to conclude that this is not yet considered an accepted standard of care.

##### ^18^F-FDG-directed surgery

There is a single reported case in the literature by Gulec et al [43] for using ^18^F-FDG-directed surgery for the identification and resection of a hypermetabolic metastatic periaortic lymph node in a case of seminoma.

#### Bladder cancer

Within the published literature, the application of the gamma detection probe in radioguided surgery for bladder cancer has been limited to only a small number of reports from Sweden on radioguided SLN [639-642]. In all instances, ^99m^Tc colloidal human albumin has been utilized and injected into four peritumoral sites within the detrusor muscle in a total dosage range from 1.4 mCi (50 MBq) to 1.9 mCi (70 MBq). In 2001, Sherif et al [639] were the first to report on 13 bladder cancer patients in which localization was seen in 11 of 13 patients (85%), with four patients having a positive SLN, and with no false-negative results. In 2003 Liedberg et al [640] reported on 26 bladder cancer patients in which localization was seen in 21 of 26 patients (81%), with seven patients having a positive SLN, and with one false-negative result. In 2006, Liedberg et al [641] reported on 75 bladder cancer patients in which localization was seen in 65 of 75 patients (87%), with 26 patients having a positive SLN, and with six false-negative results (19%). Despite the higher false-negative rate reported by Liedberg et al [641], a common theme to all these reports has been that approximately one-quarter of all patients that localized had a SLN that was anatomically located outside the field of the normally excised lymph nodes of the obturator fossa, thus leading to improved overall lymph node staging when radioguided SLN biopsy was used in conjunction with standard lymphadenectomy. Finally, Sherif et al [642] demonstrated that the use of fused single-photon emission computed tomography (SPECT) and computed tomography (CT) preoperative imaging aided in the intraoperative identification of both metastatic and non-metastatic SLNs.

#### Renal cell cancer

Only two reports involving cases of renal cell cancer can be found within the published literature that focus on the use of the gamma detection probe in radioguided surgery [29,643]. Both reports involve RIGS with the utilization of monoclonal antibodies. However, only one of these two reports describes the use of a particular monoclonal antibody that is specific to renal cell carcinoma [29].

Recently, in 2008, Strong et al [29], reported on two cases of clear-cell renal cell cancer in which RIGS was performed using the monoclonal antibody cG250 and ^124^I (Table 1). cG250 is well known to bind with a high specificity to clear-cell renal cell cancers [644-646]. The specific antigen target of cG250 is an epitope of carbonic anhydrase IX [29,646]. In these two RIGS cases in the Strong et al series [29], the patients were each intravenously injected with 5 mCi (187 MBq) of ^124^I-labeled cG250 approximately seven days prior to surgery. PET imaging was performed four hours post-injection of ^124^I-labeled cG250 and then again approximately seven days later at a time specifically within three hours of the start of surgery. Intraoperatively, both a gamma detection probe and a beta detection probe were utilized for tumor identification. No significant difference in the ratio of tumor/background counts were found for the two different types of radiation detection probes utilized.

The incidental finding of metastatic renal cell cancer at the time of RIGS performed for a presumed primary colon cancer was previously reported by Avital et al in 1998 [643]. In this report, a 72 year old female with a remote history of renal cell cancer requiring a right nephrectomy some five years earlier and who was now presumed to have a primary colon cancer underwent RIGS after receiving an intravenous injection of 2 mCi (74 MBq) of ^125^I-labeled murine CC49 monoclonal antibody approximately three weeks prior to surgery. After pathologic evaluation of the case, it was determined that the colonic mass was, in fact, a metastatic renal cell cancer deposit that had metastasized to and through all layers of the colonic wall. Immunohistochemistry revealed that the tumor cells composing the metastatic renal cell cancer were themselves negative for CC49 monoclonal antibody staining. However, the normal colonic mucosa adjacent to the metastatic renal cell cancer demonstrated strong CC49 monoclonal antibody staining. Therefore, in retrospect, the adjacent normal colonic mucosa and not the remotely occurring metastatic renal cell cancer deposit was responsible for the accumulation of the ^125^I-labeled murine CC49 monoclonal antibody detected at the time of RIGS.

### Thoracic malignancies

#### Lung cancer

The use of a gamma detection probe in lung cancer dates back to 1984 when Woolfenden et al [647] first described using a miniature NaI [Tl] detector during fiberoptic bronchoscopy to attempt to detect and localize sites of deposit of ^57^Co bleomycin in 34 patients with suspected lung cancer. Despite the promising results of this report, the extremely long physical half-life of ^57^Co (271.8 days) has made it a highly undesirable radionuclide for any further use in radioguided surgical procedures. Since that time, radioguided surgery for lung cancer has been further investigated using ^99m^Tc for radioguided SLN biopsy, using ^125^I-labeled and ^99m^Tc-labeled monoclonal antibodies for RIGS, as well as using ^99m^Tc-peplomycin and ^18^F-FDG for other radioguided surgical applications.

##### Radioguided SLN biopsy

The utilization of radioguided SLN biopsy for non-small cell lung cancer was first reported in 2000 by Liptay et al [648]. Since that time, multiple reports have been published on the potential application of radioguided SLN biopsy in the surgical management of non-small cell lung cancer [649-668]. ^99m^Tc tin colloid, ^99m^Tc sulfur colloid, and ^99m^Tc colloidal human albumin have all been utilized in radioguided sentinel lymph node biopsy for non-small cell lung cancer. Some authors have advocated preoperative injection of radiocolloid into or around the primary tumor by CT guidance on the day of surgery or on the day before surgery. Other authors have advocated intraoperative injection of radiocolloid into or around the primary tumor after direct visualization of the tumor. While still other authors advocate intraoperative endobronchial injection of radiocolloid into directly visualized endobronchial tumor or transbronchially at the most distal pulmonary sub-segment that could be reached endobronchially within proximity to the primary tumor. Total dosage range for the radiocolloid is from 0.25 mCi (9.25 MBq) to 8 mCi (296 MBq), which is injected in a total volume of normal saline from 0.5 mL to 2.0 mL. In all series reporting more than one hundred patients undergoing radioguided SLN biopsy for non-small cell lung cancer [657,659,662,668], a SLN was generally identified in 70% to 100% of patients and false negative rates were generally reported as 10% or less. Despite these numerous reports and their encouraging results, the routine use of radioguided SLN biopsy in the surgical management of non-small cell lung cancer has not been embraced. Nevertheless, those authors who have played an active role in its evaluation in the surgical management of non-small cell lung cancer remain optimistic about its future applications [669].

##### RIGS

The application of RIGS technology to adenocarcinoma of the lung has been previously investigated and reported in an extremely limited fashion [670,671]. In 1998, Grazia et al [670] intraoperatively evaluated 9 patients with adenocarcinoma of the lung with the gamma detection probe after intravenous injection of 0.9 to 1.5 mCi (33 to 56 MBq) of ^125^I-labeled monoclonal antibody B72.3 at 11 to 44 days (mean of 20.4 days) prior to the surgical procedure. Specific binding to histologically confirmed sites of adenocarcinoma of the lung was noted in all such cases. However, the high radioactive background counts found in the thorax, due to the heart and major vessels, was postulated as a drawback to further investigation of this technique. Likewise, in 1998, Mansi et al [671], evaluated both ^125^I-labeled monoclonal antibody B72.3 and ^99m^Tc-labeled fragments of the murine anti-CEA monoclonal antibody F023C5 in lung cancer patients. In the first phase of their study, using immunohistochemistry, they found binding of monoclonal antibody B72.3 in only 6 of 45 primary non-small cell lung cancers. Only one operable patient was then intravenously injected with 2 mCi (74 MBq) of ^125^I-labeled monoclonal antibody B72.3 and no selectivity for neoplastic cells was seen during RIGS using the gamma detection probe. In the second phase of their study, 11 patients with squamous cell lung cancer were intravenously injected with 15 mCi (555 MBq) of ^99m^Tc-labeled fragments of the murine anti-CEA monoclonal antibody F023C5. Immunoscintography was then performed six to 24 hours thereafter. Only one patient was operable and underwent RIGS in concert with a pneumonectomy and lymphadenectomy at a time some 36 hours after the original intravenous injection of the ^99m^Tc-labeled fragments of the murine anti-CEA monoclonal antibody F023C5. Using the gamma detection probe, *in vivo *detection of the primary tumor was not accomplished; however, resected neoplastic tissue demonstrated a 2:1 tumor-to-background ratio on *ex vivo *counting. Likewise, RIGS identified a small lymph node metastasis that was not previously identified by CT scan or immunoscintigraphy. Despite these two early reports on RIGS in lung cancer, no further investigations have occurred since that time.

##### ^99m^Tc-peplomycin radioguided surgery

In 2003, Wang et al [672] reported on a series of 37 patients with lung neoplasms who were intravenously injected with a non-specified dose of ^99m^Tc-peplomycin. Peplomycin is a semi-synthetic analog of bleomycin. Following injection, patients underwent preoperative scintigraphic imaging for identification of radiotracer uptake by tumor-bearing tissue and were then subsequently taken to the operating room for radioguided surgery using the gamma detection probe. The sensitivity, specificity, and accuracy was reported as 90%, 88%, and 89%, respectively, for identifying malignant lung lesions, and was reported as 91%, 88%, and 90%, respectively, for identifying lymph node metastases.

##### ^18^F-FDG-directed surgery

Much more recently, the application of intraoperative gamma probe detection after a preoperative same-day injection of ^18^F-FDG has been reported in a limited number of selected cases of non-small cell lung cancer [45,673,674]. First reported in 2006, Nwogu et al [673] evaluated 10 patients with non-small cell lung cancer that were intravenously injected with approximately 10 mCi (370 MBq) of ^18^F-FDG on the day of surgery. All resected primary tumors were FDG-avid. With regards to evaluation of the thoracic lymph nodes, there were 5 cases of true-positive FDG-avid thoracic lymph nodes, 3 cases of false positive FDG-avid thoracic lymph nodes, and 2 false-negative cases in which the thoracic lymph nodes were not FDG-avid but were found to contain metastatic disease. In this regard, and of most recent note, Moffatt-Bruce et al at The Ohio State University [674] has recently reported a combined approach for radioguided localization of FDG-avid tissues for a single case of lung cancer using perioperative ^18^F-FDG PET/CT imaging (using a triad of preoperative PET/CT imaging, specimen PET/CT imaging, and postoperative PET/CT imaging) and intraoperative gamma probe detection, in which a dose of approximately 26.1 mCi (966 MBq) ^18^F-FDG was injected intravenously approximately 98 minutes prior to the start of radioguided localization of FDG-avid tissues.

#### Pulmonary nodules

The application of the gamma detection probe for the intraoperative localization and video-assisted thoracoscopic wedge resection of solitary or multiple small indeterminate pulmonary nodules has been investigated [675-683]. Generally, on the morning of surgery, such pulmonary lesions are injected under CT guidance with ^99m^Tc colloidal human albumin in dosage range from 0.14 mCi (5 MBq) to 0.30 mCi (11 MBq) and in a total volume of normal saline from 0.1 mL to 0.3 mL. Subsequently, such patients are taken to the operating room for video-assisted thoracoscopic wedge resection of these previously injected pulmonary nodules. In the largest and most recent study conducted to date, Grogan et al (683) reported that pulmonary nodules were successfully localized and excised by this radioguided technique in 77 of 81 patients (95%), and were exclusively thoracoscopically excised in 71 of 77 patients (92%).

### Neuroendocrine tumors

#### Gastroenteropancreatic (GEP) neuroendocrine tumors

While GEP neuroendocrine tumors generally follow a protracted clinical course, surgery remains integral in the management of localized primary tumors, recurrent disease, or metastatic disease [684-687]. Therefore, the ability to detect and localize GEP neuroendocrine tumor tissue is paramount to the effectiveness of surgical treatment and was revolutionized by Krenning's landmark description of radiolabeled octreotide scintigraphy using ^111^In-DTPA-D-Phe^1^-octreotide and ^123^I-Tyr^3^-octreotide, which both specifically target somatostatin receptors [688]. As a result, ^111^In-DTPA-D-Phe^1^-octreotide imaging has been coupled with single photon emission computerized tomography (SPECT) to become the primary imaging and staging modality of patients with GEP neuroendocrine tumors, exceeding the sensitivity of MRI and CT [689,690]. Despite this well-established preoperative imaging technology, intraoperative localization of such disease has remained a formidable challenge with negative laparotomy rates reported as high as 30% for patients with ileal and pancreatic endocrine tumors [686,691]. The intraoperative application of ultrasound, transduodenal illumination, and duodenotomy has been advocated by some [692] to pinpoint GEP neuroendocrine tumors particularly of the duodenum and pancreatic head region, a location historically known as the "gastrinoma triangle."

Another approach to intraoperative localization of GEP neuroendocrine tumors is use of the gamma detection probe for radioguided surgery. Radiopharmaceutical agents utilized for intraoperative detection of GEP neuroendocrine tumors include ^111^In-DTPA-D-Phe^1^-octreotide [693-695], ^125^I-Tyr^3^-octreotide [696,697], ^123^I-MIBG [690], and ^99m^Tc-EDDA/HYNIC octreotate [698]. However, the use of ^111^In-DTPA-D-Phe^1^-octreotide has become far more prevalent for several reasons. First, the considerably shorter physical half-life of ^111^In (approximately 68 hours) as compared to ^125^I (approximately 60 days) make ^111^In more desirable secondary to the importance of radiation safety issues related to the storage and disposal of radioactive materials [49]. Second, the predominant hepatobiliary excretion of ^125^I-Tyr^3^-octreotide leads to a considerable amount of intestinal activity, reducing the reliability of somatostatin receptor imaging [688]. Third, the intestinal clearance of ^111^In-DTPA-D-Phe^1^-octreotide can be overcome by the administration of laxatives. Lastly, the intraoperative tumor to background ratio is improved with most cases, demonstrating a ratio greater than 4:1 [694]. Despite these relative advantages and disadvantages, ^111^In, ^125^I, ^123^I and ^99m^Tc radiopharmaceutical agents have all been utilized to detect primary, recurrent, and metastatic lesions from multiple GEP neuroendocrine tumors, including gastrinomas, carcinoids, and insulinomas.

The standard technique for radioguided surgery of GEP neuroendocrine tumors involves the intravenous injection of ^111^In-DTPA-D-Phe^1^-octreotide at dosage range of 3.0 to 6.0 mCi (110 to 220 MBq) [684,687,694] with subsequent 4-hour and 24-hour whole body scintigraphy and 24-hour SPECT imaging. Surgical exploration is performed approximately 48 to 72 hours post-injection and is preceded by a bowel preparation to minimize the background radiation from physiologic bowel excretion of ^111^In-DTPA-D-Phe^1^-octreotide. A higher energy gamma detection probe (with a gamma photon energy range at or above the 247 keV photopeak of ^111^In) is required. Once an area of high radioactivity is located, the gamma detection probe is held stationary and 10-second counts are obtained. A tumor to background ratio of at least 1.5 or greater is required for confirmation of the tissue localization [687]. If ^125^I-Tyr^3^-octreotide is utilized instead of ^111^In-DTPA-D-Phe^1^-octreotide, the radiopharmaceutical agent is given the day of the procedure with Lugol's solution (a solution containing 5% iodine [I_2_] and 10% potassium iodide [KI] in 85% distilled water with a total iodine content of 130 mg/mL) administered three days immediately before the planned surgery to prevent excessive thyroidal uptake of unbound ^125^I [697].

As reported by Adams and Baum [684], the use of the gamma detection probe with ^111^In-DTPA-D-Phe^1^-octreotide has increased the intraoperative detection of GEP neuroendocrine tumors, thus identifying 57% more lesions than with conventional surgical palpation. In addition, the intraoperative gamma detection probe was able to identify small tumors (even down to less than 5 mm in size) more efficiently than somatostatin receptor scintigraphy (greater than 90% versus 68% to 77%, respectively) [684]. As a result of this improved detection rate, cytoreductive radioguided surgery for GEP neuroendocrine tumors has resulted in improved long-term outcomes for patients with recurrent disease and/or metastatic disease [685,699]. In recurrent tumor surgery, much of the operative field is obscured with scar tissue prohibiting accurate localization with palpation. Radioguided surgery can distinguish tumor from scar tissue, thereby significantly aiding in the surgical dissection and extirpation of all sites of tumor [695,696]. As a result, radioguided surgery for detection of with ^111^In-DTPA-D-Phe^1^-octreotide with the gamma detection probe has proven to be a useful adjunct in the management of GEP neuroendocrine tumors.

In a recent report by Hubalewska-Dydejczyk et al [698], they describe the use of ^99m^Tc-EDDA/HYNIC octreotate for radioguided surgery in patients with suspected GEP neuroendocrine tumors who had negative results on other preoperative imaging tests. Based on an 18.9 mCi (700 MBq) dose of ^99m^Tc-EDDA/HYNIC octreotate intravenously injected 24 hours prior to surgery, radioguided detection of this new somatostatin analogue with the gamma detection probe was successful in identifying both carcinoid tumors and islet cell tumors within the study group.

Recently, ^68^Ga has been labeled to octreotide by way of DOTA (1,4,7,10-tetraazacyclododecane-1,4,7,10-tetra-acetic acid), a macrocyclic chelator, for the creation of ^68^Ga-DOTA-Tyr^3^-octreotide [687,700] for the preoperative scintigraphic detection of GEP neuroendocrine tumors. The radionuclide ^68^Ga has a physical half-life of approximately 68 minutes and decays by 89% through positron emission of a maximum energy of 1.92 MeV (making it ideal for PET imaging) and by 11% through orbital electron capture. ^68^Ga-DOTA-Tyr^3^-octreotide has recently been described as a potentially highly desirable radiopharmaceutical agent for radioguided surgery for GEP neuroendocrine tumors [687]. Nevertheless, to date, no clinical studies involving actual radioguided surgical procedures for GEP neuroendocrine tumors have been published to substantiate this claim for ^68^Ga-DOTA-Tyr^3^-octreotide.

Most recently, ^18^F-L-dihydroxyphenylalanine (^18^F-L-DOPA) was utilized for combined preoperative PET/CT imaging and radioguided surgery with the gamma detection probe for the detection of multiple site of metastatic GEP neuroendocrine tumor in a single patient with ^111^In-DTPA-octreotide negative disease [701]. However, more time will be needed to thoroughly assess whether this ^18^F-based technology will become clinically relevant in the surgical management of metastatic GEP neuroendocrine tumor.

#### Bronchial carcinoids

Only two case reports exist in the literature describing radioguided surgery with ^111^In pentetreotide for bronchial carcinoids. In 1997 Mansi et al [702] reported on a 28 year old male patient with Cushing's syndrome from an adrenocorticotrophin (ATCH)-secreting bronchial carcinoid who, four days after receiving a 3 mCi (111 MBq) intravenous injection of ^111^In-DTPA-D-Phe^1^-octreotide, underwent radioguided surgery with the assistance of a gamma detection probe and which allowed for accurate localization and resection of a 1.8 cm tumor in the right lower lobe with *in vivo *tumor to normal surrounding tissue count ratios of 6:1 and *ex vivo *tumor to normal surrounding tissue count ratios of 18:1. Similarly, in 2005, Grossrubatscher et al [703] reported on a 28 year old female patient with Cushing's syndrome from an adrenocorticotrophin (ATCH)-secreting bronchial carcinoid who, two days after receiving a 4 mCi (148 MBq) intravenous injection of ^111^In-DTPA-D-Phe^1^-octreotide, underwent radioguided thoracoscopic surgery with the assistance of a gamma detection probe and which allowed for accurate localization and resection of multiple metastatic lymph nodes within the upper mediastinal region.

#### Neuroblastoma

Only very limited data are available on the use of the gamma detection probe during radioguided surgery for neuroblastoma [684,704-707]. This was first reported in 1995 by Martinez et al [704] in six pediatric patients with stage II or IV neuroblastoma using an intravenous injection of approximately 0.03 to 0.2 mCi (1.1 to7.4 MBq) of ^125^I-Tyr^3^-octreotide given 1.8 to 7.5 hours prior to surgery. The gamma detection probe found 17 sites of ^125^I-Tyr^3^-octreotide binding, and of which 15 sites contained neuroblastoma. Five of these 15 sites of neuroblastoma were occult and were not detected by standard intraoperative palpation and visualization. Next, in 1997, Heij et al [705] reported on five pediatric patients with recurrent abdominal neuroblastoma that were intravenously injected with 5 mCi (185 MBq) of ^123^I-MIBG that was given 48 hours prior to surgery. The gamma detection probe correctly identified tumor seen on preoperative MIGB scan in all five patients. Thereafter, in 1998, Martelli et al [706] reported the largest series of 66 radioguided surgical procedures in 58 pediatric patients undergoing intraoperative localization of neuroblastoma with the gamma detection probe and utilizing either ^123^I-MIBG (mean dose of 5 mCi or 185 MBq intravenously injected 24 to 48 hours before surgery) or ^125^I-MIBG (mean dose of 0.11 mCi or 4 MBq intravenously injected three to five days before surgery). A tumor to background ratio that exceeded 2:1 was considered positive for target localization, and in 65% of the cases, the gamma detection probe was considered helpful. The gamma detection probe permitted detection of small, nonpalpable tumors in sites that were difficult to access surgically as well as aided in more accurately defining tumor margins or areas of tumor extension. The sensitivities of the ^123^I-MIBG and ^125^I-MIBG were similar, at 91% and 92% respectively. However, the specificity of ^125^I-MIBG was significantly higher than ^123^I-MIBG (85% as compared to 55%, respectively). Comparable results for ^123^I-MIBG and ^125^I-MIBG in neuroblastoma patients were reported by Adams and Baum [684]. In addition, Adams and Baum [684] attempted intraoperative neuroblastoma localization with ^111^In-DTPA-D-Phe^1^-octreotide. However, they found the urinary excretion of the radionuclide limited the use of the gamma probe in tissues adjacent to the kidneys and ureters.

#### Pheochromocytoma

MIBG scintigraphy, with a sensitivity and specificity as high as 88% and 96%, respectively, is the technique of choice for the localization of pheochromocytoma [708]. Only very limited data is available on intraoperative localization of pheochromocytomas with iodine-based radiopharmaceutical MIBG agents, including ^131^I-MIBG [709], ^125^I-MIBG [710,711], and ^123^I-MIBG [463] and has confirmed the feasibility of this technique. The technique for preoperative administration of the iodine-based radiopharmaceutical MIBG agent varies based on the physical half-life of the agent, with Proye et al [710] reporting intravenously injecting 0.5 mCi to 1.0 mCi (18 MBq to 37 MBq) of ^125^I-MIBG approximately 3 days before surgery with thyroid blockade and with Adams et al [463] reporting intravenously injecting 4.9 mCi (180 MBq) of ^123^I-MIBG approximately 4 to 5 hours before surgery without thyroid blockade. Tumor to background ratios exceeding 5:1 were achieved [710,711]. Detection of tumor foci less than or equal to one centimeter in size was demonstrated [711]. However, Adams et al [463] suggested that avid hepatic and biliary signals may overshadow potential intraoperative sites of pathologic MIGB uptake seen on whole body scanning, especially in the subdiaphragmatic/paravertebral region, and may lead to low tumor to background ratios of 1.5:1.0. As a result, only very limited acceptance of this radioguided surgical technique has been given since these early published series.

### Adrenocortical carcinoma

The use of the gamma detection probe in radioguided surgery for adrenocortical carcinoma is virtually nonexistent. Only one report exists in the literature that describes a single case of ^18^F-FDG-directed surgery for adrenocortical carcinoma [45]. In this report, ^18^F-FDG-directed surgery was instrumental in locating difficult to access metastatic lymph nodes at the time of a combined sternotomy and laparatomy.

### Sarcoma

The application of the gamma detection probe for the surgical management of sarcoma has been extremely limited. The vast majority of sarcoma histologic subtypes metastasize only hematologically. Nevertheless, a few sarcoma histologic subtypes have a propensity for lymphatic dissemination, such as rhabdomyosarcoma, epithelioid sarcoma, clear cell sarcoma, and synovial sarcoma [712]. In this regard, radioguided SLN biopsy has been limitedly investigated in some of these select sarcoma histologic subtypes [713-722]. These reports are limited to a few case reports [714,716,717,719] and to a few small series [713,715,718,720-722]. Most frequently, ^99m^Tc colloidal human albumin, ^99m^Tc sulfur colloid, and ^99m^Tc rhenium sulfur colloid have been utilized. However, the timing of injection, site of injection, and total dosage range for the radiocolloid have generally not been well-characterized in these limited reports.

The largest series reported to date was recently published by Kayton et al [721]. In their report, they described 23 pediatric patients with sarcoma (9 rhabdomyosarcomas, 4 epithelioid sarcomas, 2 synovial sarcomas, 2 alveolar soft part sarcomas, 2 fibrosarcomas, 2 clear cell sarcomas, 1 Ewing sarcoma, and 1 angiomatoid fibrous histiocytoma) undergoing radioguided SLN biopsy using a dosage range of 0.05 mCi (1.9 MBq) to 0.8 mCi (29.6 MBq) of ^99m^Tc sulfur colloid that was injected into one to four locations at the site of the primary tumor. A SLN was detected in all patients. A positive SLN was found in only one patient (4.3%). Since the radioguided SLN biopsy was not accompanied by a confirmatory lymph node dissection, a false negative rate could not be determined.

### Brain tumors

Radioguided surgical resection of brain tumors has been investigated in a very limited fashion [723-727]. In 2002, Vilela Filho and Carneiro Filho [723] first described use of ^99m^Tc-MIBI, in a dose of 30 mCi (1110 MBq), for gamma probe-assisted resection of a metastatic renal cell carcinoma to the right parietal lobe. In 2004, Kojima et al [724] reported on the use of ^99m^Tc-MIBI, in an unknown dose, for gamma probe-assisted resection in 13 patients with either primary or recurrent astrocytomas or metastatic brain lesions. In 2005, Gay et al [725] reported on the use of ^111^In-(DTPA)-D-Phe1 pentetreotide, an analog of somatostatin, in a dose of 3 mCi (111 MBq), for gamma probe-assisted resection in 10 patients with *en plaque *meningiomas. In 2006, Serrano et al [726] reported on the use of ^201^Tl, in a dose of 1.4 mCi (50 MBq), for gamma probe-assisted resection of an astrocytoma of the right temporoparietal region. Finally, in 2007, Bhanot et al [727] reported on the use of ^99m^Tc-MIBI, in a dose of 10 mCi (370 MBq), for gamma probe-assisted resection in 13 patients with supratentorial gliomas. However, at this time, other methods of intraoperative neurosurgical navigation, such as with magnetic resonance imaging and ultrasound, which do not utilize radioactive substances, remain the standard of care.

### Bone lesions

The use of the gamma detection probe in radioguided surgery has been extensively described for radioguided biopsy and/or resection of benign bone lesions, such as osteoid osteomas [728-742] and enchondromas [743], as well as suspected bone metastases [743-755]. The application of gamma detection probe technology for radioguided biopsy and/or resection of bone lesions in the early 1980s [728,743] truly represents some of the early forward thinking that contributed to the later development of the modern era of radioguided surgery (Table 1).

The technique radioguided biopsy and/or resection of bone lesions utilized the same class of radiopharmaceutical agents (^99m^Tc diphosphonates) and the same dosing range as is utilized for diagnostic bone scanning. The most common ^99m^Tc diphosphonates utilized are ^99m^Tc MDP, ^99m^Tc HMDP, and ^99m^Tc HDP. These radiopharmaceutical agents are administered by a peripheral intravenous injection in a dosing range of approximately 15 mCi (555 MBq) to 30 mCi (1,110 MBq) at a time approximately 2 to 12 hours prior to the planned surgical procedure. Reasonable success has been reported with guiding diagnostic biopsy of such lesions as well as guiding the surgeon for complete surgical resection of such lesions. Nevertheless, this technique, as it relates to bone lesions, has not become a part of the mainstream clinical practice for the biopsy and/or resection of bone lesions.

### Lymphoma

The application of intraoperative gamma probe-directed biopsy of suspicious lesions for confirming the diagnosis of lymphoma has been very limited [43,45,746,755,756]. Robinson et al [747] described using ^99m^Tc HMDP for intraoperative gamma probe-directed biopsy of two rib lesions representing lymphoma. Schattner et al [756] described using ^67^Ga for intraoperative gamma probe-directed biopsy of increased activity within the left paraspinal and psoas muscles seen on whole body gallium scan and single-photon emission computed tomography (SPECT) for confirming the diagnosis of lymphoma. Burdine et al [757] described using ^99m^Tc sulfur colloid for intraoperative gamma probe-directed biopsy of small, nonpalpable, non-visible, suspicious pulmonary nodules during video-assisted thoracoscopic surgery in two patients with a history of lymphoma. Most recently, Gulec et al [43,45] have described using ^18^F-FDG for intraoperative gamma probe-directed biopsy of nonpalpable suspicious lymph nodes seen on diagnostic whole body PET scan for confirming the diagnosis of lymphoma.

### Radioguided monitoring of isolated limb perfusion

The utility of a gamma detection probe system for intraoperative radioguided monitoring of systemic leakage during isolated limb perfusion surgery for both melanoma and sarcoma has been previously investigated [758-767]. The basic principles of this technique were first described in 1961 by Stehlin et al [758] using a single, large, overhead-mounted scintillation detector that displayed continuous tracing results, in counts per minute, on a rectilinear recorder for detecting ^131^I-labeled human serum albumin. The principles of this technique using a more modern handheld gamma detection probe system within the operating room were later described in 1989 by Sardi et al [759] at The Ohio State University. This system by Sardi et al [759] consisted on two handheld gamma probes, with one positioned over the precordial area and one positioned over the distal aspect of the thigh. Each patient received 0.8 mCi (29.6 MBq) of ^99m^Tc pentetate through the perfusate circulation. The percentage of ^99m^Tc pentetate leakage was calculated by a simultaneous reading of the two gamma detection probes at one-minute intervals. When compared to a method of intermittent simultaneous blood sampling from the perfusate and systemic circulations at 15 minutes intervals, essentially identical percentage of leakage was detected by both methods. However, the minute by minute monitoring of the two handheld gamma probe system allowed for a more instantaneous and real-time indication of any fluctuations in the percentage of leakage than did the intermittent (every 15-minute) blood sampling from the perfusate and systemic circulations.

Since that time, multiple variations of this technique have been described [760-767]. Manner et al [760] have advocated the use of a two-probe system (precordial and thigh) with 0.50 mCi (18.5 MBq) of ^111^In-labeled red blood cells injected into the perfusate circulation. Sprenger et al [761] have advocated the use of a three-probe system (precordial, thigh, and perfusate circuit) with low-dose (i.e., 0.004 mCi or 0.15 MBq) of ^111^In-labeled red blood cells injected into the systemic circulation (to establish a minimum baseline reference activity within the systemic circulation) and a subsequent high-dose (i.e., 0.32 mCi or 12 MBq) of ^111^In-labeled red blood cells injected into the perfusate circulation. Sandrock et al [763] have advocated the use of a precordial one-probe system with ^99m^Tc-labeled red blood cells (0.4 mCi or 15 MBq) injected into the perfusate circulation. Barker et al [762] have advocated the use of a precordial one-probe system with a combination radionuclide design consisting of a low-dose (i.e., 0.02 mCi or 0.74 MBq) of ^131^I-labeled human serum albumin injected into the systemic circulation and a subsequent 10-fold higher dose (i.e., 0.20 mCi or 7.4 MBq) of ^131^I-labeled human serum albumin injected into the perfusate circulation. Similarly, Daryanani et al [764] and Van Ginkel et al [765] have advocated the use of a precordial one-probe system with a combination radionuclide design consisting of a low-dose (i.e., 0.014 mCi or 0.5 MBq) of ^131^I-labeled human serum albumin and 0.3 mCi (10 MBq) of ^99m^Tc-labeled human serum albumin injected into the systemic circulation and a subsequent 10-fold higher dose (i.e., 0.14 mCi or 5 MBq) of ^131^I-labeled human serum albumin injected into the perfusate circulation. Finally, Casara et al [766,767] have advocated a precordial one-probe system with 0.014 mCi/kg (0.5 MBq/kg) of ^99m^Tc-labeled human serum albumin injected into the perfusate circulation at the time of the isolated limb perfusion surgery and in conjunction with prior simulation testing with 0.0014 mCi/kg (0.05 MBq/kg) of ^99m^Tc-labeled human serum albumin injected into the systemic circulation of the patient at a time 48 to 72 hours prior to the isolated limb perfusion surgery. Despite variations in these techniques, all have verified the potential utility of the gamma detection probe for intraoperative radioguided monitoring of systemic leakage during isolated limb perfusion surgery.

## Conclusion

Radioguided surgery using gamma detection probe technology has tremendously expanded and has evolved into what is now considered an established discipline within the practice of surgery. It has been investigated in and applied to almost every imaginable neoplastic disease entity that is surgically managed, and it has revolutionized the surgical management of breast cancer, melanoma, colorectal cancer, and parathyroid disease. The impact of radioguided surgery on the surgical management of cancer patients includes providing vital and real-time information to the surgeon regarding the location and extent of disease, as well as regarding the assessment of surgical resection margins. Additionally, it has allowed the surgeon to minimize the surgical invasiveness of many diagnostic and therapeutic procedures, while still maintaining maximum benefit to the cancer patient. As we move forward through the 21^st ^century, we hope to continue to build upon our current technological platform of radioguided surgery and further integrate advanced medical imaging within the operative arena in order to supplement intraoperative gamma detection probe technology [16,768].

## Competing interests

The authors declare that they have no competing interests.

## Authors' contributions

The initial planning and development of the concept and content of the review manuscript was undertaken by SPP and EWM. The individual sections comprising the review manuscript were written by SPP, RLN, CMM, and DMO. SPP assumed the supervising editorial role for all the authors and was responsible for editing and organizing all the individual sections into the review manuscript. EWM was the senior physician overseeing the entire project. RLN, CMM, DMO, GHH, NCH, DAM, MVK, and EWM all played a critical role and had a substantial contribution to the revising and editing process of the review manuscript. All of the authors have read and approved the final version of the review manuscript.
